# Recent Advances in the Enantioselective Radical Reactions

**DOI:** 10.3390/molecules28176252

**Published:** 2023-08-25

**Authors:** Tomasz Bauer, Yusuf Zaim Hakim, Paulina Morawska

**Affiliations:** Faculty of Chemistry, University of Warsaw, L Pasteura 1, PL-02-093 Warsaw, Poland; y.hakim@student.uw.edu.pl (Y.Z.H.); pe.morawska2@student.uw.edu.pl (P.M.)

**Keywords:** radical chemistry, enantioselective synthesis, transition metal catalysis, organocatalysis, enzymes

## Abstract

The review covers research published since 2017 and is focused on enantioselective synthesis using radical reactions. It describes recent approaches to the asymmetric synthesis of chiral molecules based on the application of the metal catalysis, dual metal and organocatalysis and finally, pure organocatalysis including enzyme catalysis. This review focuses on the synthetic aspects of the methodology and tries to show which compounds can be obtained in enantiomerically enriched forms.

## 1. Introduction

Radical reactions are now well established as an excellent and one of the most powerful methods for the formation of the carbon–carbon and carbon–heteroatom bonds. Among them, photo-induced transformations are of great value and become very popular since new sources of the light—cheap and efficient LEDs—became available. The key challenge for the radical reaction is the controlling selectivity of the reaction. The highly reactive radical species can be involved in several unwanted side reactions. The problem becomes even more serious in the area of asymmetric synthesis. The synthetic chemist willing to perform radical reactions in a highly enantioselective manner has to not only to control the already mentioned side reactions, but also suppress non-enantioselective background reactions competing with the main process and leading to the formation of racemic products.

Until the late 1990s, the scientific community believed that radical species are too reactive and the control of the stereochemically defined processes is not possible. The pioneering work of Porter and Sibi [1,2] introducing chiral Lewis acids into radical reactions opened new avenues, and in last twenty years, we are seeing an enormous development in the methodology of enantioselective radical chemistry.

Several new methodologies were developed:

- Transition metal catalysis—the catalyst not only creates chiral environment by coordination to a substrate, but also plays important role in the radical generation process;
- Visible light photoredox radical reactions combined with transition metal catalysis;
- Visible light photoredox radical reactions combined with organocatalysis including dual transition metal/organocatalytic processes.

Several excellent reviews have been published [3,4,5,6,7,8,9,10,11,12,13,14,15,16], many of them explaining the mechanistic aspects of the above-mentioned processes in great detail. In this review, we would like concentrate on the synthetic aspects of the methodology leading to enantiomerically enriched compounds. The most valuable and/or informative outcomes of the described reactions will be presented in the schemes. Recent papers published since 2017 are presented.

## 2. Transition Metal Catalysis

### 2.1. Copper-Catalyzed Enantioselective Radical Reactions

**Scheme 1:**
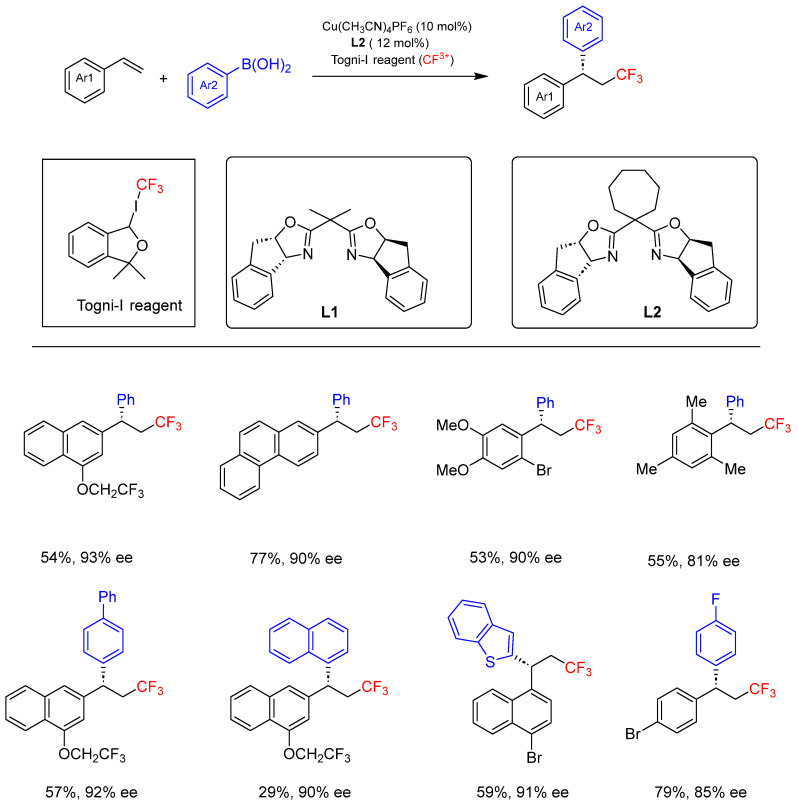
Trifluoromethylarylation of styrenes by enantioselective arylation of benzylic radicals.

Liu’s group developed a concept of transition-metal-catalyzed reactions based on the copper complexes with privileged ligands—bisoxazolines, also known as box ligands. As the early part of asymmetric radical transformations developed by Liu and co-workers, they reported the novel copper-catalyzed intermolecular trifluoromethylarylation of styrenes, in which a variety of CF_3_-substituted 1,1-diarylalkanes could be efficiently synthesized [17]. Several box ligands have been tried and the best results were obtained for two of them, **L1** and **L2**. The process is based on the formation of a benzylic radical-containing CF_3_ group in the cooperative action of the Togni-I reagent, which in the chiral environment of the Cu-box complex leads to an enantioenriched product. The trifluoromethyl group-containing compounds were obtained in good yields and very good enantioselectivities up to 93% ee (Scheme 1). However, the exact mechanism has not been investigated. 

**Scheme 2:**
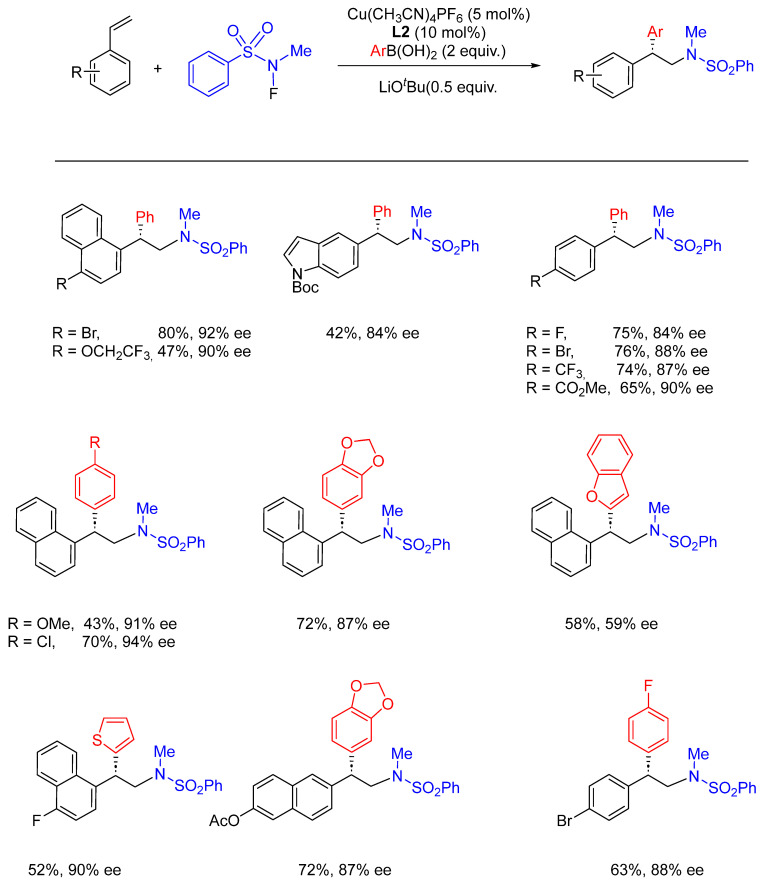
Synthesis of optically active 2, 2-diarylethylamines using N-F reagent and Cu/box complex.

In another paper, the group reported a copper-catalyzed enantioselective aminoarylation of styrenes with specially designed novel N-F reagent, *N*-fluoro-*N*-alkylsulfonamide (NFAS) as the amine reagent generating amino radical. Again, the complex **L1/**Cu(II)Ar was able to control the enantioselective arylation [18]. Several enantioenriched 2, 2 diarylamines were obtained with good yields and very good enantioselectivities, up to 94% ee (Scheme 2). These compounds are a valuable source of biologically active compounds such as dopamine receptors and some anticancer agents.

**Scheme 3:**
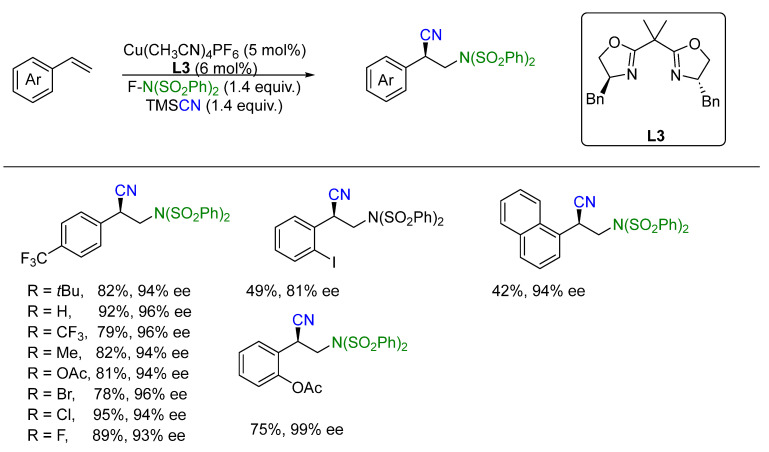
Aminocyanation of styrenes.

Another very interesting method for the difunctionalization of styrenes led to the synthesis of β–amino and β-azidonitriles [19]. In this paper, authors presented an extension of the earlier published methodology: an enantioselective benzylic C-H cyanation, where the formation of a benzylic radical generated by a hydrogen atom transfer (HAT) in the assistance of a chiral bis(oxazoline)/copper cyanide complex leads to enantiomerically enriched benzyl nitriles [20]. The group also recently developed an enantioselective trifluoromethylcyanation reaction of styrene that is initiated by a CF_3_ radical-addition process [21]. Aminocyanation of several substituted styrenes proceeded with very-good-to-excellent enantioselectivities (Scheme 3).

**Scheme 4:**
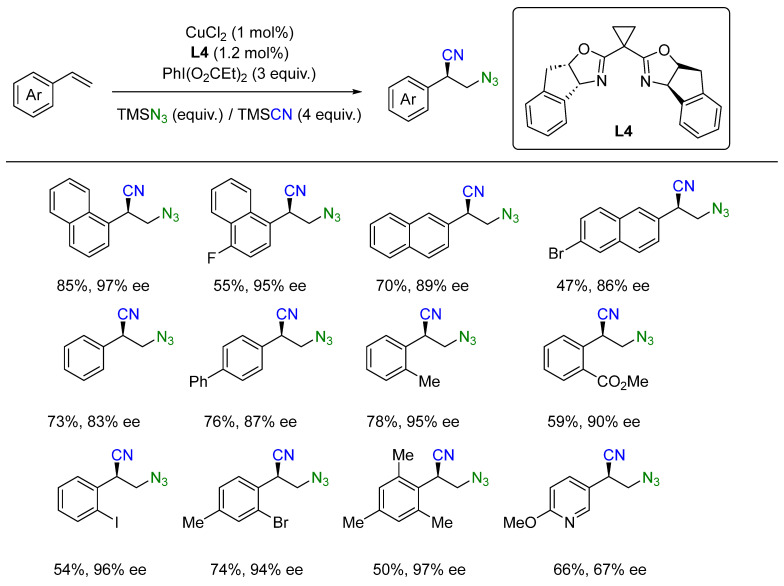
Azidocyanation of styrenes.

The necessity of the sulfonyl group removal under harsh conditions was the major drawback of this approach. Therefore, the authors decided to modify the reaction conditions to introduce an azido group instead of a disulfonyl imino one. In this case, the best results were obtained for indabox **L4**, and also this reaction proceeded with very-good-to-excellent enantioselectivities (Scheme 4). Both electron-donating and electron-withdrawing substituents provided the same level of enantioselectivity, while the pattern of the substitution was important to some extent, *ortho*-substituted substrates delivered the best stereoselectivity. The reaction was not compatible with heteroaromatics, and pyridine derivative was obtained with only moderate ee.

**Scheme 5:**
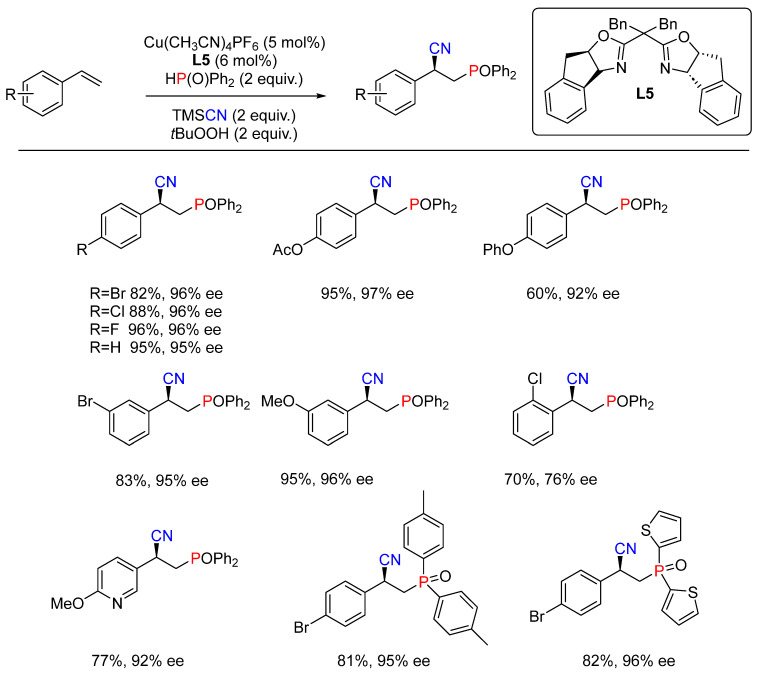
Asymmetric phosphinocyanation of styrenes.

Liu’s group has developed a new method for the asymmetric phosphinocyanation of styrenes based on the proton-coupled electron transfer (PCET). Chiral organophosphines are among most popular ligands used in asymmetric synthesis; therefore, new methods for their synthesis are of great value. In this paper, the authors present the synthesis of the phosphine precursors, a phosphine oxides. The best results were obtained for the reaction catalyzed by the copper complex of the ligand from the indabox family—dibenzyl derivative **L5** [22]. The addition proceeded with both excellent yield and enantioselectivity, and was almost insensitive to the substitution pattern of the starting styrenes (Scheme 5). 

**Scheme 6:**
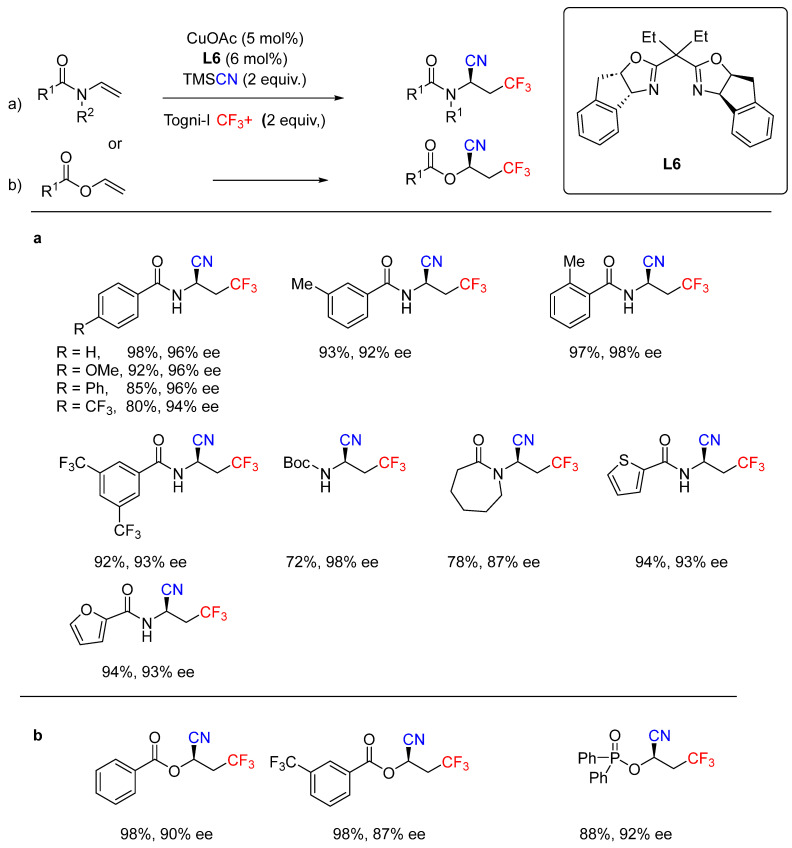
Trifluoromethylocyanation of enamides (**a**) and vinyl esters (**b**).

**Scheme 7:**
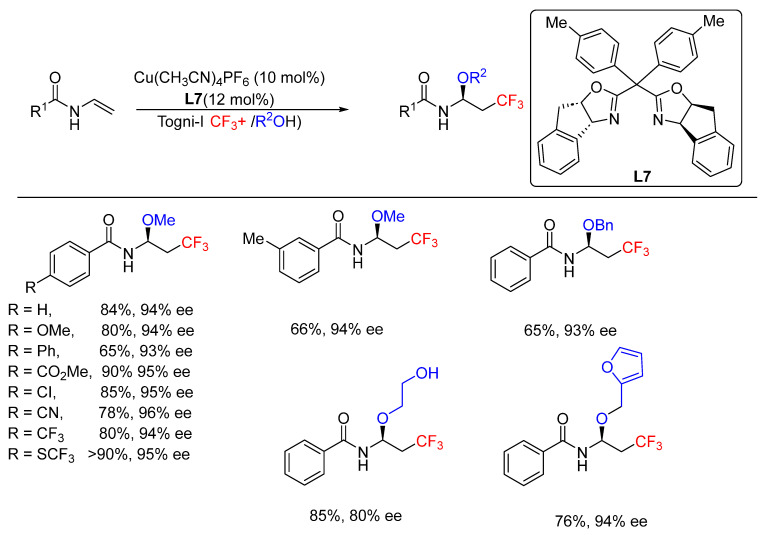
Trifluoromethyloxygenation of enamides.

The methodologies developed by Liu’s group for the difunctionalization of styrenes’ double bond was further extended to more complicated substrates—enamides and vinyl esters [23]. The radical formed on the central carbon of the unsaturated part of the substrate interacts with the copper complex of indabox ligand. The reaction shows high compatibility with various functional groups and proceeds with good yield and very-good-to-excellent selectivities. The reaction is quite flexible and TMSCN can be used to provide trifluoromethylcyanation of enamides and vinyl esters with ligand **L6** (Scheme 6), or the use of alcohol as reagent gave products of trifluoromethyloxygenation with ligand **L7** (Scheme 7).

The major drawback of this approach seems to be necessity of the introduction of a trifluoromethyl group into each product, which narrows the usability of these compounds. 

**Scheme 8:**
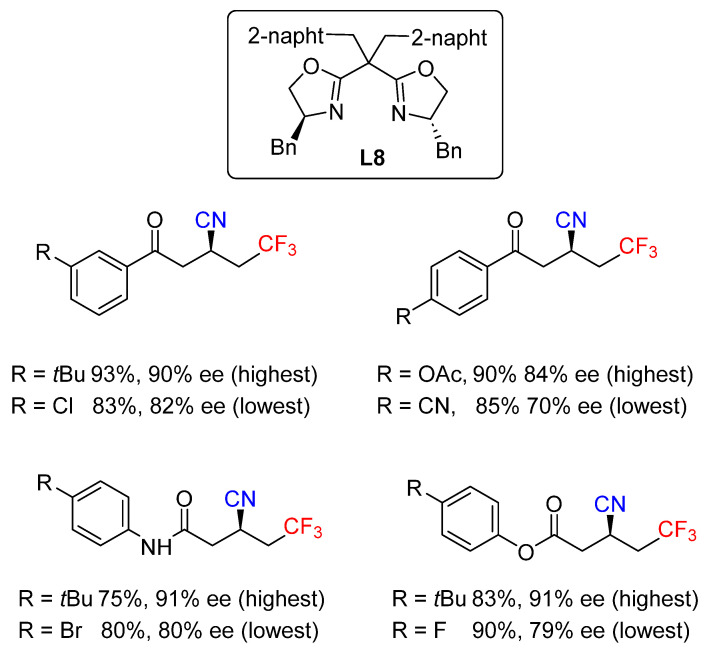
Trifluoromethylocyanation of β,γ-unsaturated carbonyl compounds.

Under the same conditions which were used for the trifluoromethylocyanation of enamides (see Scheme 6), there was the trifluoromethylocyanation of β,γ-unsaturated carbonyl compounds—ketones, esters and amides [24]. In this case, best results were achieved for a box ligand **L8** and the products were obtained with good yields and enantioselectivities in a range of 70–91% ee (Scheme 8).

**Scheme 9:**
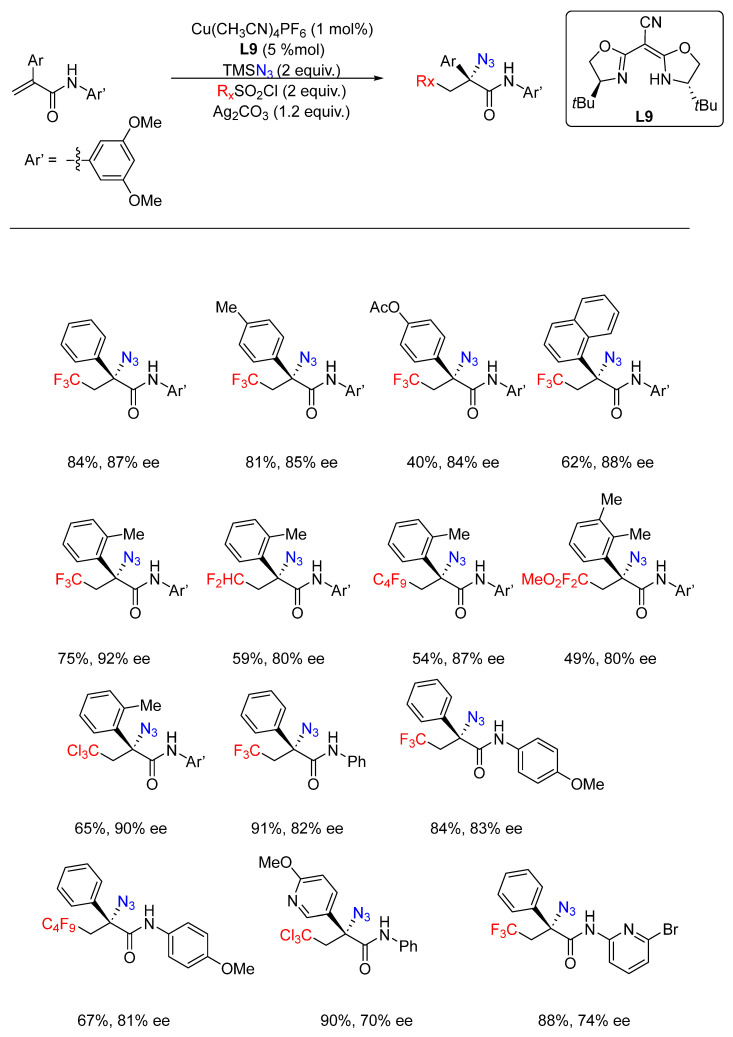
Azidation of acrylamides.

Azidation of acrylamides led to broad range of azides bearing various haloalkyl groups and the best ee’s, up to 90%, were observed for anionic box ligand **L9** [25]. The reactions proceed with good yields and are very tolerant to the various substituents of the double bond and amide’s nitrogen (Scheme 9). Several sulfonyl chlorides, including the range of halosulfonyl chlorides were used as radical precursors with good results; however, simple alkyl sulfonyl chlorides like mesyl chloride failed to react, probably because they were unable to initiate the single-electron-transfer process.

**Scheme 10:**
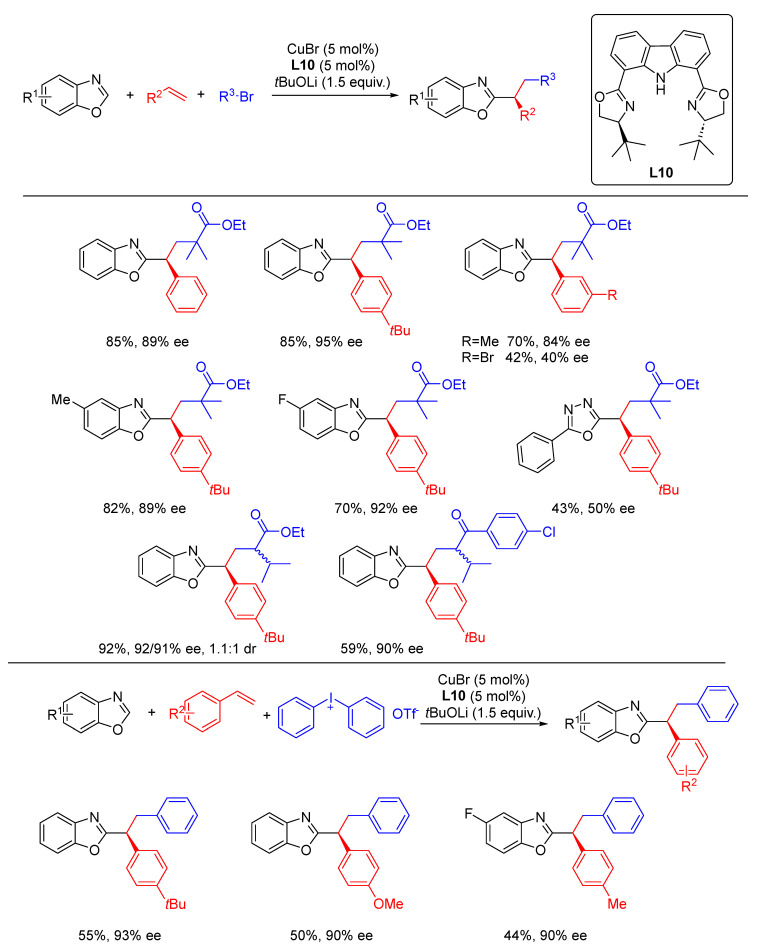
Azolation of alkenes.

The elegant three-component reaction of azoles catalyzed via the copper–bisoxazoline complex was presented by Zhang and Ma [26]. The reactions of various benzoxazoles, styrenes and tertiary bromides or diaryliodonium salts proceeded smoothly with good yields and good-to-very-good enantioselectivities in the presence of carbazol-derived bisoxazoline **L10**. The reactions and some selected examples of the products are presented in Scheme 10.

**Scheme 11:**
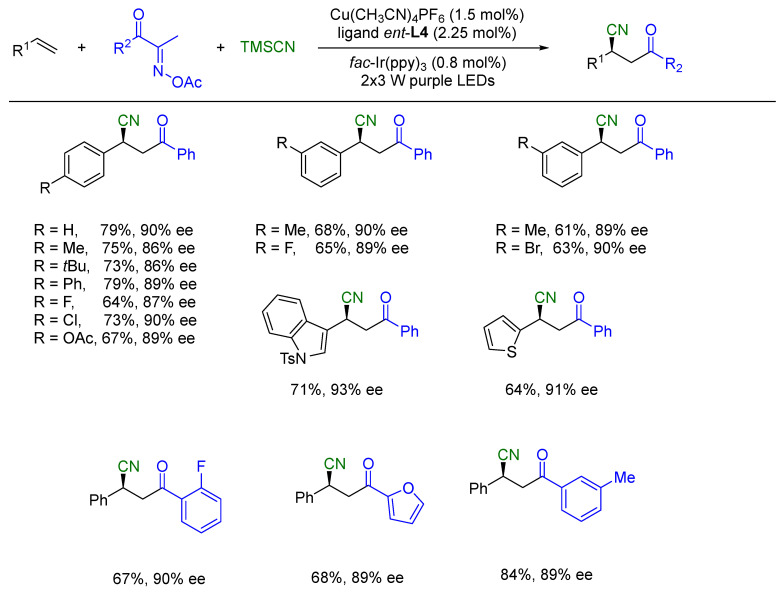
Vicinal alkene difunctionalization.

Another three-component reaction was presented by Wang et al. Intermolecular alkene vicinal dicarbofunctionalization (DCF) is fundamental transformations of alkenes, which allows for the installation of two different carbon fragments. While the ionic version of this reaction is well known, the radical-mediated enantioselective version remained largely unexplored. The author reported a catalytic asymmetric three-component radical vicinal DCF reaction of alkenes with oximes and TMSCN, which provide a general approach of valuable optically active α-cyano ketones and alkyldinitriles [27]. Box ligand *ent*-**L4** provided the highest enantioselectivities in the range 82–93% ee, usually around 90%. The substitution pattern of the tested aryl moieties only slightly influenced the stereoselectivity of the reaction (Scheme 11).

The privileged ligands, bisoxazolines, were broadly used not only for the functionalization of alkenes, but also in several other asymmetric reactions. 

**Scheme 12:**
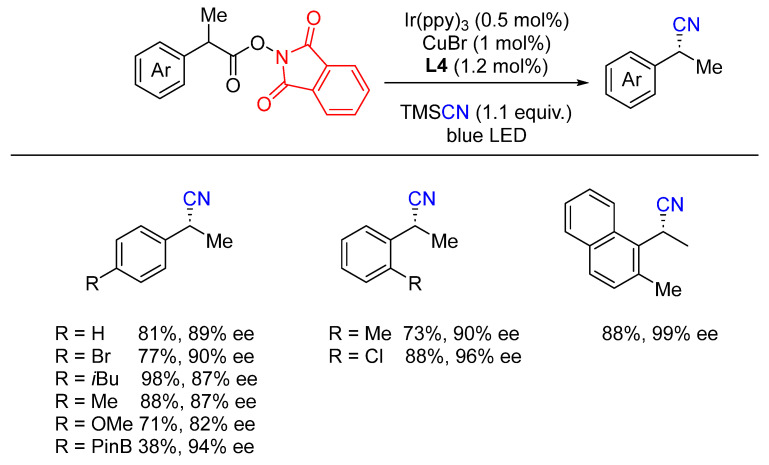
Decarboxylative cyanation.

The ligand **L4** was used in novel process combining photoredox catalysis with asymmetric copper catalysis [28]. The enantioselective decarboxylative cyanation allowed for the synthesis of the broad spectrum of aryl-substituted alkyl nitriles with high yields and with very good and, in some cases, excellent enantioselectivities (Scheme 12). These compounds can be important substrates in organic synthesis and are easily converted into valuable precursors for the synthesis of several important pharmaceuticals.

**Scheme 13:**
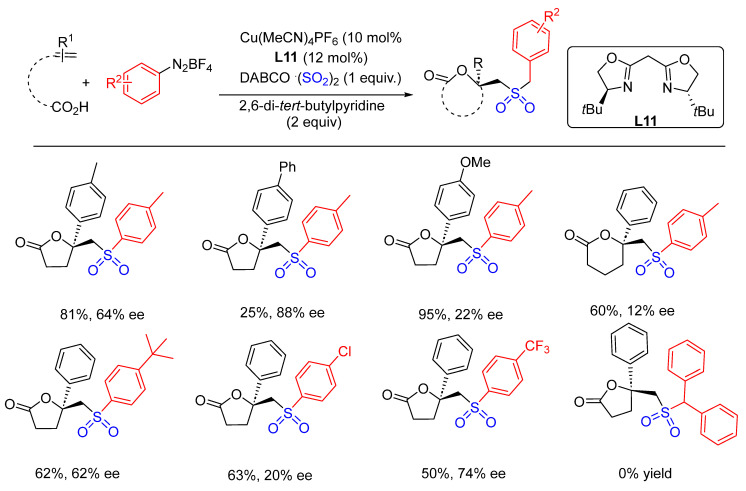
Synthesis of chiral sulfonyl lactones.

Han and co-workers reported novel method for the synthesis of chiral sulfonyl lactones bearing a quaternary stereogenic center based on the copper-catalyzed multi-component reaction of ω-unsaturated carboxylic acids [29]. Lactones were obtained with good yields and moderate selectivities in the presence of classical bu-box ligand **L11** (Scheme 13). However, only three types and five box ligands in total of were taken to the screening procedures; therefore, there is still some room for improvement.

**Scheme 14:**
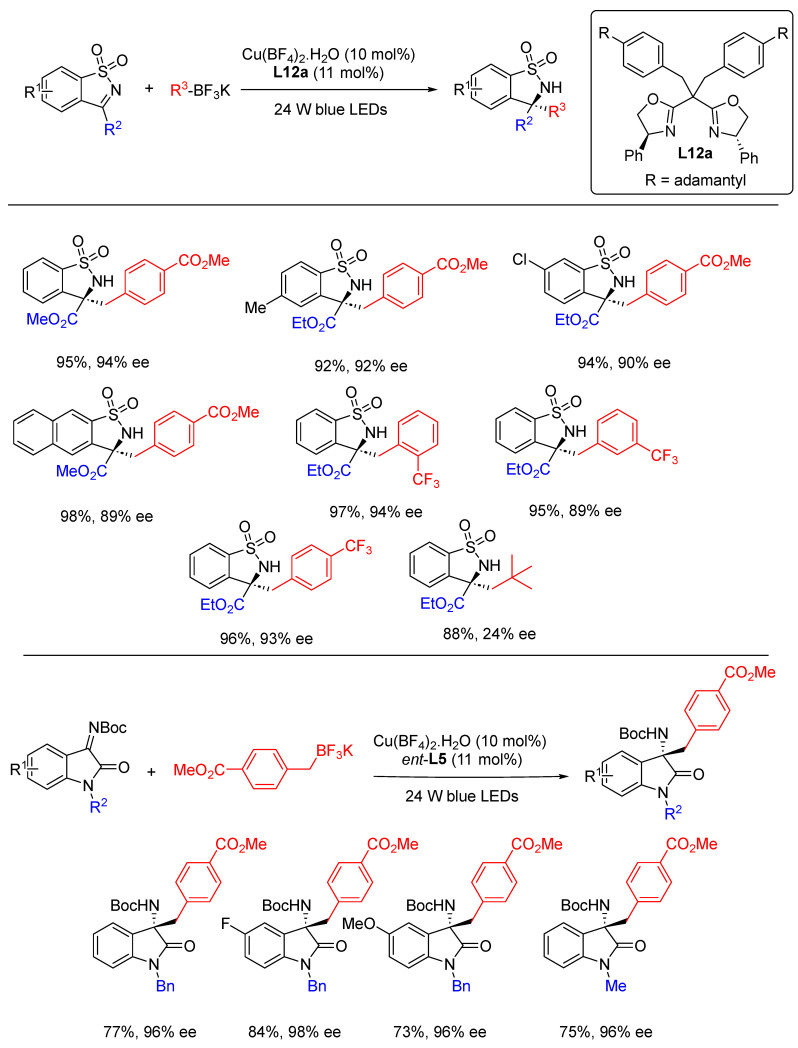
Alkylation of imines and isatin-derived ketimines.

Gong and co-workers reported a highly efficient method for the enantioselective alkylation of imines based on the photoredox/bifunctional Cu(II)/box catalyst [30]. The alkylation of both sulfonyl imines and isatin-derived ketimines proceeded with very-good-to-excellent enantioselectivities, up to 98% ee; however, results were highly dependent on the ligand structure. Several ligands were tested and the best results were obtained using **L12a** for sulfonylimines and *ent*-**L5** for ketimines (Scheme 14). 

**Figure 1:**
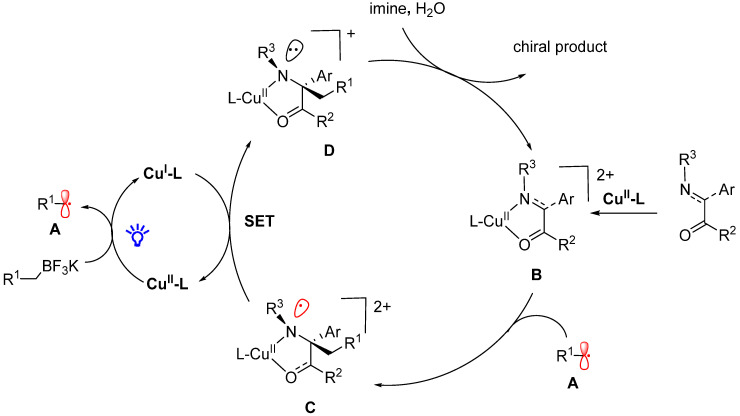
A plausible mechanism for the alkylation of imines and isatin-derived ketimines.

The researchers put in a lot of effort to elucidate the mechanism of the reaction. They probed, inter alia, the radical pathway, investigated UV-VIS spectra and performed cyclovoltametric experiments. The proposed mechanism is presented in Figure 1.

Copper-box complexes were applied to several reactions other than the fuctionalization of the double-bond.

**Scheme 15:**
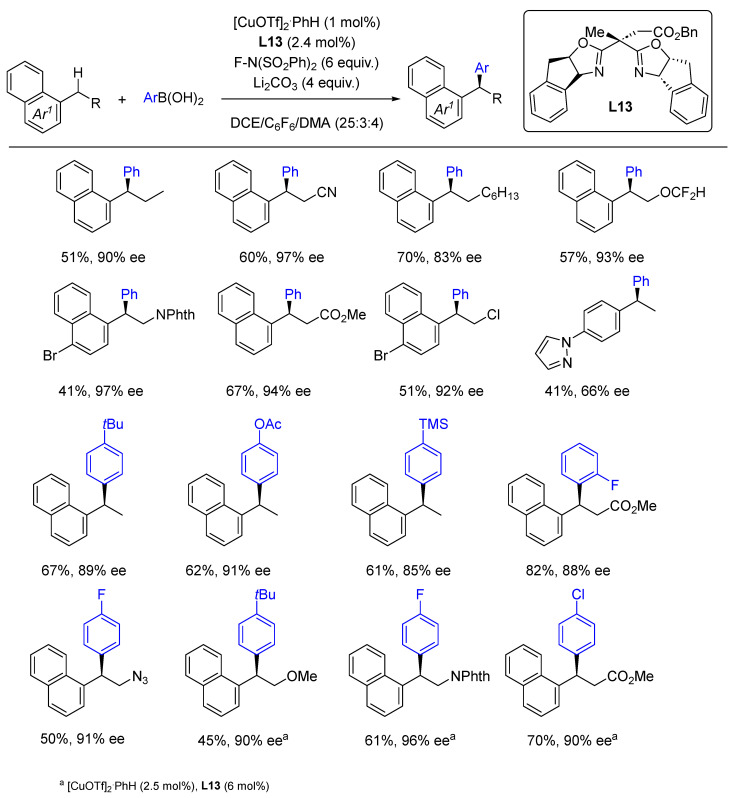
Enantioselective C-H arylation.

Liu et al. reported the benzylic arylation of C(sp^3^)-H bonds in the radical relay process [31]. They showed the importance of the thorough survey of chiral ligands used in this type of the reaction. The structure of the bisoxazolines influences not only the enantioselectivity, but can also increase the reactivity, improve the yield and suppress side reactions. After testing 10 ligands, it was found that the best stereoselectivity and highest yields with only trace amounts of the side products can be obtained in the presence of bisoxazoline **L13**. The reaction is compatible with both electron-donating and electron-withdrawing groups and several functional groups (Scheme 15). 

The first catalytic asymmetric cyanation of propargylic esters was described by Lan, Xiao and co-workers [32].

**Scheme 16:**
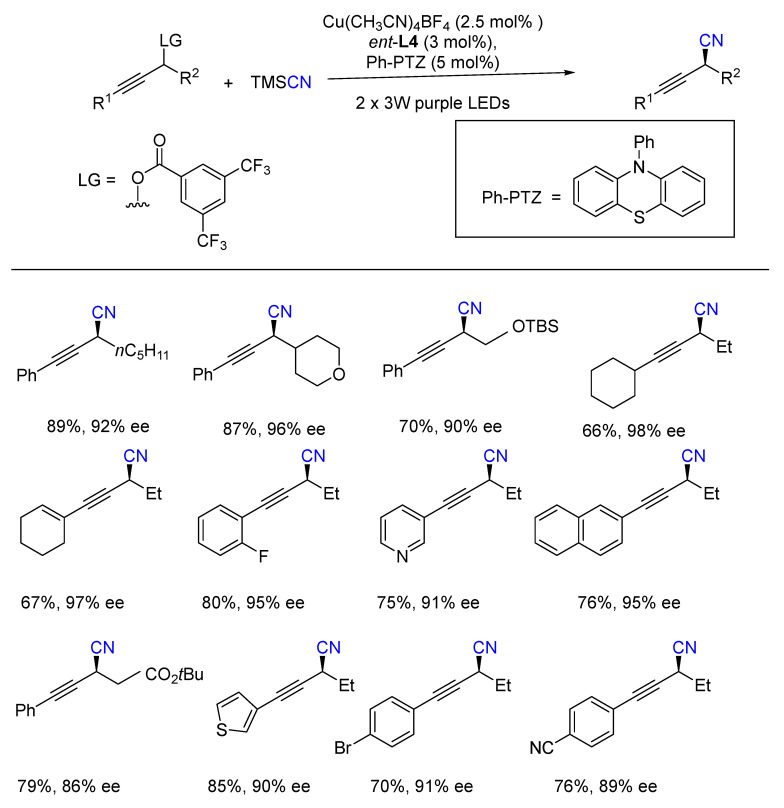
Catalytic asymmetric cyanation of propargyl esters.

They used photoredox catalysis and asymmetric catalysis based on copper complexes of bisoxazoline *ent*-**L4**. The reaction proceeded with good-to-very-good chemical yields and very-good-to-excellent enantioselectivity (Scheme 16). The structure and electronic properties only slightly influenced the outcome of the reaction. Moreover, both photocatalyst (Ph-PTZ, 10-phenyl-10H-phenothiazine) and leaving group 3,5-(ditrifluoromethyl)benzoic acid could be recovered with very good yields.

**Scheme 17:**
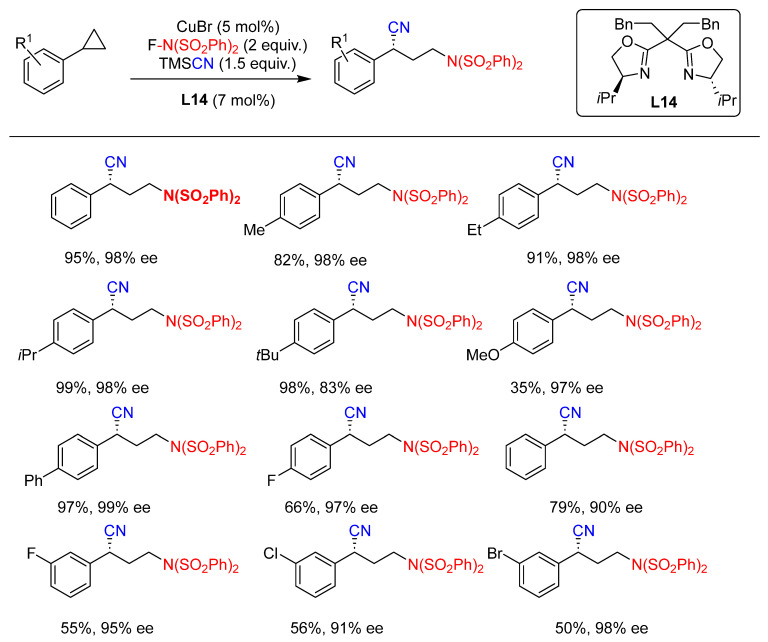
Enantioselective cyclopropyl ring opening.

Yang et al. described highly enantioselective cyclopropyl ring opening of arylcyclopropanes with trimethylsilyl cyanide [33]. The resulting γ-aminonitriles were obtained with good-to-excellent yields and enantioselectivities (Scheme 17).

**Scheme 18:**
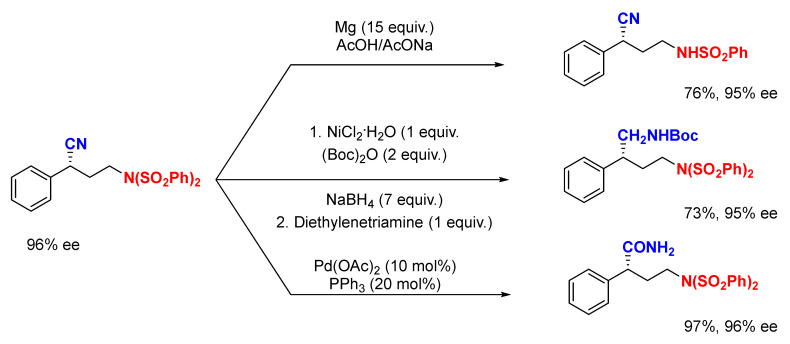
Reactions of γ-aminonitriles.

Products of this reaction can be further converted into diamines or γ-amino amides (Scheme 18); however, the major drawback of this methodology seems to be the harsh conditions for the removal of the sulfonamide protection of the terminal amino group. 

**Scheme 19:**
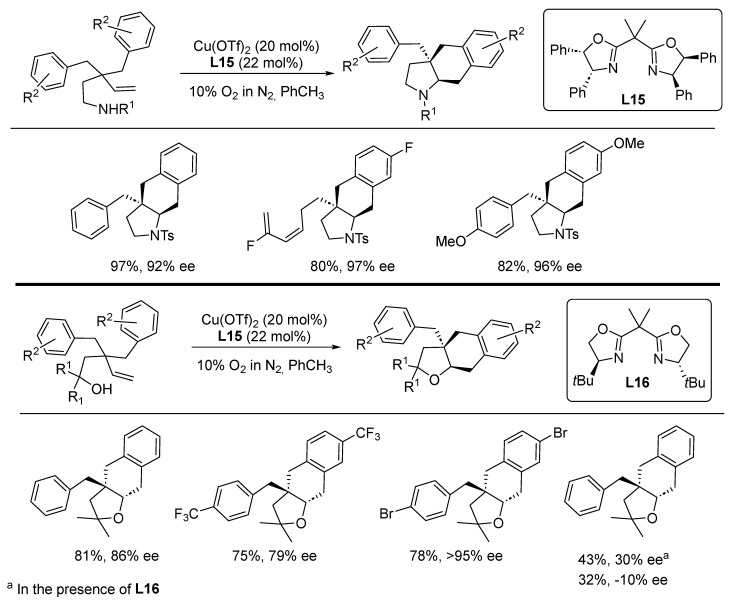
Carboamination and carboetherication of alkenes.

Copper-catalyzed aerobic enantioselective carboamination and carboetherication of alkenes has been reported by Wdowik et al. [34]. Instead of the usually used MnO_2_ or hypervalent iodine reagents for that type of transformation, the authors applied environmentally friendly 10% oxygen (O_2_) in nitrogen as oxidant. Very good results, with ee’s up to 97% ee, were obtained for the carboamination, slightly worse for the carboetherication. The latter reaction gave good results only for sterically demanding tertiary alcohols (Scheme 19). Spirocyclic ethers were also obtained using this methodology, but with low-to-moderate yields.

**Scheme 20:**
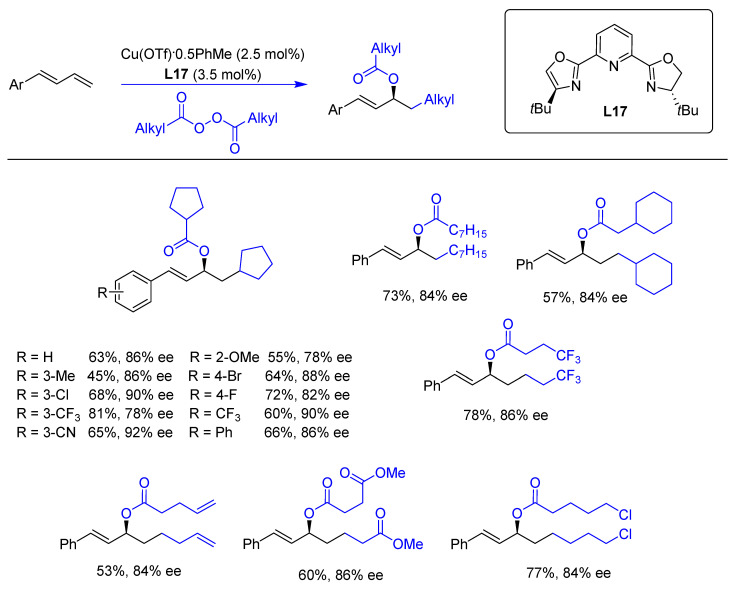
Enantioselective carboesterification of dienes with diacyl peroxides.

Enantioselective carboesterification of dienes with diacyl peroxides leading to chiral acylated α,β-unsaturated alcohols was reported by Zhu et al. [35]. The reaction is catalyzed by a bisoxazoline. Several ligands were checked in order to find the best one for this specific addition, and the best results were obtained for the pybox **L17**; the enantioselectivities were generally in the range 80–90% ee with moderate-to-good yields (Scheme 20).

**Scheme 21:**
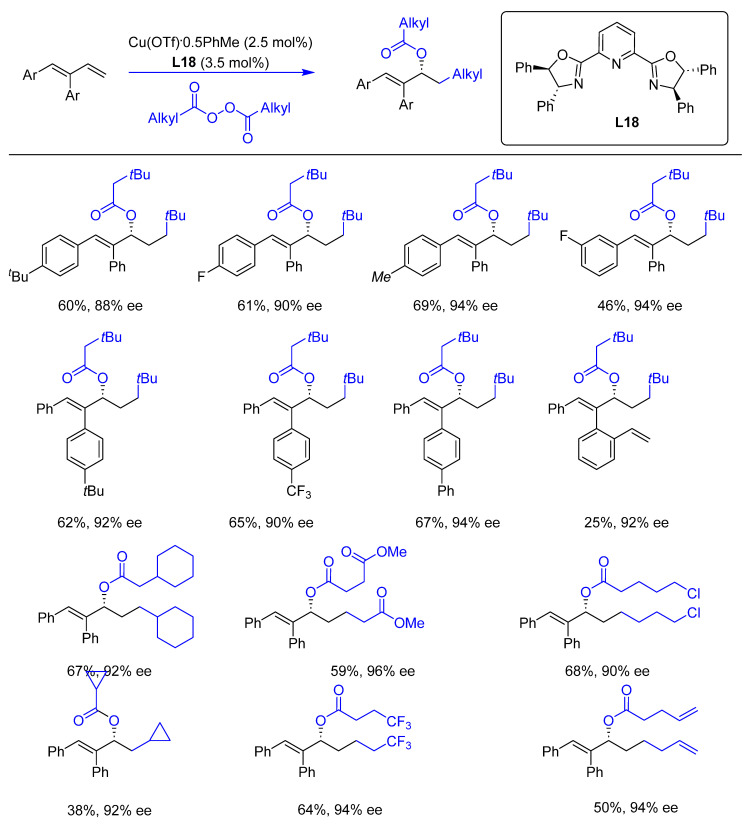
Enantioselective carboesterification of 1,2-disubstituted dienes with diacyl peroxides.

Better enantioselectivities, usually over 90% ee, were observed for the disubstituted dienes; however, the bulkier pybox ligand **L18** had to be used. Moreover, the reaction was fully regioselective for 1,2-diaryl dienes (Scheme 21).

**Scheme 22:**
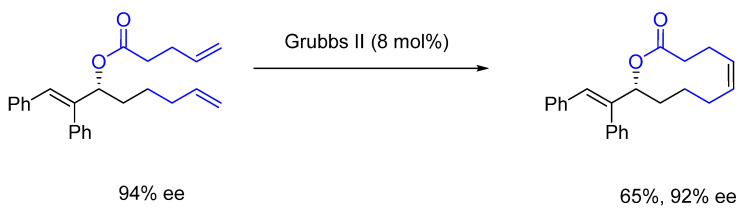
Ring-closing metathesis leading to 10-membered ring.

The products of this reaction can be useful starting material for further transformations, as was shown by the RCM reaction in Scheme 22.

**Figure 2:**
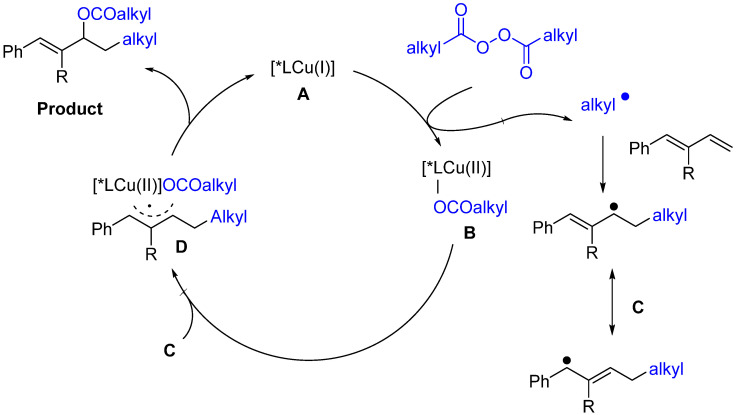
A proposed mechanism for the carboesterification of dienes.

The mechanism studied under radical clock reaction, radical trap experiment, crossover reaction with two different peroxides, crystallography of two single crystals of dimer copper-dimer ligand complex, and MS studies of copper complex resulted in the plausible mechanism below in Figure 2.

**Scheme 23:**
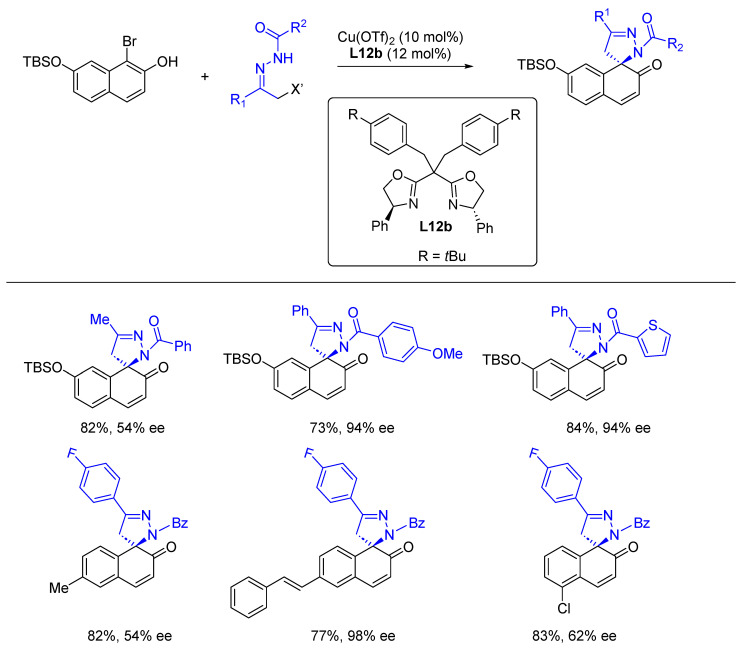
Dearomatization/dehalogenations of β-naphtols.

The asymmetric Cu-catalyzed [4+1] spiroannulation reaction has been reported by Bai et al. [36]. Dearomatization/dehalogenation of α-bromo-β-naphtols produced pyrazoline-based frameworks with moderate-to-excellent enantioselectivity (Scheme 23). 

**Scheme 24:**
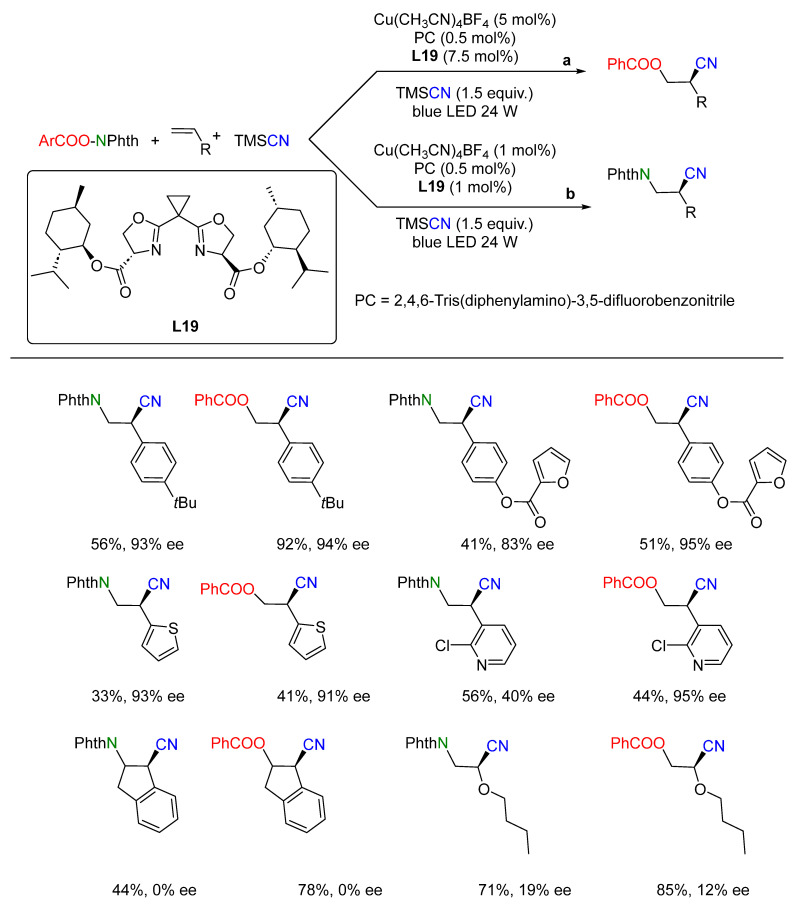
Chemodivergent asymmetric aminocyanation and oxocyanation of alkenes.

A very interesting approach to the enantioselective difunctionalization of alkenes using dual photoredox and copper catalysis was developed by Lu and co-workers [37]. They found that the generation of either an *O*-centered aroyloxy radical or an *N*-centered phtalimidyl radical from *N*-(aroyloxy)-phtalimides (ArCO_2_NPhth) depends on the ratio of the ligand to the copper catalyst. The photocatalyst generates an *O*-centered aroyloxy radical in the presence of excess copper and *N*-centered phtalimidyl radical when the ligand predominates over copper. Several box ligands have been investigated and the best results were obtained for the new serine-derived, (-)-menthol substituted ligand **L19**. The reaction proceeded with good yields and very-good-to-excellent enantioselectivities, albeit with some important exceptions. Representative examples are presented in Scheme 24.

**Scheme 25:**
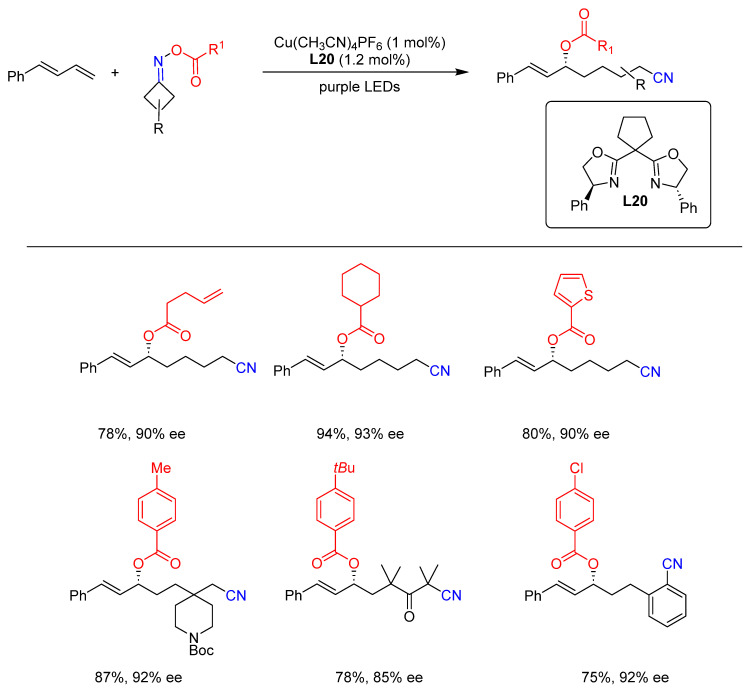
C-O cross-coupling.

The very interesting approach to oxocyanation was presented by Chen et al. [38]. The nitrile group is formed by a ring-opening of cycloalkenone’s acylated oxime with the simultaneous C-O cross-coupling with 1-Ar-substituted 1,3-dienes leading to chiral allylic esters bearing terminal CN moiety. In the presence of the simple box ligand **L20**, the reaction proceeds with high regioselectivity, very good yields and very-good-to-excellent enantioselectivities (Scheme 25).

**Figure 3:**
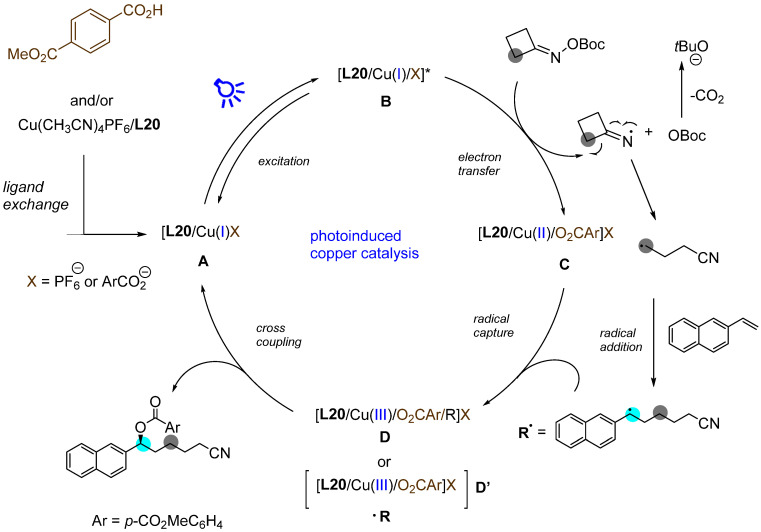
Mechanism of C-O cross-coupling.

Mechanistic studies involving both experiments and DFT calculations led to the proposition of the plausible mechanism (Figure 3).

**Scheme 26:**
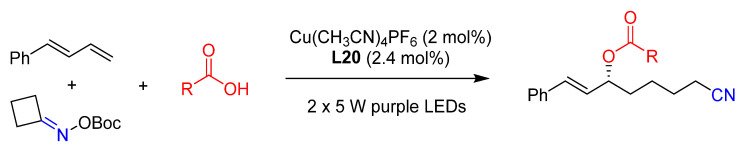
Three-component C-O cross-coupling.

A modified version of that research, using Boc protected oximes and carboxylic acids as the third component, was simultaneously published by Chen’s group (Scheme 26) [39].

**Scheme 27:**
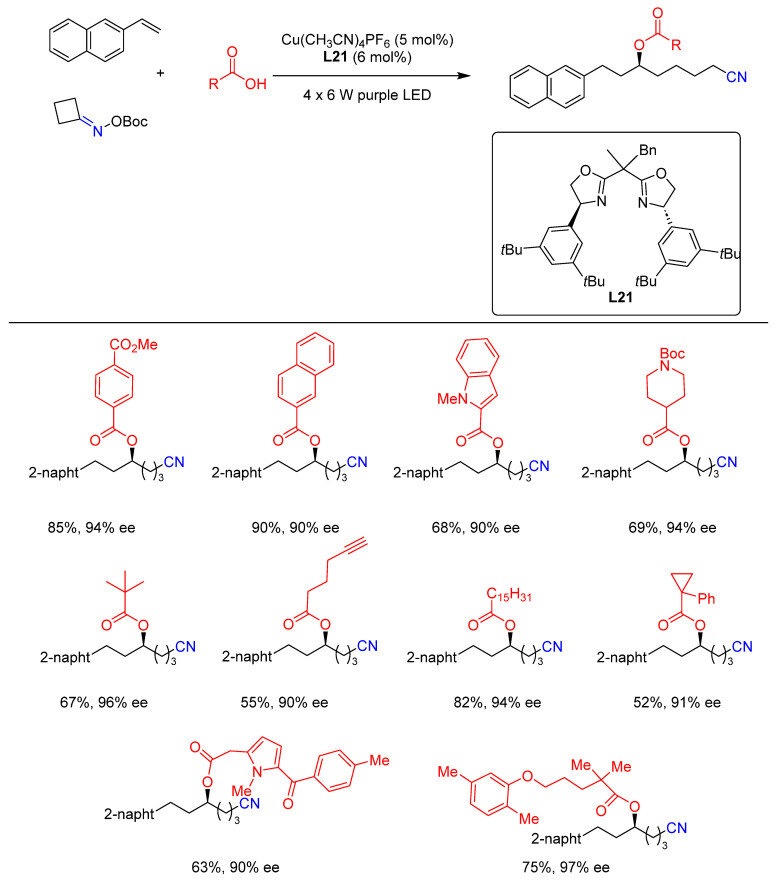
C-O coupling of 2-vinylnaphtalenes.

**Scheme 28:**
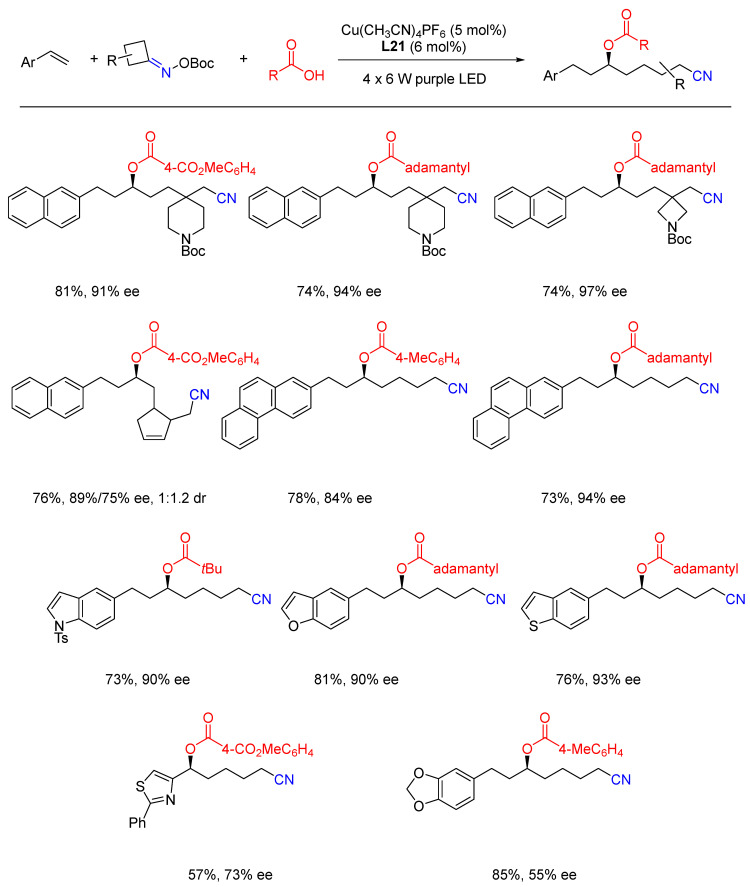
C-O coupling of various oxime esters and (hetero)arenes.

The atom transfer radical addition approach was successful for arylalkenes, albeit optimization of the reaction required thorough studies on the bisoxazoline structure. Finally, **L21** was shown to give the best results for various carboxylic acids and alkenes with good-to-excellent enantioselectivities, as shown in Scheme 27 and Scheme 28 [40]. 

**Scheme 29:**
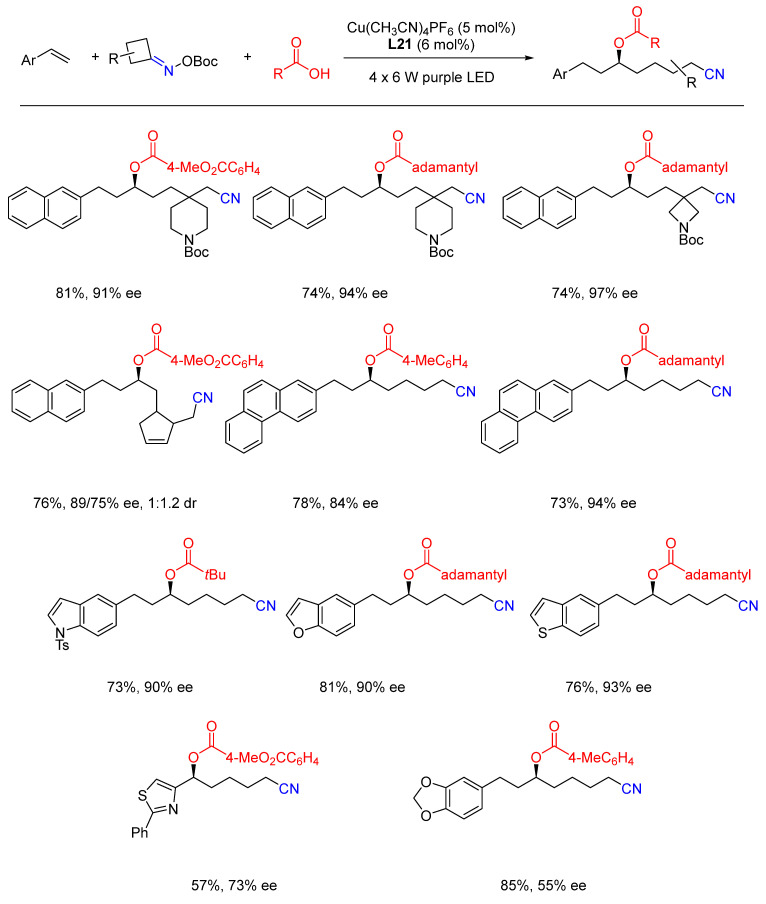

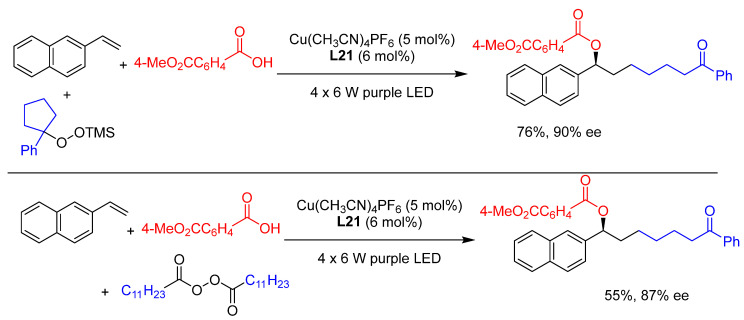
C-O coupling of carboxylic acids and styrenes.

Under those conditions the functionalization of alkenes using peroxides as a radical precursor provided highly enantioenriched products (Scheme 29). 

**Scheme 30:**
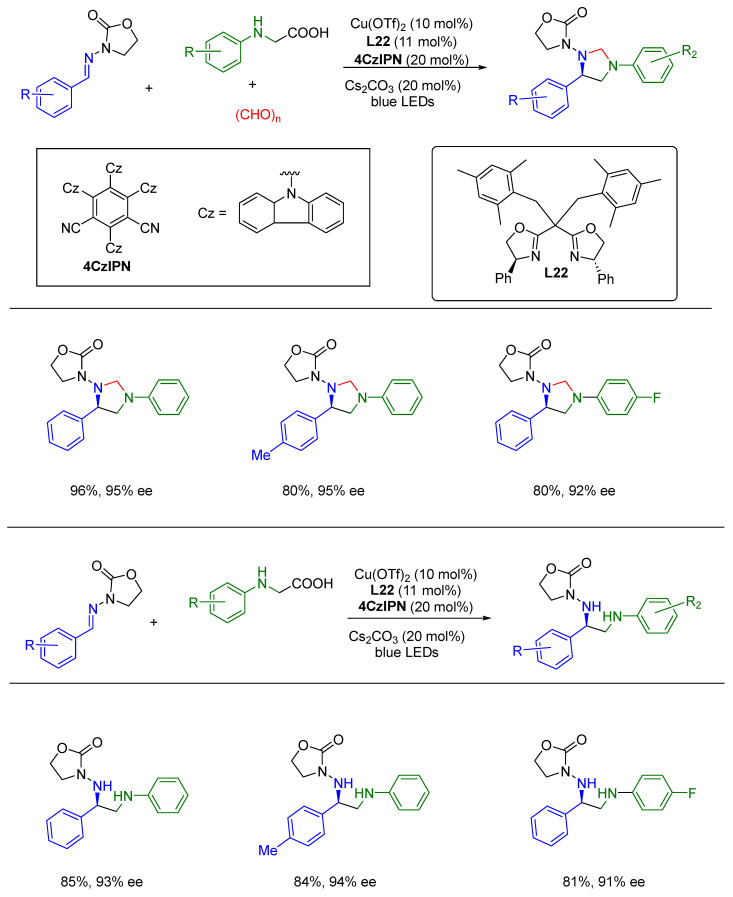
Synthesis of imidazolidines catalyzed by copper complex of **L22.**

The method for the synthesis of imidazolidines catalyzed by copper complex with bisoxazoline **L22** was developed by Dai et al. [41]. The combination of copper catalysis and visible-light-induced photocatalysis in the presence of photosensitizer 4CzIPN allowed for the synthesis of imidazolidinones with very good yields and enantioselectivities. This approach is quite flexible, and when the three-component reaction leads to the synthesis of imidazolidines, the same reaction in the absence of formaldehyde gives vicinal diamines with comparable yields and selectivities (Scheme 30).

**Scheme 31:**
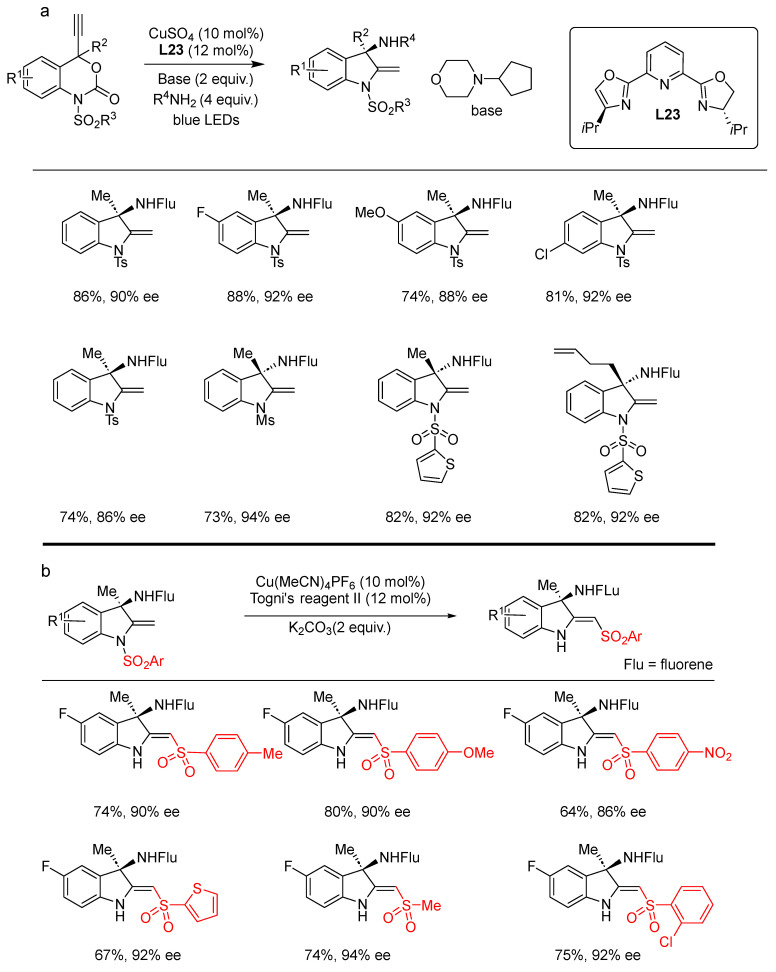
The synthesis of 3-aminoindolines and their sulfonyl derivatives.

The synthesis of 3-aminoindolines (compounds with potential antitumor activity) based on the Cu-catalyzed enantioselective cyclization of ethynyl benzoxazinones with amines using simple pybox ligand **L23** (or its enantiomer) was reported [42]. The reactions proceeded smoothly to give 3-aminoindolines. The model reactions were performed mainly with 9H-fluoren-9-amine, but also with some other aromatic amines and a broad spectrum of sulfonamides, as well as alkyl substituents, providing 3-aminoindolines in very good yields and enantioselectivities (Scheme 31a).

The attempt to introduce a trifluoromethyl group to terminal alkene carbon using Togni’s reagent led unexpectedly to sulfonyl migration with good yields (Scheme 31b).

**Scheme 32:**
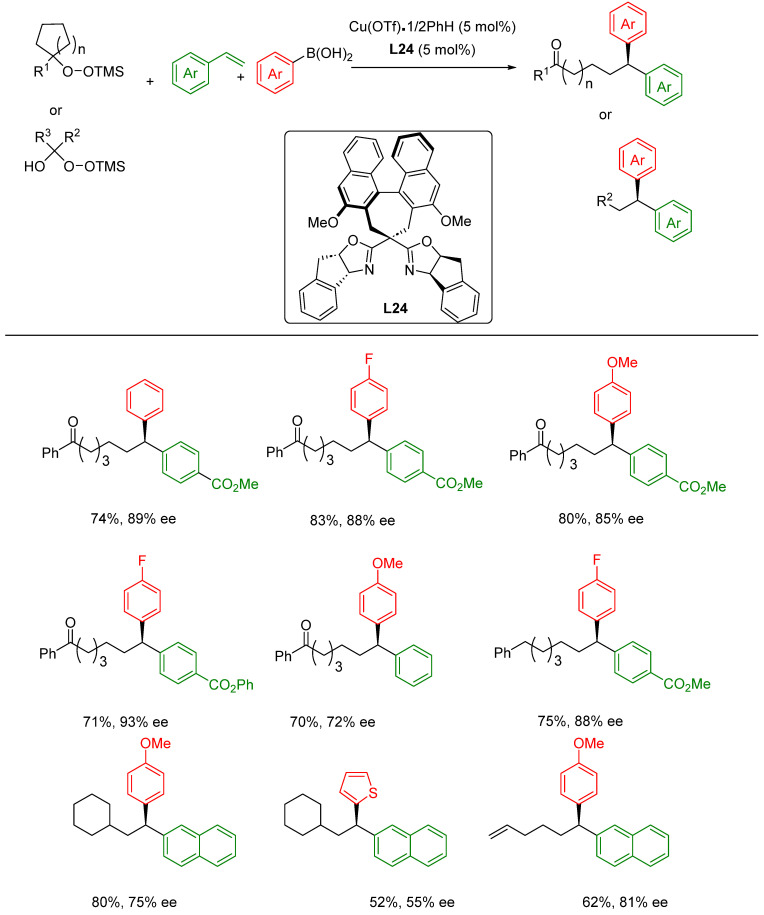
Synthesis of chiral 1,1-diarylalkanes.

Synthesis of chiral 1,1-diarylalkanes in radical relay coupling reactions was presented by Maruoka and co-workers [43]. In order to achieve an acceptable level of enantiodiscrimination in this three-component coupling of alkylsilyl peroxides, vinylarenes, and arylboronic acids, series of a new type of hybrid binaphtol-box ligands, were designed and the best results were obtained for the ligand **L24** (Scheme 32); however, mostly moderate-to-good enantioselectivities were observed. 

**Scheme 33:**
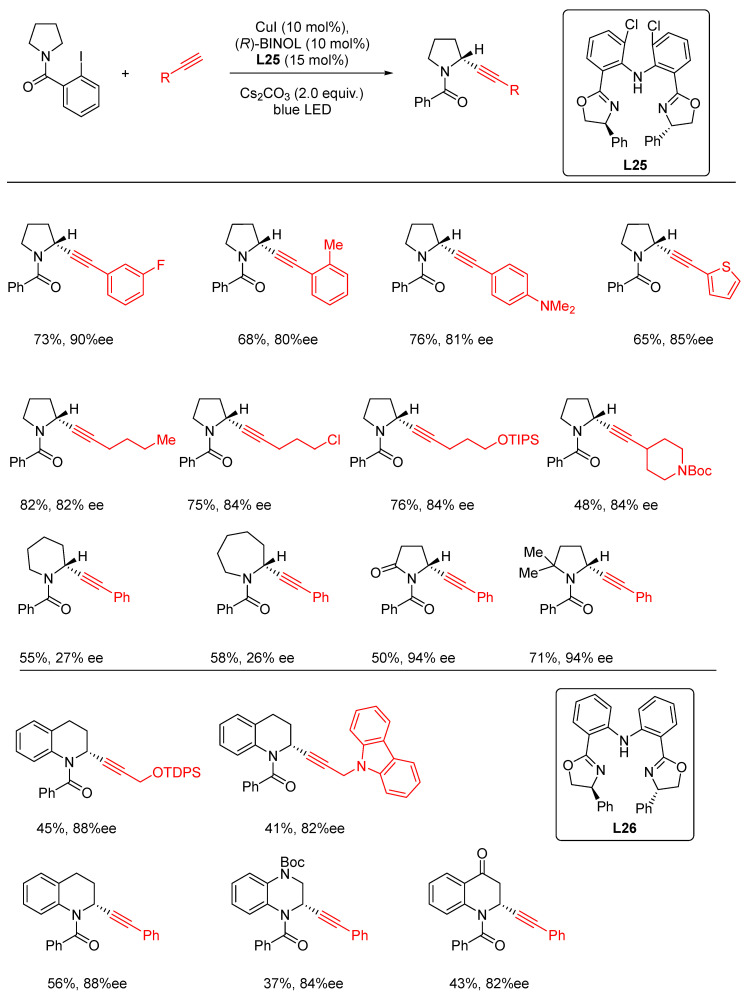
Alkynylation of cyclic, unactivated amines.

Enantioselective α-C(sp^3^)-H-alkynylation of cyclic, unactivated amines were successfully performed yielding chiral propargylic amines with enantioselectivities over 80% ee, and in some case over 90% ee [44]. This level of enantiodiscrimination was achieved after thorough studies on the box ligand structure–ee relationship, and after the important discovery that BINOL is an indispensable element of the system. In the Maruoka’s studies (vide supra) binaphtol had to be a part of the ligand. Herein, only the presence of the optically pure BINOL as additive to a ligand **L25** was sufficient to highly improve chemical yield and, to some extent, the enantioselectivity (Scheme 33).

In the same publication, authors perform diastereoselective additions to d- and l-proline and report mismatched interactions between chiral substrate and chiral catalyst; however, no investigation into which elements in this three-molecule (substrate-BOPA ligand **L25**-(*R*)-BINOL) system are responsible for that effect were conducted. 

Under the same conditions, but in the presence of ligand **L26**, benzo-fused amines were also alkynylated with very good enantioselectivities up to 88% ee.

**Figure 4:**
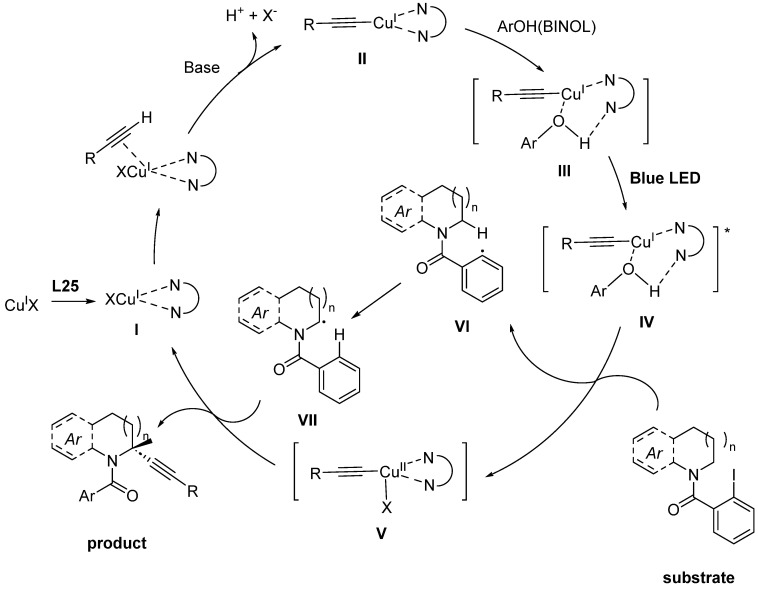
Mechanism of amine alkynylation.

The mechanism was comprehensively studied with (a) a control experiment of possible intermediates, (b) reduction potential of substrates and different complexes, (c) Stern-Volmer plots, (d) deuterium-labeling and KIE experiments, (e) TEMPO trapping experiment, (f) stereocontrol effect of BINOL, and (g) SAESI-MS of possible intermediates, which resulted in the plausible mechanism presented below (Figure 4).

Other interesting ligands used in enantioselective reactions are phosphino-oxazolines (PHOX ligands). These well-known ligands, which proved their efficacy in several types of enantioselective processes were recently applied for copper-catalyzed radical reactions. Liu et al. successfully used them in the radical oxidative C(sp^3^)-C(sp) cross-coupling of C-H bonds with alkynes [45]. 

**Scheme 34:**
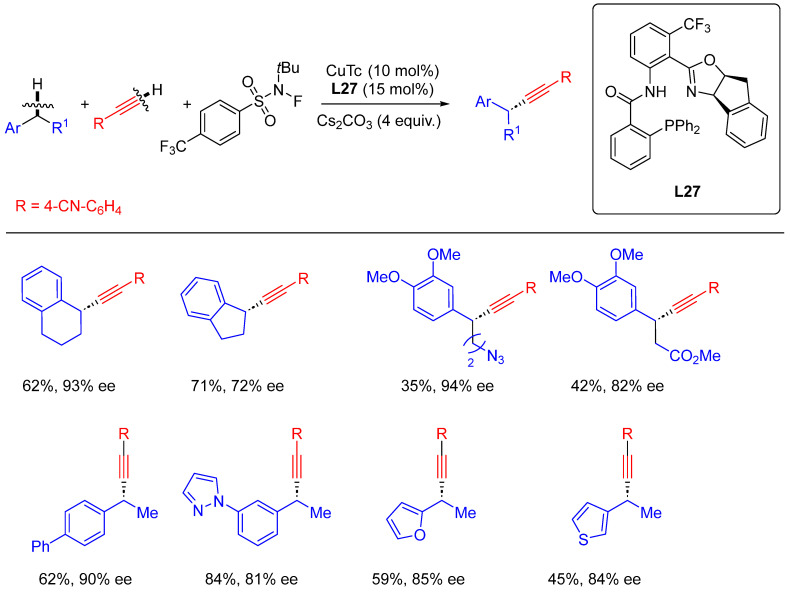
Oxidative C(sp^3^)-C(sp) cross-coupling of C-H bonds with alkynes.

**Scheme 35:**
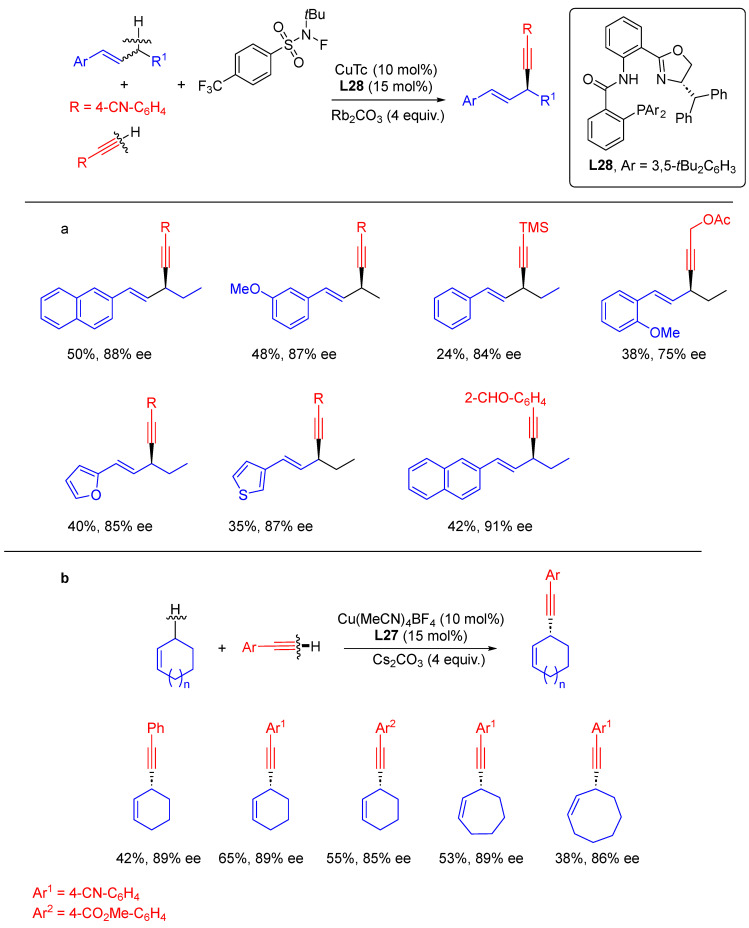
Oxidative C(sp^3^)-C(sp) cross-coupling of C-H bonds with alkynes.

We should consider it as a rule, that for the given enantioselective reaction, the structure of the ligand has to be fitted to the reagents in order to provide the best asymmetric induction. So, tedious studies including several ligands of different structure and mode of action have to be performed in most cases. Authors found that highest ee’s for C-H functionalization of benzylic position as well as of the allylic position in cyclic alkenes is secured by the use of ligand **L27** in the presence of *N*-fluorosulfonamide as an oxidant (Scheme 34 and Scheme 35b), while **L28** is the best one for acyclic alkenes (Scheme 35a).

**Scheme 36:**
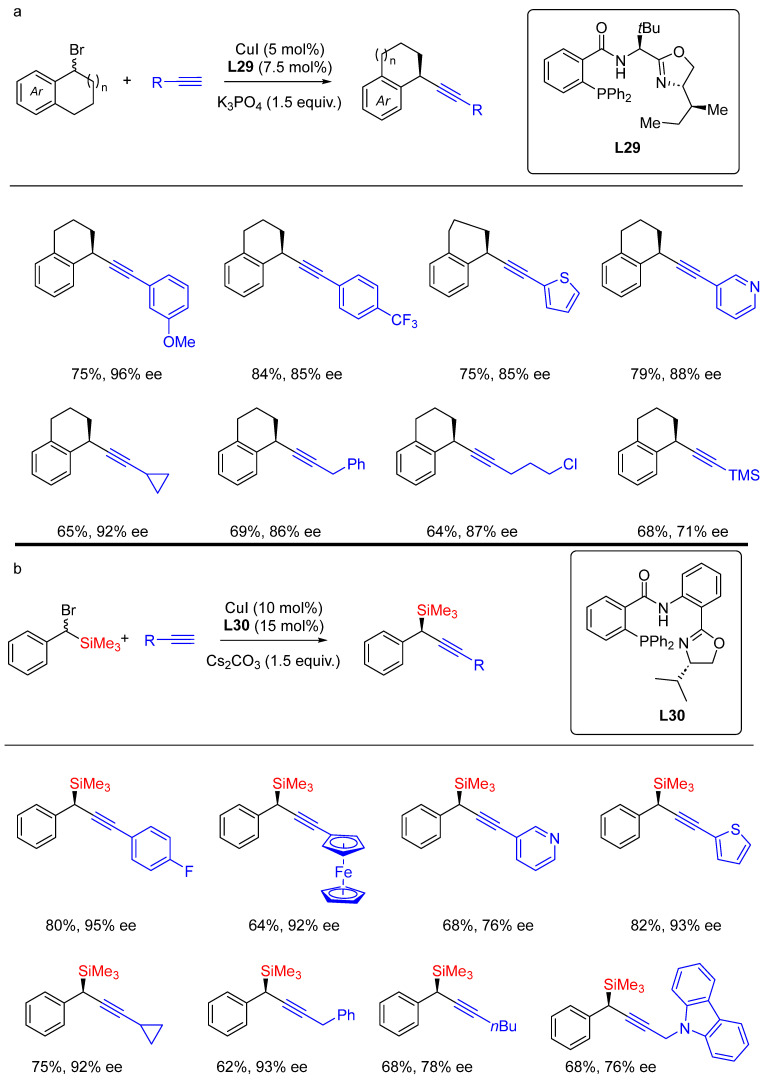
Alkynylation of benzylic bromides.

A different approach to the installation of an alkynyl moiety in the benzylic position was proposed by Zhang’s group [46]. They performed alkynylation of various benzylic bromides (Scheme 36a), including α-silyl bromides (Scheme 36b), and achieved very good yields and enantioselectivities, in a few cases as high as 96% ee in the presence of two novel PHOX ligands, **L29** and **L30**.

Cinchona alkaloid derivatives, extremely popular in organocatalysis, and to a lesser extent in transition metal catalysis and organometallic chemistry, were used as ligands for radical reactions.

**Scheme 37:**
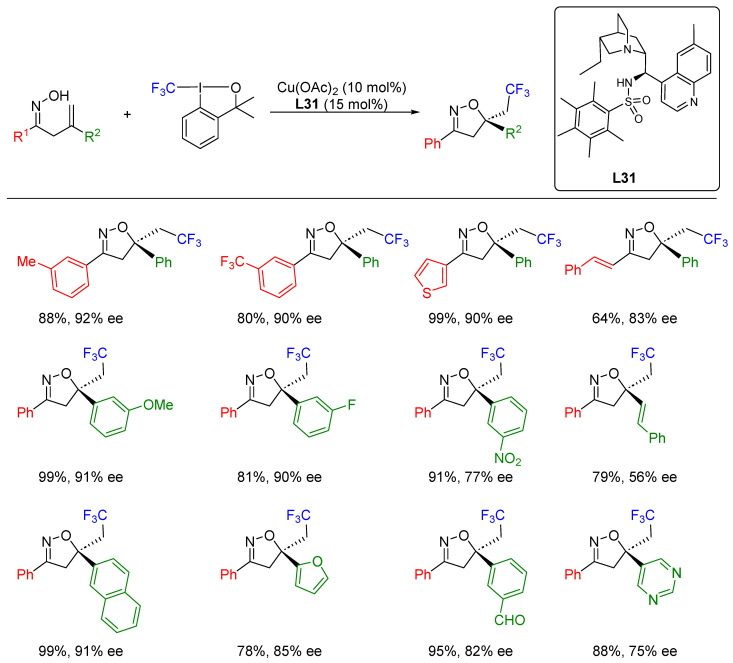
Asymmetric radical oxytrifluoromethylation of oximes.

Li et al. developed an asymmetric radical oxytrifluoromethylation of oximes. They have checked several cinchona derivatives for efficient copper catalysis and found, that in the presence of the cinchonine sulfonamide **L31**/copper complex, CF_3_-containing isooxazolines bearing α-tertiary stereocenters were obtained with good-to-excellent yield and enantioselectivity [47]. Selected results are presented in Scheme 37.

**Figure 5:**
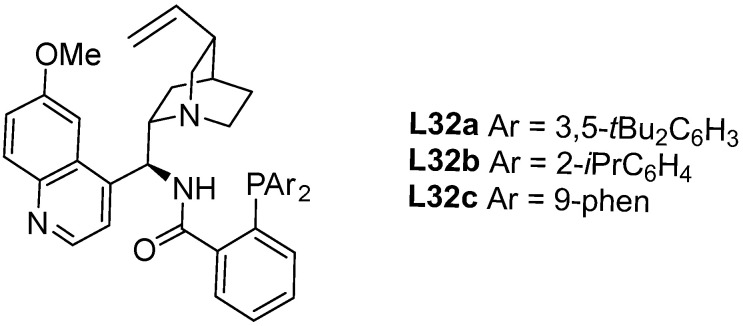
Cinchona alkaloid ligands.

New chiral ligands based on the modified cinchona alkaloids were developed by Dixon’s group [48] and later were successfully (after some modifications) used by Liu’s group; their common feature was the presence of triaryl phosphine moiety, thus taking advantage of the Cu-P soft–soft interactions. The three most efficient ones are presented in Figure 5.

**Scheme 38:**
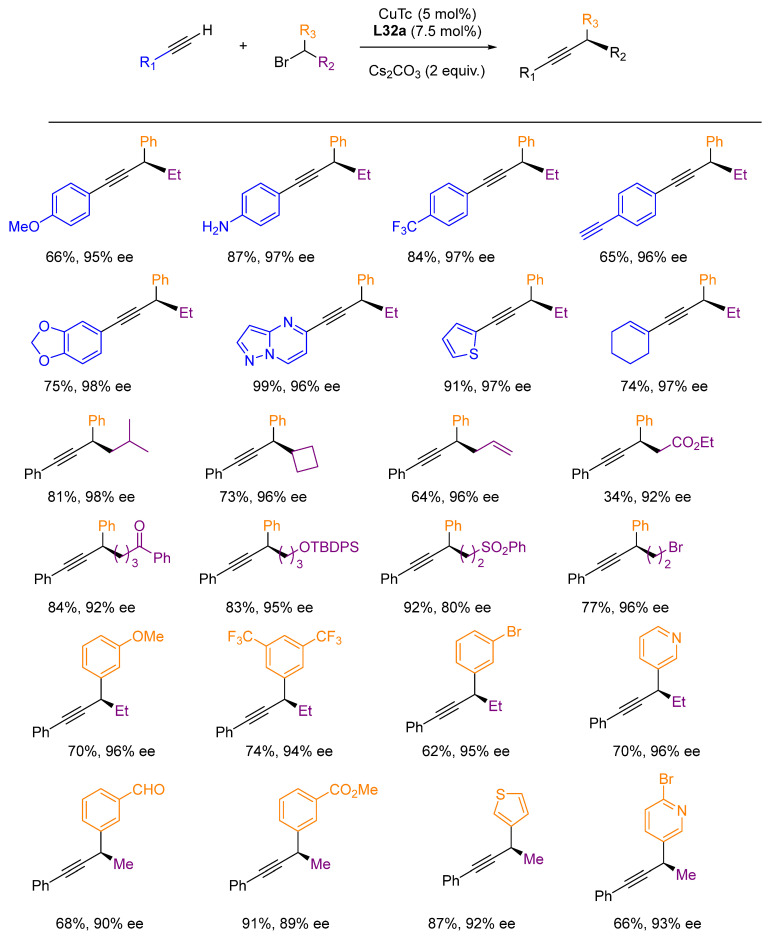
Sonogashira C(*sp^3^*)-C(*sp*) cross-coupling of alkynes with racemic alkyl bromides.

Liu and co-workers reported the enantioconvergent Sonogashira C(*sp^3^*)-C(*sp*) cross-coupling of alkynes with racemic alkyl bromides using a copper complex of a cinchona alkaloid-based *P,N*-ligand **L32a** [49]. Several ligands from various classes, as phosphoramidites, BOX and PHOX ligands, axially chiral phosphines and cinchona alkaloid derivatives were tried, and the best results were obtained for **L32a**. This system appeared to be relatively insensitive to the substituents of the reaction partners and tolerant of various functional groups; very good yields and very-good-to-excellent enantioselectivities were reported (Scheme 38).

**Scheme 39:**
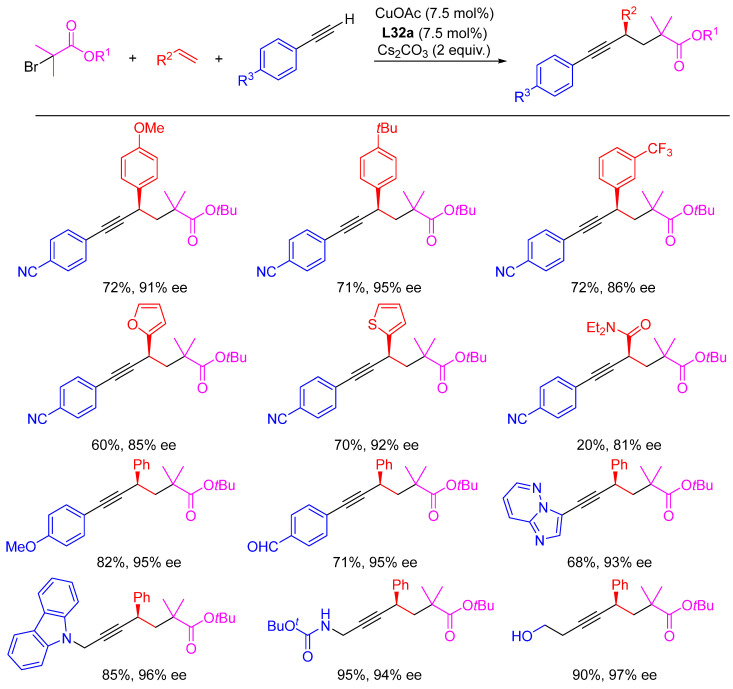
1,2-Carboalkynylation of alkenes.

Liu’s group used ligand **L32a** in another alkynylation reaction, namely enantioselective 1,2-carboalkynylation of alkenes [50]. The reaction proceeded very well for a variety of terminal alkenes, alkynes as well as several alkyl halides, and high chemical yields and enantioselectivities were obtained (Scheme 39).

**Figure 6:**
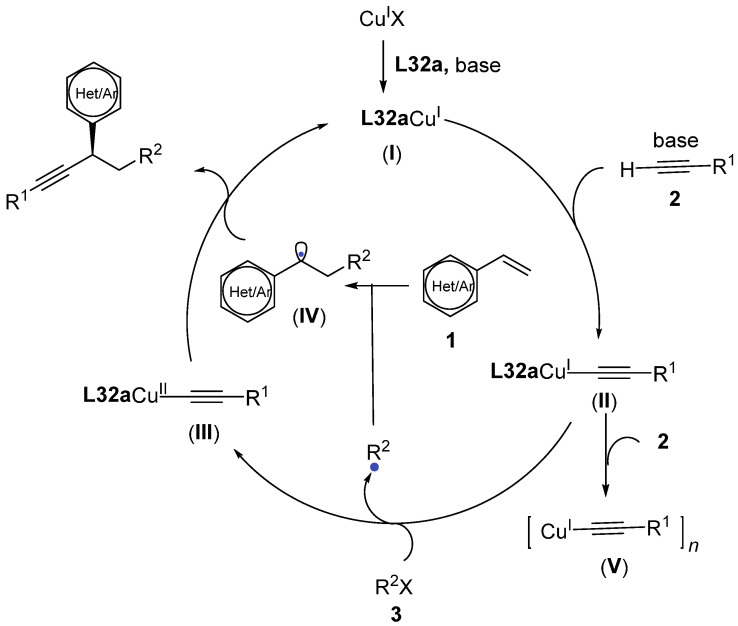
Mechanism of 1,2-carboalkynylation of alkenes.

The comprehensive mechanism was studied by control experiments, radical clock and radical quenching experiments, NMR studies of **L32a**-Cu complex, cyclic voltammograms to investigate the redox potential of Cu(I) catalyst, and EPR experiments to support the involvement of a Cu(II) species, and lastly the kinetic analysis. On that basis, a plausible mechanism was proposed (Figure 6).

**Scheme 40:**
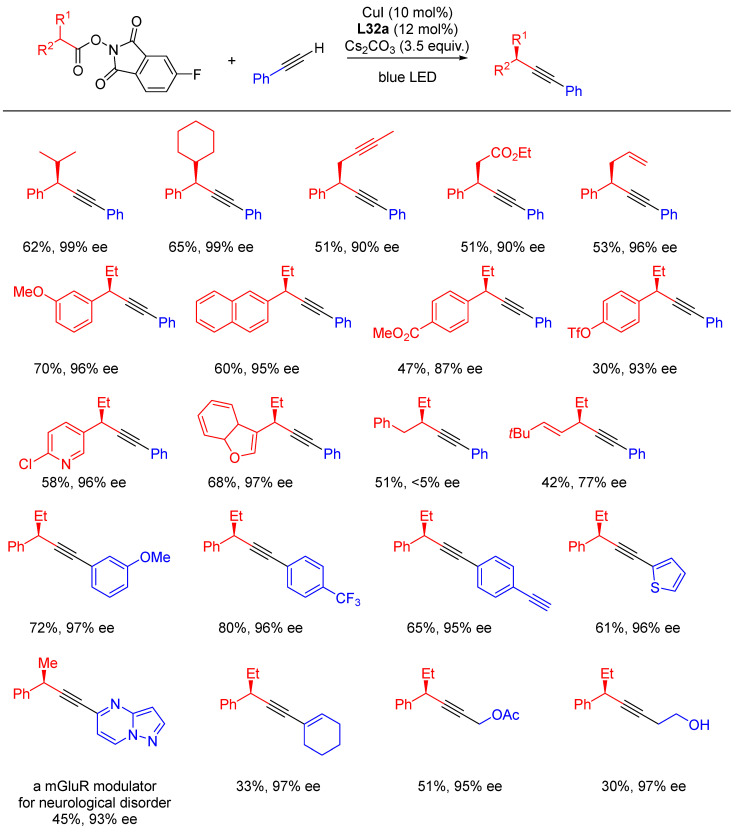
Decarboxylative alkynylation of *N*-hydroxyphthalimide esters.

The alkynylation of racemic secondary halides (see Scheme 38) has some major drawbacks as tedious synthesis and, often, low stability of the substrates occur. As a remedy, Liu’s group developed the method for the decarboxylative alkynylation of *N*-hydroxyphthalimide esters of racemic alkyl carboxylic acids [51]. The photoinduced radical reaction catalyzed again by **L32a**/copper complex proceeded with good yields and excellent enantioselectivities for a broad range of terminal alkynes and alkyl carboxylic acids (Scheme 40).

**Scheme 41:**
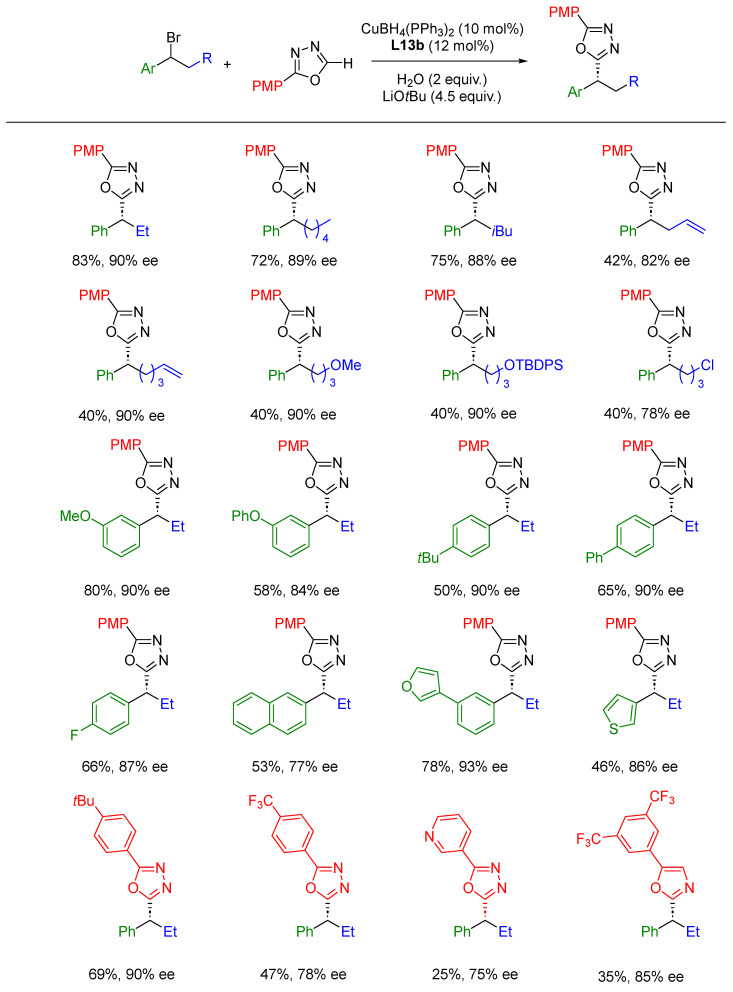
The enantioconvergent coupling of alkyl halides with azoles.

The catalytical system **L32b**/copper complex (modification of the phosphine moiety was required in order to achieve best results) was applied to the alkylation of azoles leading to pharmaceutically important α-chiral alkylated azoles [52]. The enantioconvergent coupling of alkyl halides with azole C(sp^2^)-H bonds proceeds under mild conditions yielding products with moderate-to-good yields and good-to-very-good enantioselectivities. This method not only provides an excellent complementary approach to the previous reported enantioselective heteroarene C(sp^2^)-H alkylation, but also an immediate access to enantioenriched α-chiral alkylated azoles for potential drug discovery (Scheme 41).

**Scheme 42:**
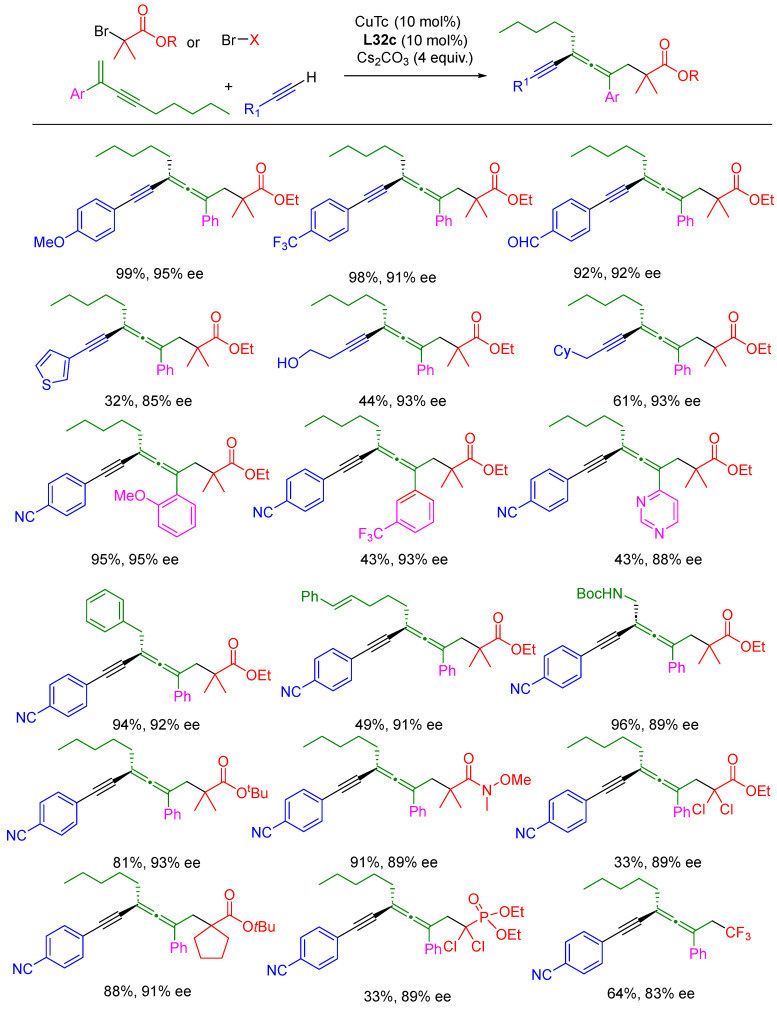
1,4-Carboalkynylation of 1,3-enynes.

On the basis of the previous results, Liu’s group proposed a similar strategy, which might also be suitable for enantiocontrol over the *sp*-hybridized allenyl radicals, thus allowing for the construction of tetrasubstituted chiral allenes [53]. They described the development of a copper-catalyzed three-component asymmetric radical 1,4-carboalkynylation of 1,3-enynes in the presence of the cinchona alkaloid-derived ligand **L32c**, providing straightforward access to diverse tetrasubstituted chiral allenes with moderate-to-good chemical yields and very good enantioselectivities (Scheme 42). 

**Scheme 43:**
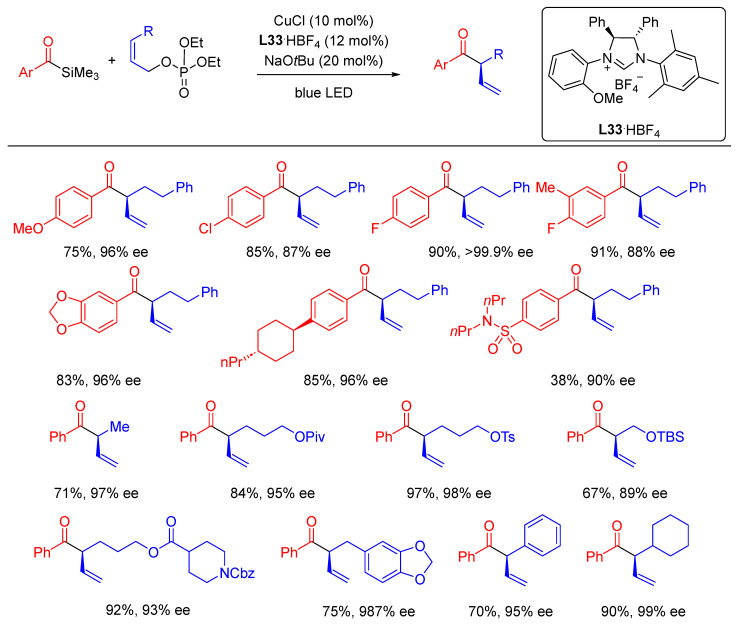
Umpolung acylation of primary allylic phosphates with acyl silanes.

Photoinduced transition metal catalysis differs from photoredox catalysis, in which metal plays a dual role as a photoabsorbent and a center for chemical bond cleavage, offering simplicity of reaction system and cost-effectiveness by avoiding the external photoredox catalyst. The mechanism consists of intermolecular photoinduced single-electron transfer (SET) between the catalytic metal complex and the substrate, followed by homolytic bond dissociation resulting in a highly reactive radical of substrate, which then recombines to the metal. Ueda et al. reported copper-catalyzed highly enantioselective umpolung acylation of γ-monosubstituted or γ,γ-disubstituted primary allylic phosphates with acyl silanes; the visible-light irradiation with a blue LED lamp led to the formation of α-branched β,γ-unsaturated ketones [54]. In the presence of chiral NHC ligand **L33**, the reaction proceeded with good yields and good-to-very-good enantioselectivities (Scheme 43).

### 2.2. Nickel-Catalyzed Enantioselective Radical Reactions

Chiral nickel complexes were recently found to be very useful tools in radical-based asymmetric synthesis. Again, most of the valuable results obtained in recent years were obtained in the presence of oxazolines or bisoxazolines as chiral ligands (vide infra). 

**Scheme 44:**
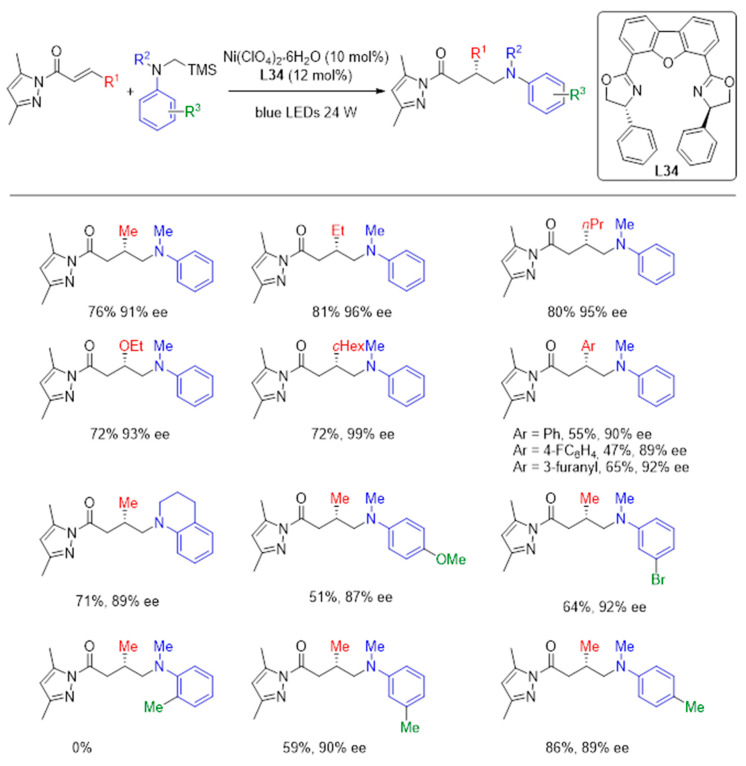
Conjugate addition of silylamines to α,β-unsaturated carbonyl compounds.

Shen et al. described an enantioselective photoredox reaction of α,β-unsaturated carbonyl compounds and tertiary or secondary α-silylamines. A complex of Ni(II) salt and box ligand **L34** (DBFOX) was used as catalyst, which in this process serves a double function—initiating SET process and providing the chiral environment for radical transformation [55]. The method allowed for the obtainment of chiral γ-amino carboxylic acid derivatives in good yields and good-to-excellent enantioselectivities up to 99% ee (Scheme 44).

**Figure 7:**
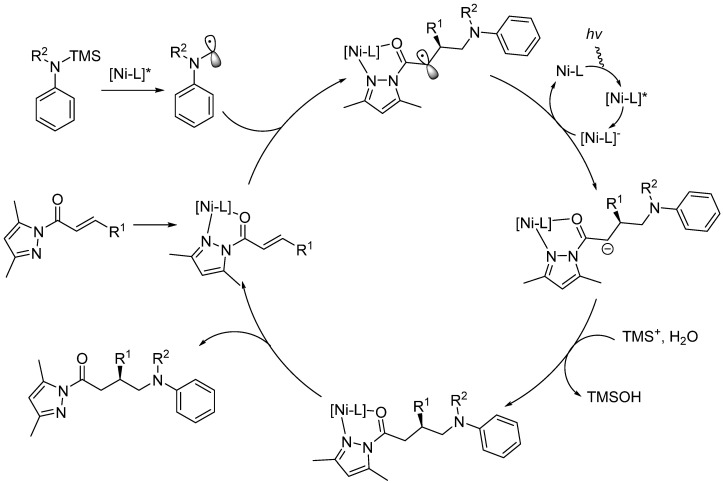
Plausible mechanism of conjugate addition of silylamines to α,β-unsaturated carbonyl compounds.

The authors found that the presence of the Ni(II) is crucial for the reaction. When Ni(COD)_2_, a Ni(0) complex, was used, the reaction failed to proceed. Also, the wavelength of light emitted by the LED diode was of great importance—no product was obtained, when red or yellow LEDs were used. On the basis of the literature, experimental data and mechanistic investigations with UV-Vis spectra and cyclic voltammograms, a plausible reaction mechanism was proposed (Figure 7).

**Scheme 45:**
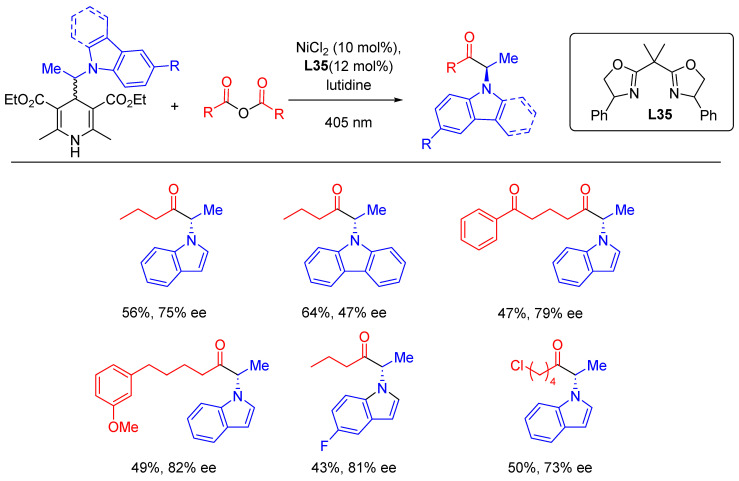
Synthesis of *N*-alkylated indoles.

**Scheme 46:**
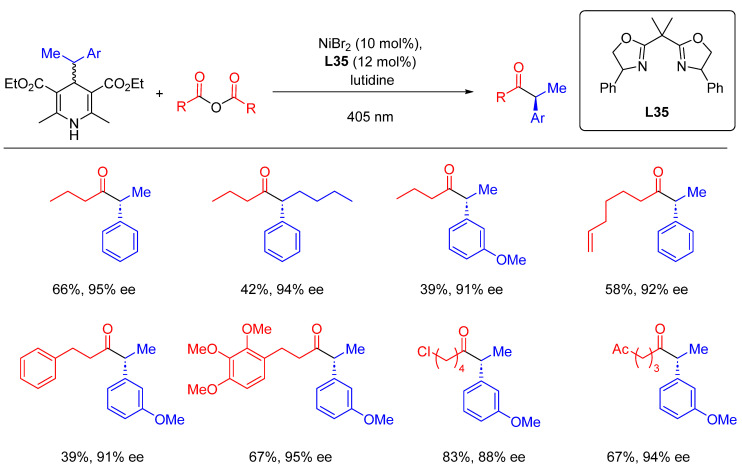
Acyl cross-coupling leading to α-aryl ketones.

Acyl cross-coupling was described by Melchiorre and co-workers [56]. The process is triggered by the excitation of 4-alkyl dihydropyridines (DHP) which hold a dual role of a radical source and reductant and therefore do not require an external photocatalyst. Symmetrical anhydrides were selected as electrophiles. Depending on the DHP used in this reaction *N*-alkylated indoles (Scheme 45) or *N*-aryl ketones could be obtained (Scheme 46).

The products were obtained with moderate-to-good chemical yields and enantioselectivities. Different substitution patterns for both DHPs as radical precursors and anhydrides as reagents were tolerated; however, only DHP bearing methyl substituent could be synthesized. 

The preparation of acyclic α,α-aryl, alkyl ketones required the synthesis of DHPs bearing both aryl and alkyl groups; also, another Ni catalyst was required—NiBr_2_ instead of NiCl_2_ in order to efficiently promote cross-coupling reaction. The reaction was compatible with various aryl rings substituted with electron-donating or electron-withdrawing groups, and was able to deliver products with good chemical yields and very-good-to-excellent enantiomeric excesses. 

**Scheme 47:**
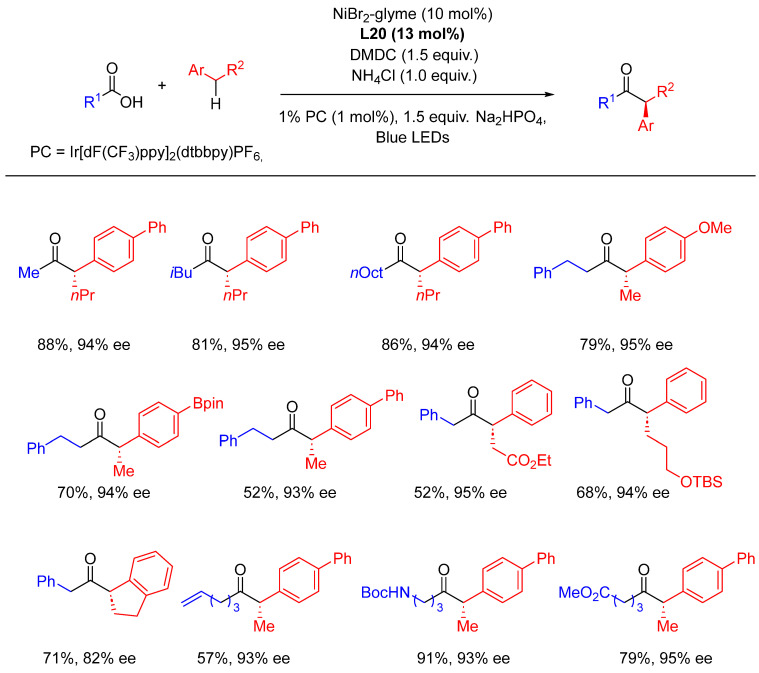
Synthesis of chiral α-aryl ketones.

Another approach to the synthesis of chiral α-aryl ketones was presented by Huan et al. [57]. Enantioselective benzylic acylation of alkylarenes was based on the in situ activation of carboxylic acids using dimethyl dicarbonate; a nickel and photoredox dual catalysis allowed for the synthesis of α-aryl ketones with good yields and very-good-to-excellent enantioselectivity (Scheme 47). The chiral environment was provided by bisoxazoline **L20**. This process exhibits high tolerance for functional groups and is applicable to broad range of carboxylic acids.

**Scheme 48:**
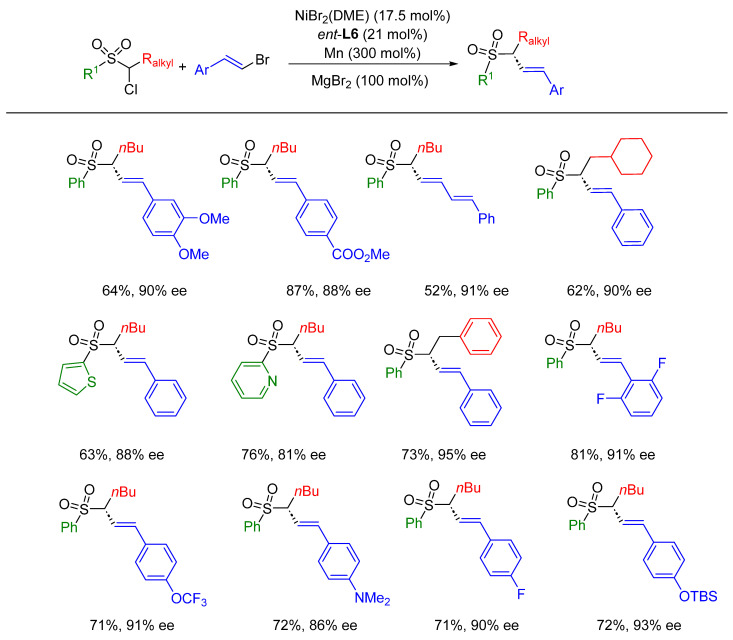
Synthesis of chiral allylic sulfones.

Geng et al. described the enantioselective synthesis of chiral allylic sulfones via Ni-catalyzed reductive cross-coupling of α-chlorosulfones with vinyl bromides [58]. The best enantioselectivity was obtained for box ligand *ent*-**L6**. The reaction outcome highly depends on the conditions and reagents used; replacement of the manganese with zinc, MgBr_2_ with TBAB or conducting the reaction at a higher temperature (10 °C) in each case led to substantially diminished ee’s. Reactions of the wide selection of β-aryl-substituted alkenyl bromides with electron-withdrawing and electron-donating groups at *para* positions resulted in the desired products with good yields and high enantioselectivities, up to 96% ee. Chlorosulfones with various α-alkyl, phenyl and alkyl silyl ether groups yielded products with very good enantiomeric excess, except pyridine and morpholine substituents, which failed to give ee’s better than 81% ee (Scheme 48).

**Scheme 49:**
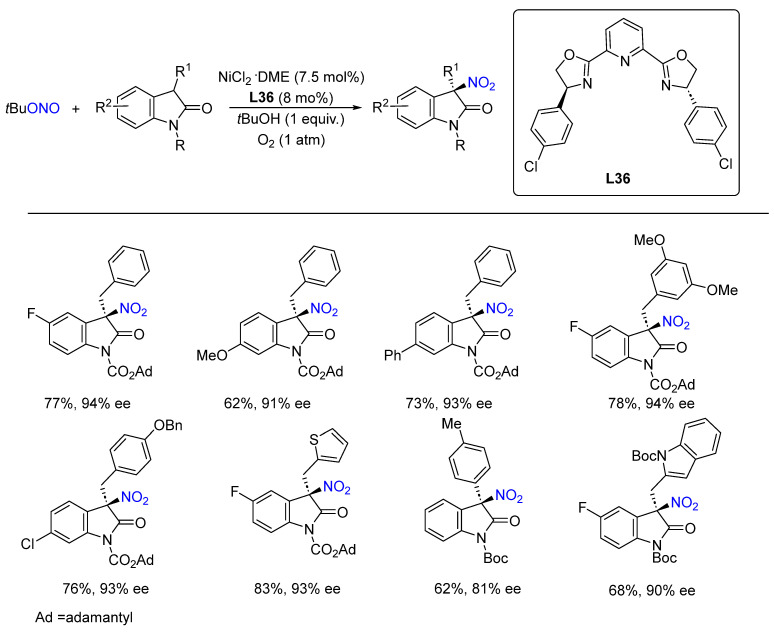
Nitration of oxindoles.

Lv and Li described the asymmetric radical nitration of oxindoles in the presence of pybox ligand **L36** [59]. Tertiary 3-nitrooxindoles were obtained with good yields and high enantioselectivities (Scheme 49). The reaction is quite insensitive to the electronic and steric effects.

**Figure 8:**
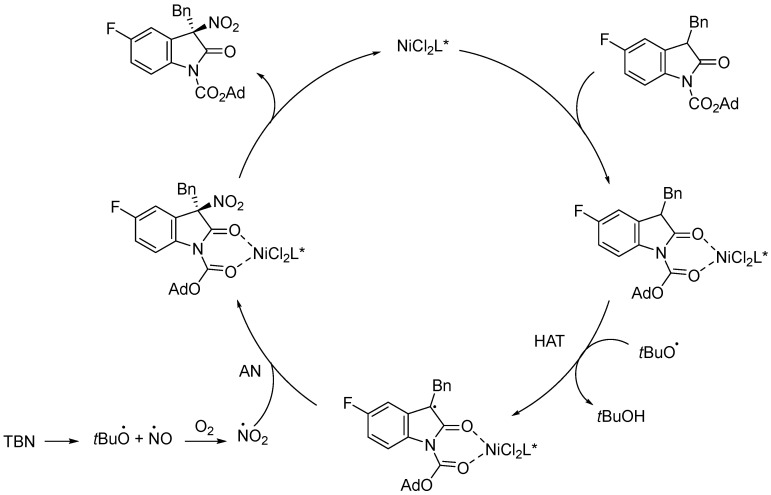
Mechanism of the nitration of oxindoles.

Experimental results supported with DFT calculations allowed for the proposal of a plausible mechanism involving single-electron-transfer (SET) as a process consisting of two stages: HAT initiated by *tert*-butoxyl radical and then asymmetric nitration where chirality is induced by nickel(II)/**L36** complex (Figure 8). 

**Scheme 50:**
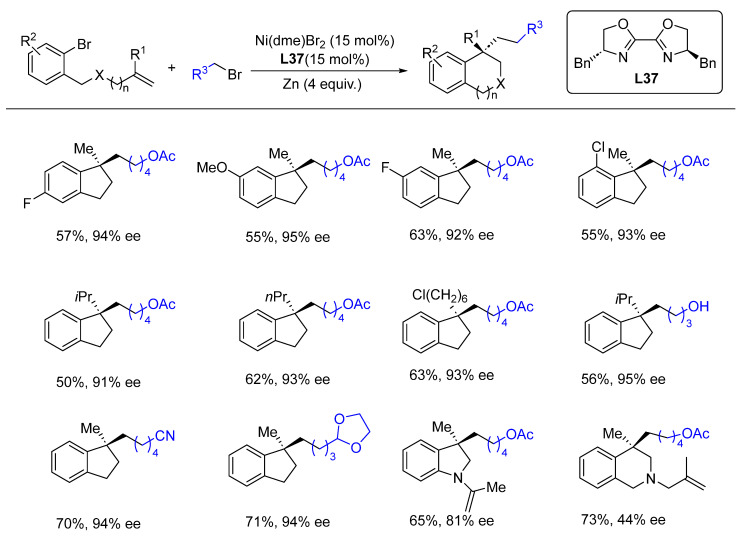
Reductive dicarbofunctionalization of unactivated alkenes.

Jin and Wang described the asymmetric reductive dicarbofunctionalization of unactivated alkenes via nickel-catalyzed reductive arylalkylation. Among the chiral ligands tested, only the less popular bi-oxazoline (biOx) ligand **L37** secured high enantiodiscrimination [60]. Benzene-fused cyclic compounds with quaternary stereocenters were obtained in this process (Scheme 50). Monosubstituted alkenes did not deliver the desired products due to a tendency to undergo β-hydride elimination. The formation of a five-membered ring is preferred and low enantioselectivity was observed for six-membered ring products. Indoles were obtained in high enantioselectivity. Various structurally complicated aliphatic primary bromides are compatible with this reaction; however, secondary and tertiary bromides failed to react.

**Scheme 51:**
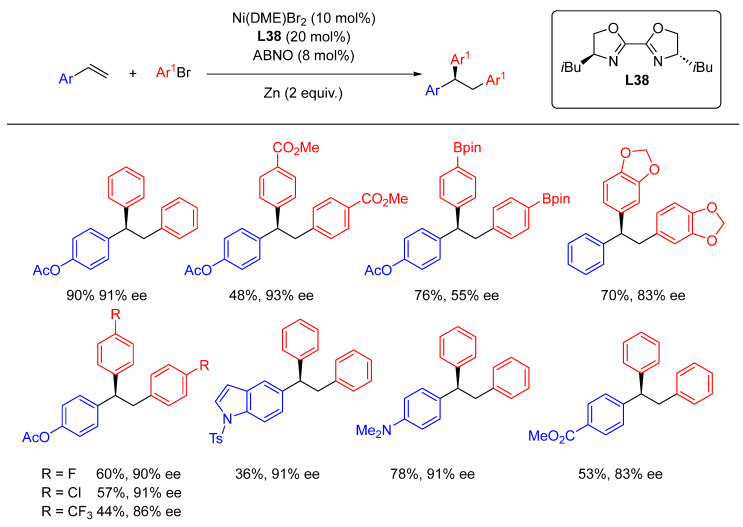
Diarylation of vinylarenes with aryl bromides.

Diarylation of vinylarenes with aryl bromides is another example where biOx ligand performed much better than classical box ligands [61]. Interestingly, not only did the chiral biOx ligand **L38**, but also *N*-oxyl radical additive 9-azabicyclo[3.3.1]nonane *N*-oxyl (ABNO) highly influenced enantiomeric excess in this reaction and, under optimized conditions, products were obtained with good yields and high enantioselectivity (Scheme 51).

**Scheme 52:**
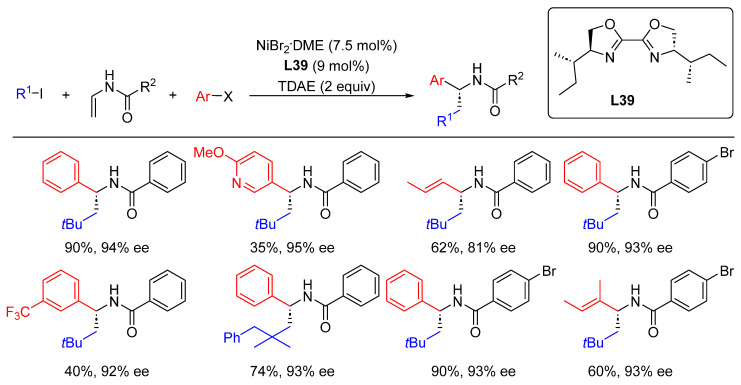
Dicarbofunctionalization of alkenes using alkyl halides.

The asymmetric dicarbofunctionalization of alkenes, a synthetically very useful process, has been reported by Wei et al. [62]. In the presence of nickel(II)/biOx complex, two different Csp^2^- and Csp^3^-halides add across the double bond of various vinyl amides with very good chemical yields and excellent enantioselectivities. The best results in this radical relayed reductive cross-coupling were obtained in the presence of (l)-(+)-isoleucine-derived bisoxazoline **L39** (Scheme 52). The broad spectrum of alkenes and halides can be used; however, no product was obtained for secondary alkyl iodides and *N*-Boc or *N*-Ts protected vinyl amides. 

**Scheme 53:**
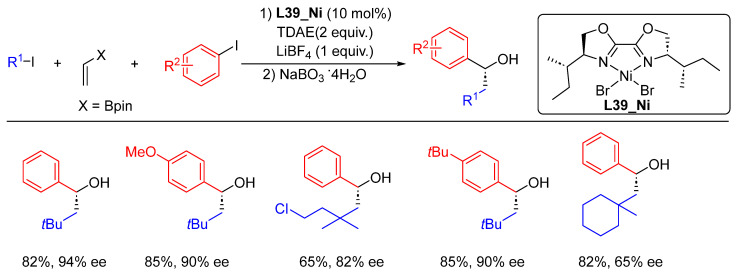
Dicarbofunctionalization of alkenes using vinyl boronic esters.

The scope of the olefinic substrate was extended to vinyl boronic esters; however, the modification of the procedure was required and preformed catalyst **L39_Ni**, instead of the one formed in situ, had to be used (Scheme 53). 

**Scheme 54:**
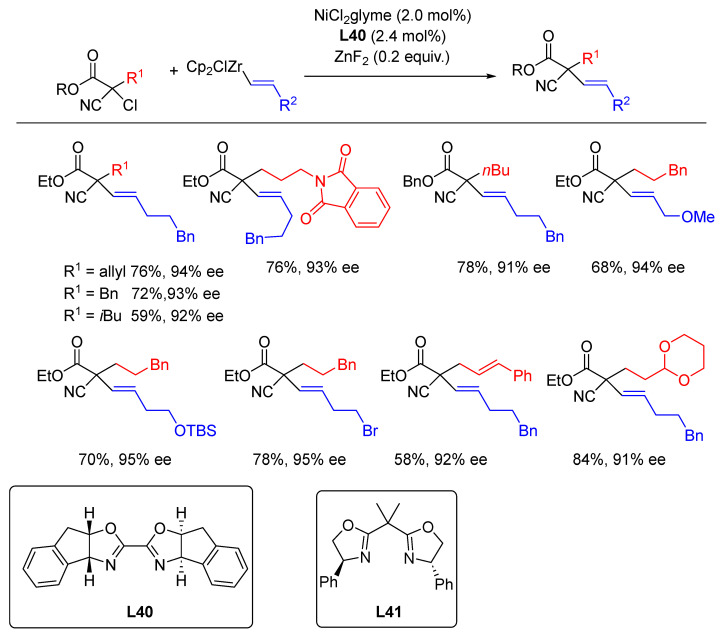
Alkenylation of acyclic tertiary alkyl halides.

As it was stated above, the structure of the ligand has to be complemental to the type of the reaction, reagents and the sterical requirements of the transition state. Very often popular, and in most cases effective, bisoxazoline ligands give inferior results. Fu and co-workers presented an enantioconvergent nucleophilic substitution reaction of tertiary alkyl halides with alkenyl zirconium reagents [63]. As substrates were used, there were two types of alkyl halides—acyclic and cyclic ones. The former required bioxazoline **L40** in order to achieve a high level of enantioselectivity; the other biOx (**L37**) or box ligand **L41** was much less selective or not selective at all (Scheme 54).

**Scheme 55:**
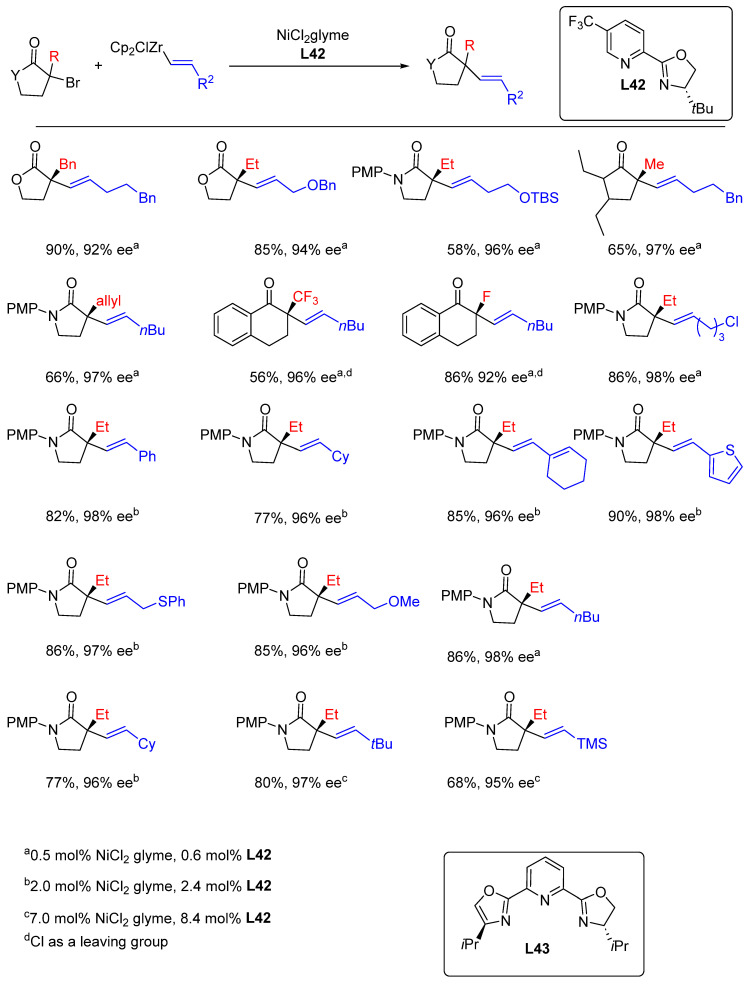
Alkenylation of cyclic tertiary alkyl halides.

The cyclic ones worked perfectly in the presence of pyOx **L42**, while the use of ligand **L40** gave very poor ee’s and pybox **L43** failed to promote the reaction (Scheme 55). 

**Scheme 56:**
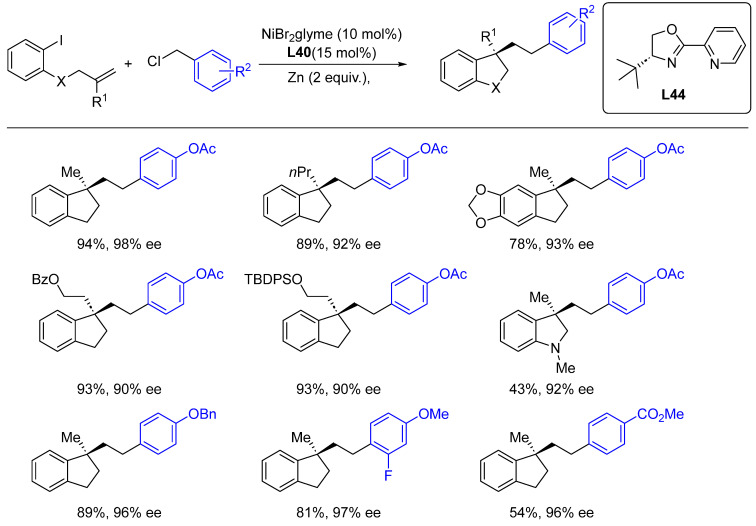
Arylbenzylation of unactivated alkenes.

Wang and co-workers extended their approach to the carbodifunctionalization of alkenes (see Scheme 50), to the arylbenzylation of unactivated alkenes with benzyl halides. The highest ee’s were obtained in the presences of the pyOx ligand **L44** [64]. The best results were achieved for benzyl chlorides and alkenes containing aryl iodide moiety, since the reactions of benzyl bromides with aryl bromides proceeded with diminished yields due to competitive homocoupling. A wide selection of benzyl and naphtyl chlorides successfully underwent the reaction with high enantioselectivities. Alkenes containing aryl rings with various electron properties were well tolerated. Indane, dihydrobenzofuran and indole frameworks were obtained in good chemical yields and high enantioselectivities (Scheme 56). 

**Scheme 57:**
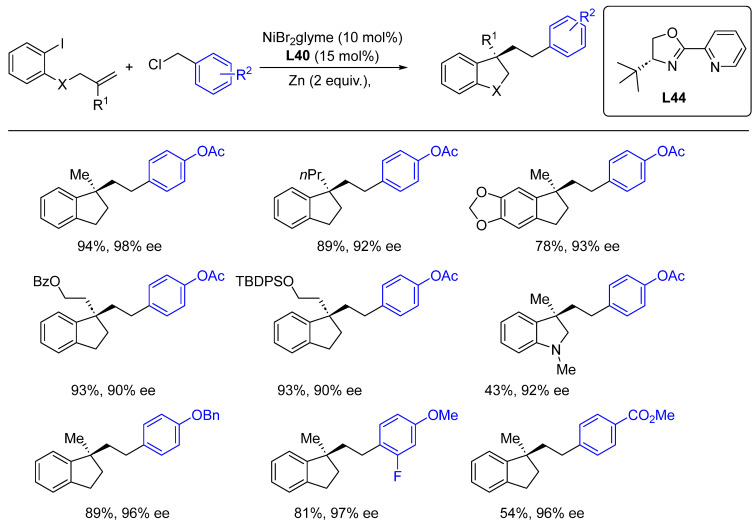
Crossover reaction of indanonecarboxamides with alkenes.

Zhang et al. presented an intermolecular radical–polar crossover reaction. Very high asymmetric induction was realized by the combination of *N,N*′-dioxide **L45**/Ni(II) complex in the presence of Ag_2_O as the best radical-generating oxidant. The counterion of nickel salt used in the process had significant impact on the stereocontrol; Ni(ClO_4_)_2_·H_2_O secured the highest enantioselectivities [65]. The reaction is applicable to various indanonecarboxamides, except acyclic β-ketoamides, and tolerant to the various substitution patterns of the aryl rings in both amide and alkene (Scheme 57).

### 2.3. Cobalt-Catalyzed Enantioselective Radical Reactions

**Figure 9:**
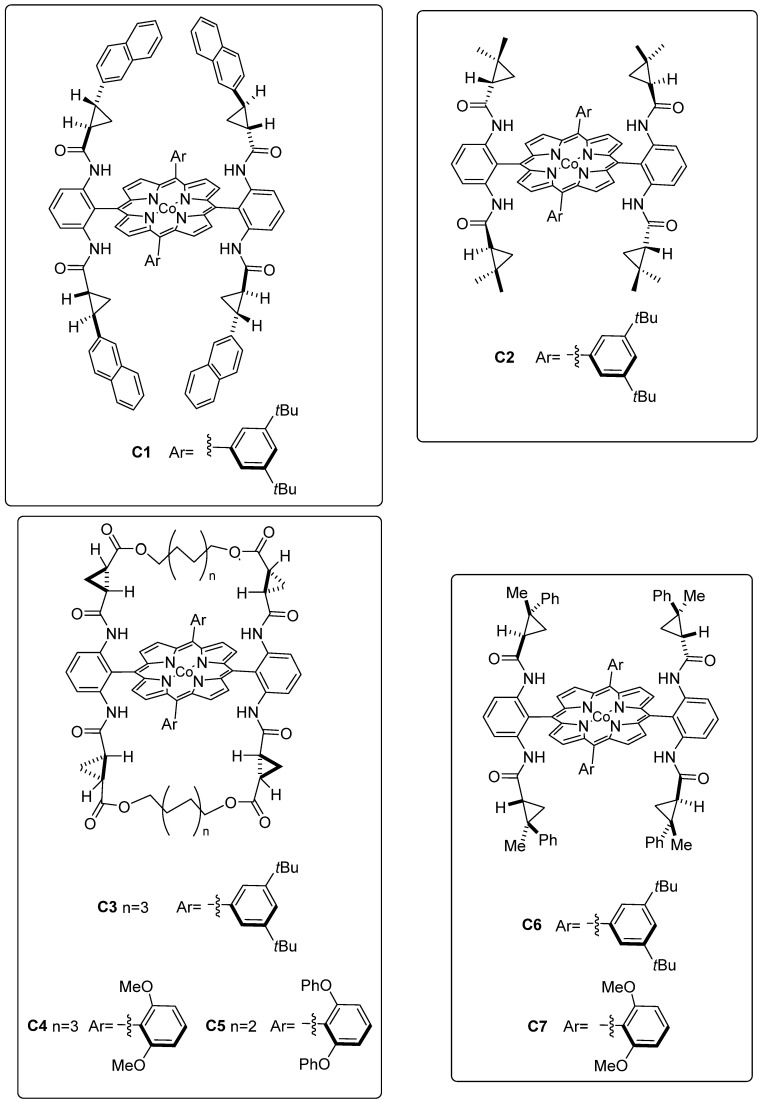
Co(II) porphyrin complexes.

**Scheme 58:**
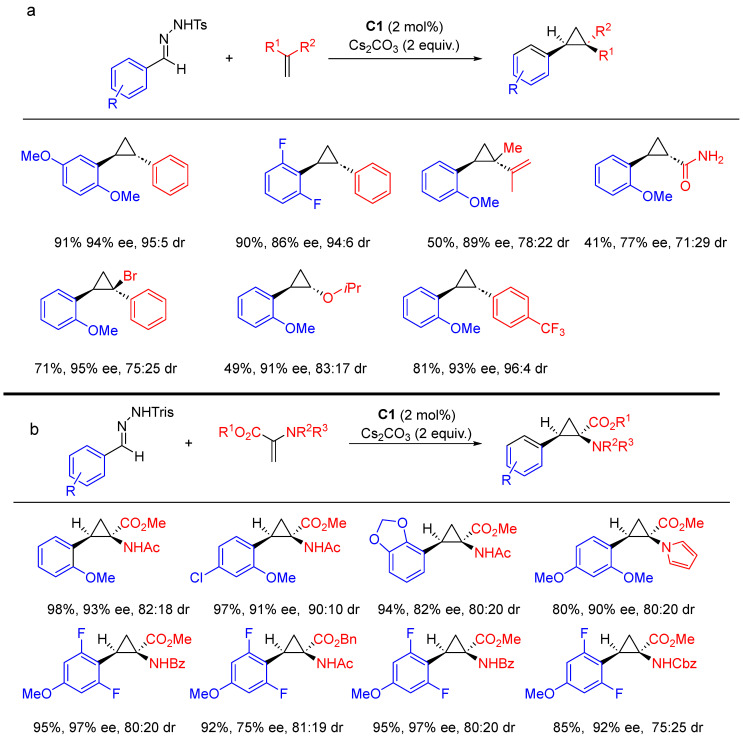
Cyclopropanation of 1,1-disubstituted alkenes.

Metal-based radical chemistry takes advantage of cobalt’s ability to form metal-centered radicals. Increasingly popular Co(II) porphyrin complexes with their ability to form stable metalloradicals have become excellent tools for asymmetric synthesis. Zhang developed a family of chiral, porphyrin-based cobalt complexes (Figure 9) and showed their great usefulness in asymmetric radical chemistry. He created several methods for the enantioselective cyclopropanation of alkenes by various diazo reagents in the presence of the above-mentioned complexes. The cobalt(II) complex **C1** catalyzes the reaction of the diazo compound generated in situ from *N*-arylsulfonyl hydrazones with 1,1-disubstituted alkenes to afford corresponding cyclopropanes with high chemical yields and high diastereo- and enantioselectivity, as presented in Scheme 58a [66]. This approach and the same catalyst **C1** were used for the cyclopropanation of dehydroaminocarboxylates using 2,4,6-triisopropylbenzenesulfonyl (trisyl) hydrazone, again with very good yields and high stereoselectivity (Scheme 58b) [67]. Some of the products were easily deprotected to give respective α-amino acids with very good yield and retained enantiopurity. 

**Scheme 59:**
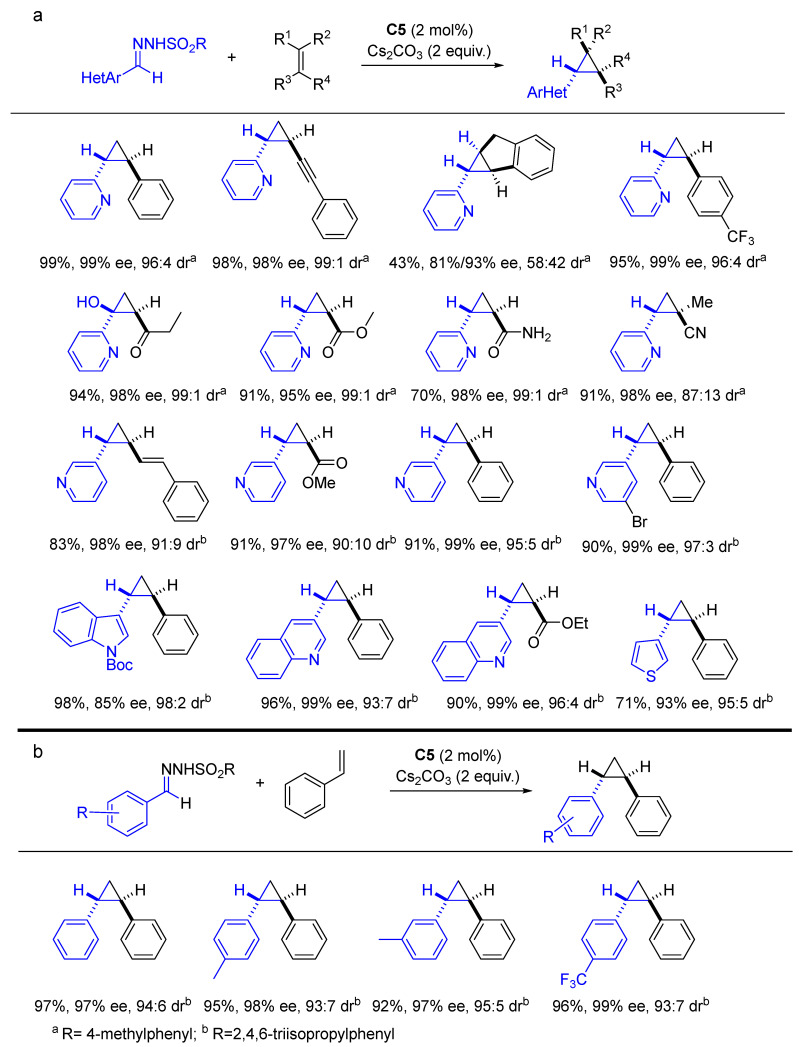
Synthesis of heteroaryl- and diarylsubstituted cyclopropanes.

This concept of the cyclopropane synthesis was further extended to heteroaryl-substituted cyclopropanes (Scheme 59a). In the presence of metalloradical complex **C5**, various heteroaryldiazomethanes cyclopropanated alkenes with excellent enantio- and diastereoselectivity up to 99% ee and 99:1 dr, respectively [68]. The methodology was also applicable to the asymmetric cyclopropanation of styrene, also with excellent selectivity (Scheme 59b).

**Scheme 60:**
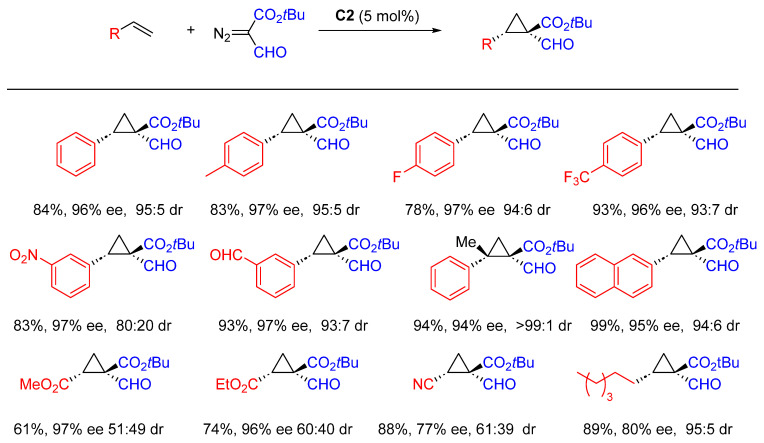
α-Formylodiazoacetates as cyclopropanation reagents.

Not only can *N*-sulfonylhydrazones be successfully applied in the cyclopropanation reactions; in the presence of cobalt(II) catalyst **C2,** α-formylodiazoacetates reacted with styrenes and acrylic acid derivatives. Excellent chemical yields, enantio- and diastereoselectivities were observed for styrene and its derivatives. Both electron-donating and electron-withdrawing substituents are acceptable; selectivity was only slightly influenced by the type of substituent. Formyl groups present in both olefin and diazoacetate remain intact in this process. Acrylic acid derivatives gave moderate-to-excellent ee’s, but the diastereoselectivity was low (Scheme 60).

**Scheme 61:**
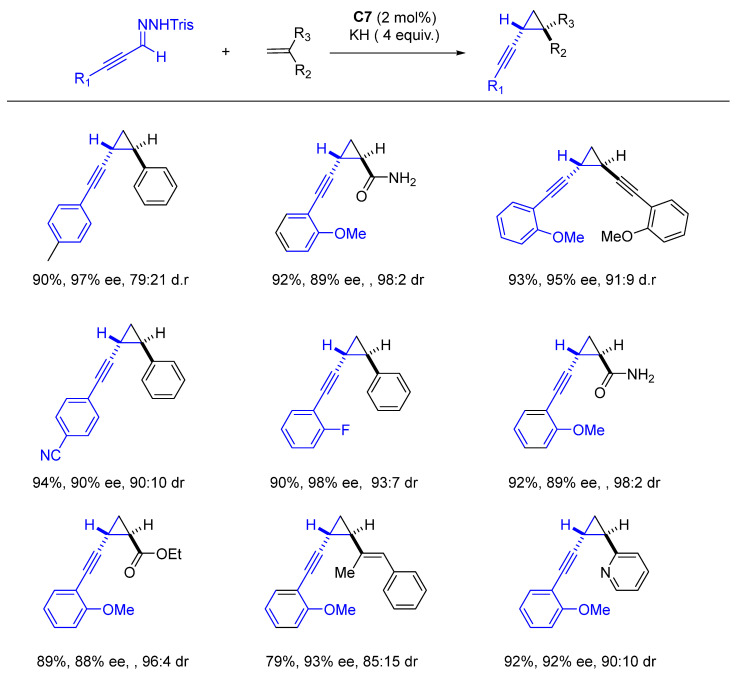
Synthesis of alkynyl-substituted cyclopropanes.

Another interesting cyclopropanation reaction was recently presented [69]. α-Alkynyldiazomethanes, formed in situ from the α-alkynylsulfonyl hydrazones, very efficiently reacted with various alkenes in the presence of **C7** as shown at Scheme 61.

**Scheme 62:**
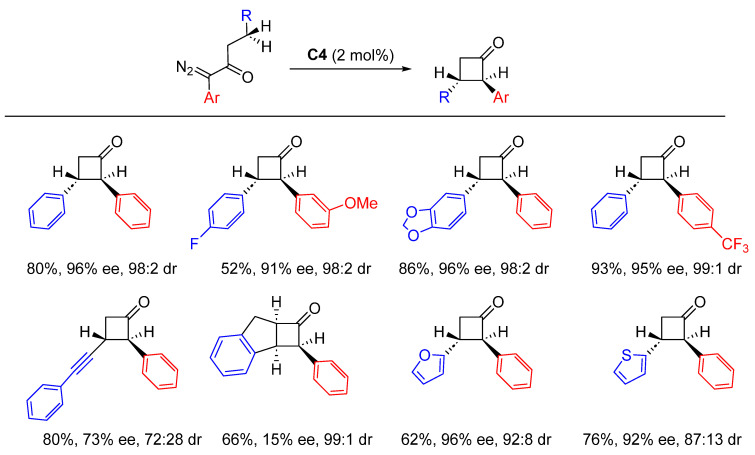
Radical ring-closure leading to cyclobutanones.

Co(II) porphyrin complexes were successfully used not only for the enantioselective synthesis of cyclopropanes, but also cyclobutene derivatives. Zhang developed a procedure for a radical ring closure, based on the 1,4-hydrogen atom abstraction (1,4-HAA), where the crucial role is played by the Co(II)-based metalloradical catalyst **C4** [70]. As shown in Scheme 62, various substituents were tolerated and cyclobutanones were obtained with usually good yields and excellent enantioselectivity. Interestingly, while diastereoselectivity was often moderate to good, the product underwent isomerization to the *trans* isomer; therefore, the dr for the final purified cyclobutanones was (with one exception of the phenylacetylene substituent) very high.

**Scheme 63:**
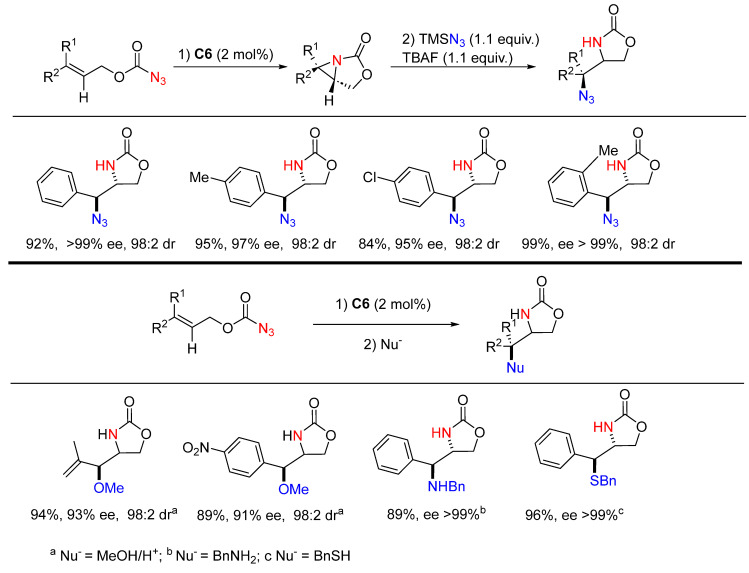
Bicyclization of allyl azidoformates as a tool for the synthesis of oxazolidinones.

The intramolecular, radical bicyclization of allyl azidoformates was performed using cobalt complex **C6** [71]. The reaction goes through the radical pathway without an additional base or oxidant. The reaction exhibits excellent enantio- and diastereoselectivity and several 3-oxa-1-azabicyclo[3.1.0]hexan-2-one compounds were obtained. However, these compounds are highly strained, making the isolation of pure compounds very difficult. Therefore, prior to isolation, aziridines were opened with nucleophiles yielding respective oxazolidinones; the original stereoselectivity was retained during this process (Scheme 63). 

**Scheme 64:**
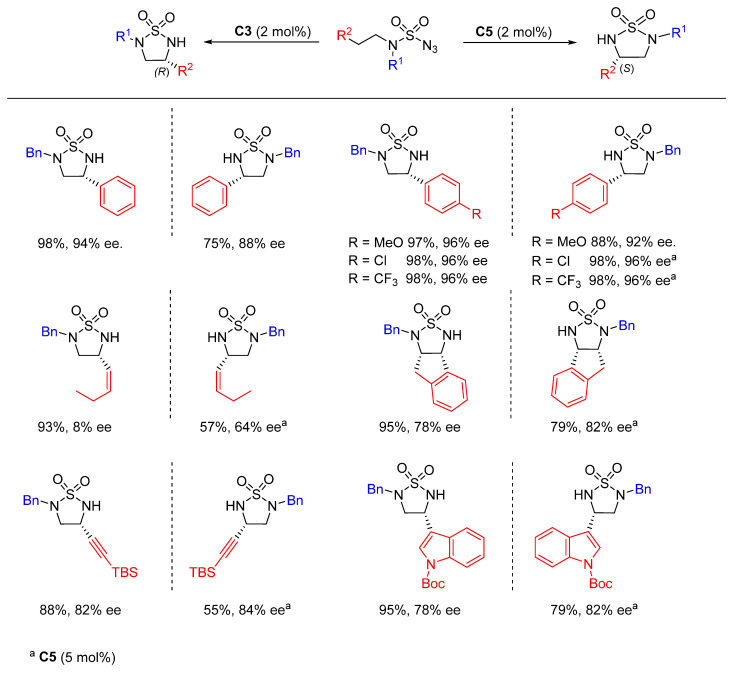
Intramolecular reactions of sulfamoyl azides.

A similar approach was applied for the intramolecular reactions of sulfamoyl azides [72]. Under Co(II)-based metalloradical catalysis, enantioselective radical 1,5-C-H amination proceeded with enantiodifferentiative hydrogen atom abstraction (HAA) and stereoretentive radical substitution. The most striking feature of this system is the enantiodivergent amination promoted by two very similar cobalt catalysts, **C3** and **C5**. These two complexes differ only by the length of the alkyl bridges (C_8_ vs. C_6_) and the type of the aryl substituent on the porphyrin ring, but the effect of these variations is tremendous. They behave as pseudo-enantiomers and lead to the opposite absolute configuration of the cyclic sulfamide’s stereogenic center with comparable, very high enantioselectivity (Scheme 64). In depth discussion of the stereochemical models, based on the kinetic isotope effect, DFT calculations and control experiments was presented by the authors. 

**Scheme 65:**
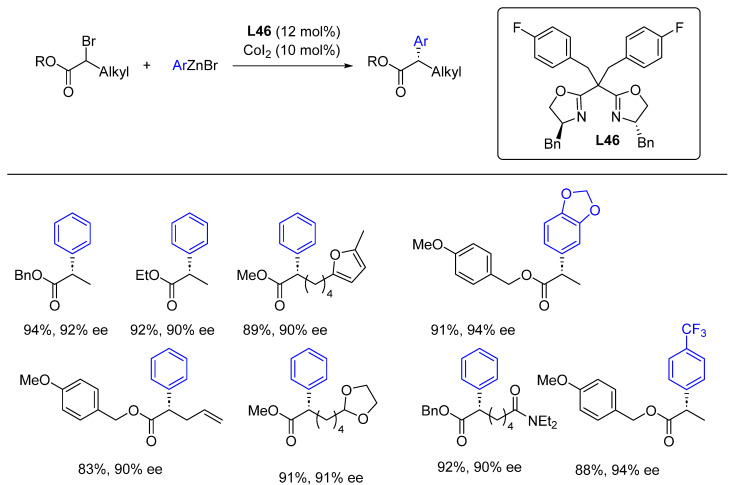
Negishi cross-coupling of arylzinc bromides with racemic α-halo esters.

The cobalt-catalyzed enantioselective Negishi cross-coupling of arylzinc bromides with racemic α-halo esters was reported [73]. The chiral environment was provided by box ligand **L46**, the best one among 14 box ligands tested. Several α-aryl alkanoic esters were obtained with high enantioselectivities and good chemical yields (Scheme 65). The mechanism of this supposedly radical process was not discussed.

**Figure 10:**
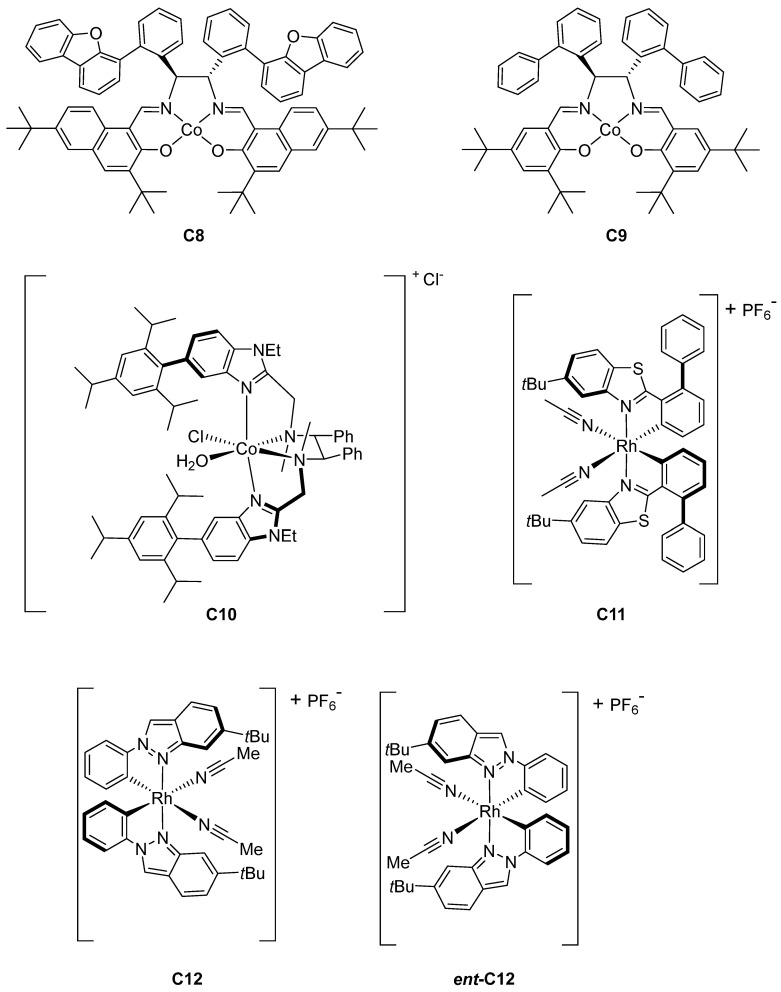
Cobalt and rhodium catalyst.

**Scheme 66:**
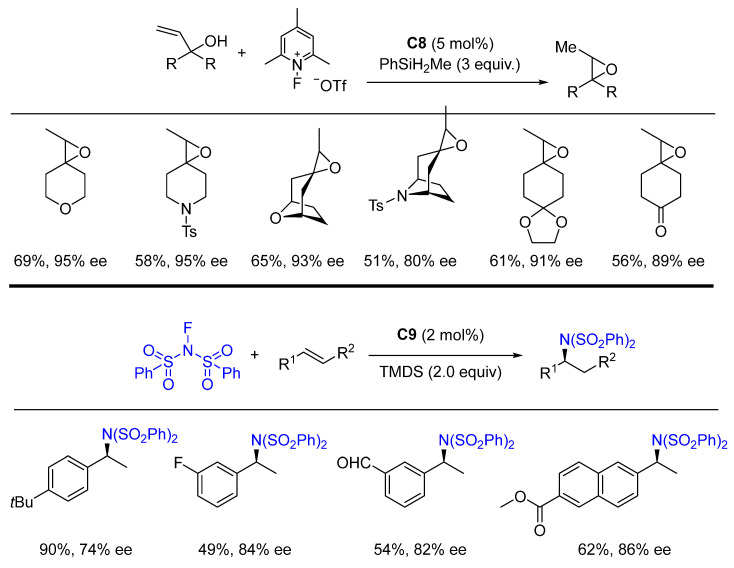
Use of Co-salen complexes for radical reactions.

Co-salen complexes are a very popular and efficient catalyst in enantioselective synthesis. They found use also in radical chemistry and salen complexes were used in intramolecular hydrofunctionalization of tertiary allylic alcohols (**C8**) [74], and hydroamination of alkenes (**C9**) [75], with moderate-to-high enantioselectivity (Figure 10, Scheme 66).

**Scheme 67:**
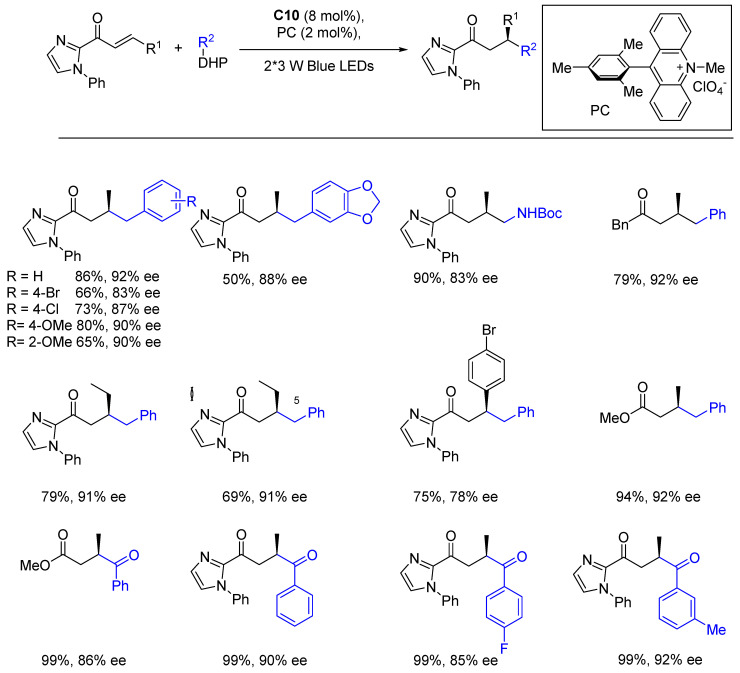
Alkylation and acylation of β-substituted enones.

The visible-light-induced conjugated addition of alkyl and acyl radicals to enones has been reported [76]. The reaction is promoted by the newly designed octahedral Co(II)-complex **C10**, whose structure was a modification of excellent Meggers’ catalysts [77]. The major difference is found in the metal-centered chirality in Meggers’ catalysts vs. carbon-derived stereogenic centers in catalyst **C10** (Figure 10). **C10** appeared to be an efficient catalyst in the visible-light-induced Giese reaction; addition of alkyl and acyl radicals to both aryl and alkyl β-substituted enones yielded desired chiral ketones with good enantioselectivity, up to 92% ee (Scheme 67).

### 2.4. Rhodium-Catalyzed Enantioselective Radical Reactions

**Scheme 68:**
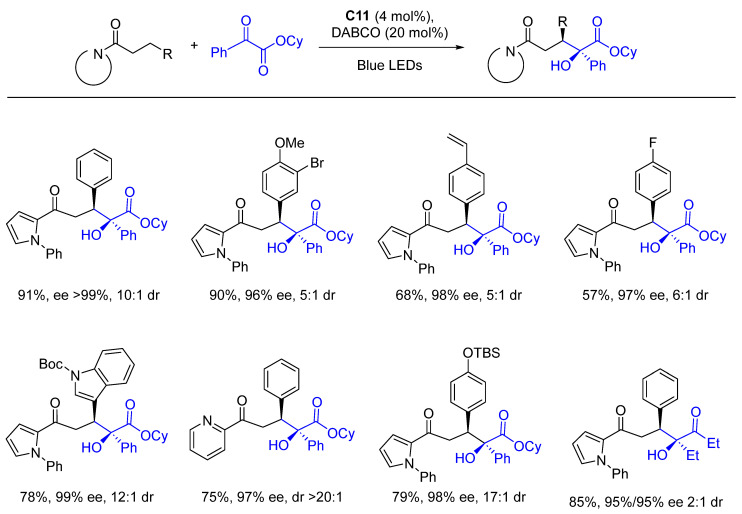
β-C(sp^3^)−H functionalization of 2-acyl imidazoles and 2-acylpyridines.

The chiral-at-ruthenium catalysts developed by Meggers have been applied to several processes. One of them is visible-light-activated asymmetric β-C(sp^3^)−H functionalization of 2-acyl imidazoles and 2-acylpyridines with 1,2-dicarbonyl compounds, mostly α-ketoesters [78]. The rhodium complex **C11** (Figure 10) plays a dual role of a chiral Lewis acid and a photocatalyst. The system exhibits good tolerance for various functional groups and reactions proceed with good yields and excellent stereoselectivity, even over 99% ee (Scheme 68).

**Figure 11:**
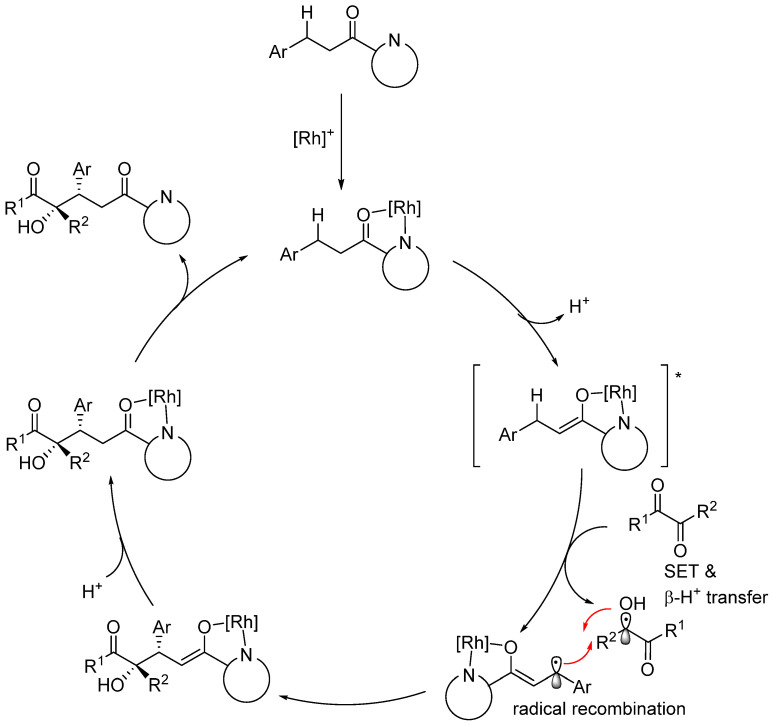
Mechanism proposed for β-C(sp^3^)−H functionalization.

On the basis of experiments and computational studies, a plausible mechanism was proposed (Figure 11).

**Scheme 69:**
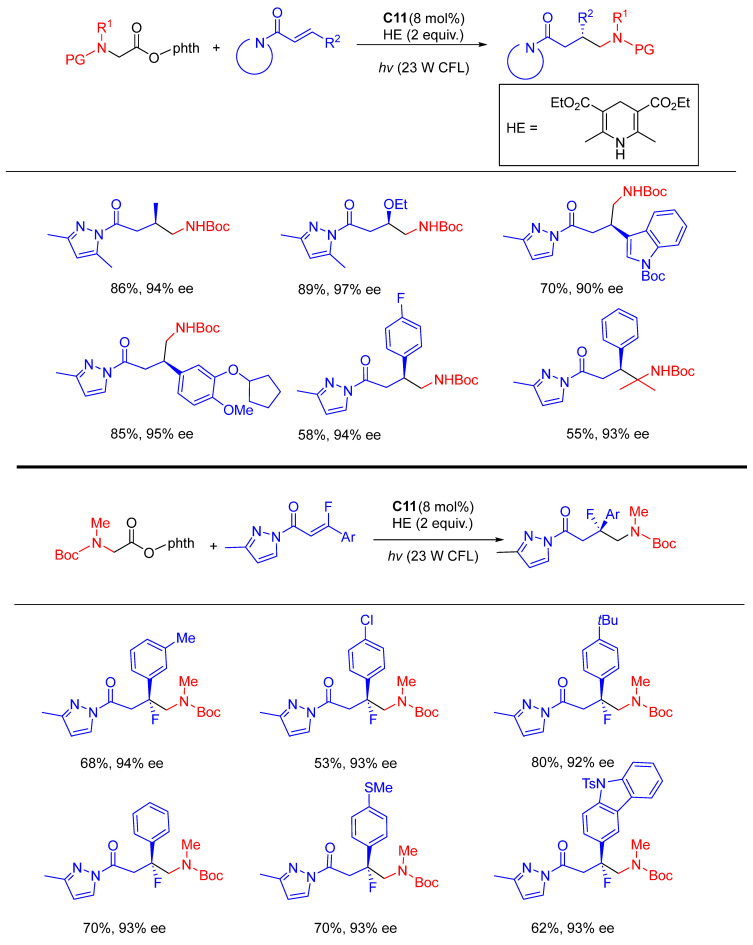
Synthesis of β-substituted γ-aminobutyric acid derivatives.

The same complex **C11** was applied to the enantioselective synthesis of β-substituted γ-aminobutyric acid derivatives [79]. The method makes use of glycine derivatives as the precursors to nucleophilic α-aminoalkyl radicals, which subsequently undergo enantioselective radical conjugated addition to α,β-unsaturated *N*-acylpyrazoles in the presence of Hantzsch ester and **C11**. Authors found that the reaction proceeds with better enantioselectivity in the absence of fac-[Ir(ppy)_3_], which was used at the beginning as a photocatalyst. Various β-substituted γ-aminobutyric acid derivatives were obtained in good yields (42–89%) and with excellent enantioselectivity (90–97% ee). Aryl, ether and alkyl substituents at the β-position of *N*-acylpyrazoles were tolerated. An addition to β-disubstituted β-methyl-β-phenyl alkene did not yield the expected product; however, replacement of the methyl group with a fluoro one yielded the desired product with high ee. This was the first example of a radical-conjugated addition leading to potentially useful GABA analogues bearing β-fluorinated quaternary stereogenic centers (Scheme 69).

The mechanism of the above-described reactions catalyzed by chiral-at-rhodium complexes and the origin of the asymmetric induction were analyzed using computational methods [80,81]. 

**Scheme 70:**
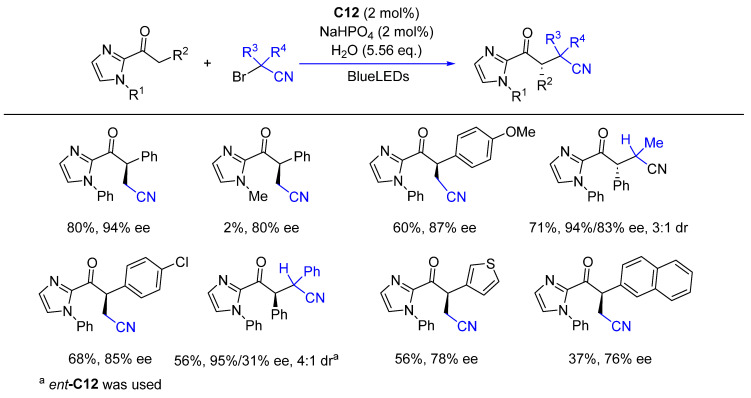
α-Cyanoalkylation of 2-acyl imidazoles.

The enantioselective α-cyanoalkylation of 2-acyl imidazoles was reported by Meggers and co-workers [82]. Substituted imidazoles were obtained with good yields and moderate-to-very-good enantioselectivities (Scheme 70) in the presence of the two new enantiomeric chiral-at-rhodium complexes **C12** and/or *ent*-**C12** (Figure 10). The new catalysts simultaneously act as a chiral Lewis acid securing asymmetric induction and as the photoredox catalyst for visible-light-induced redox processes. 

**Scheme 71:**
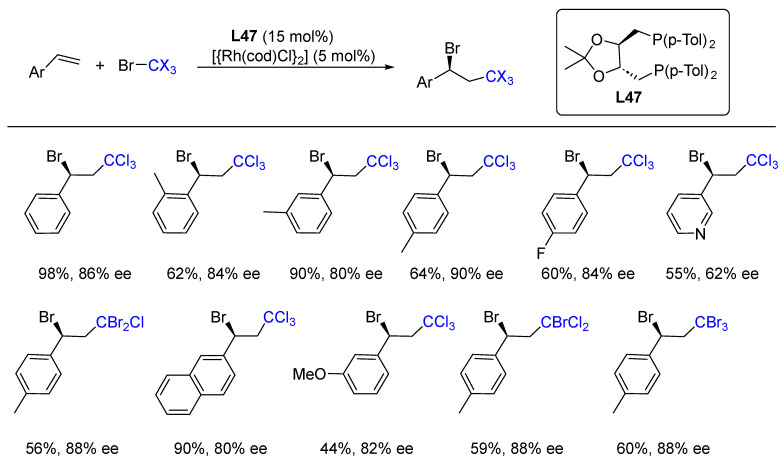
Enantioselective Kharasch reaction.

Chen et al. presented a more classical approach. The combination of rhodium complex [{Rh(cod)Cl}_2_] as a photocatalyst and *p*-Tol-DIOP **L47** as a chiral ligand allowed for the performance of an addition of CX_4_ to terminal olefins (Kharasch reaction) with very good enantioselectivity (Scheme 71). The authors performed a broad screening of the phosphine ligands in order to choose the most effective one [83].

### 2.5. Iridium-Catalyzed Enantioselective Radical Reactions

**Figure 12:**
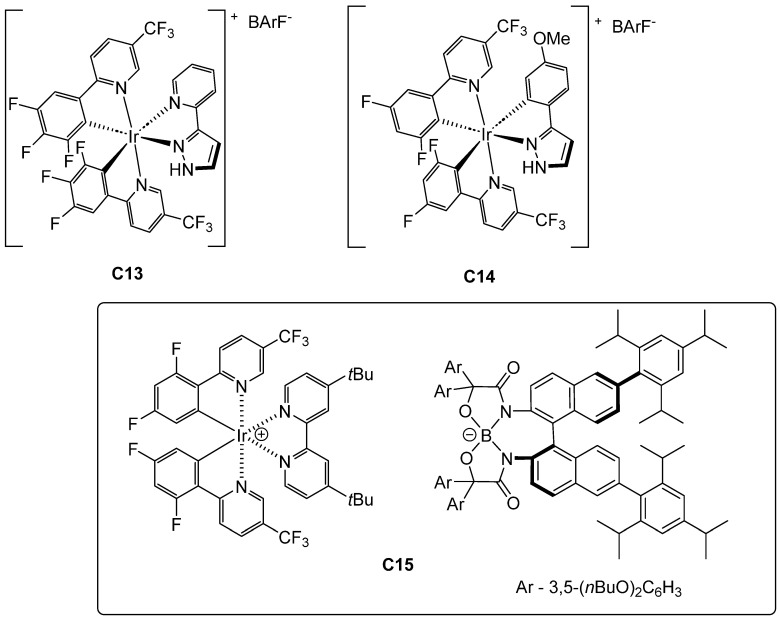
Chiral iridium complexes.

**Scheme 72:**
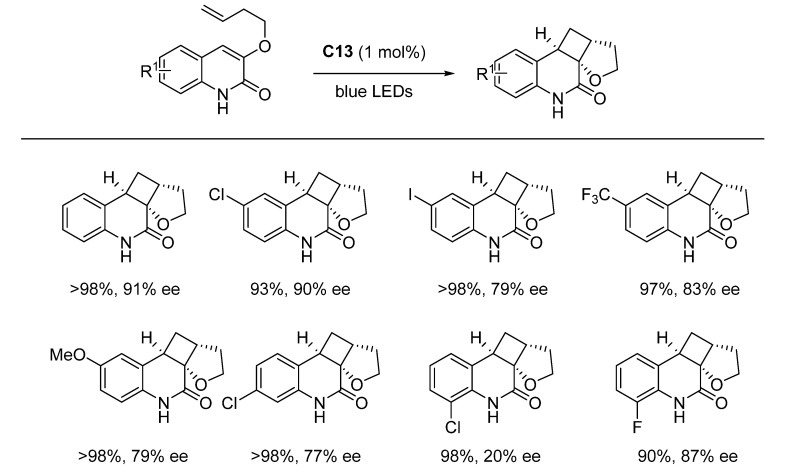
Intramolecular [2+2]photocycloadditions of quinolones.

Iridium complexes are well known photosensitizers. Chiral iridium complexes can provide a chiral environment enabling asymmetric induction and simultaneously serve as photosensitizers. Skubi et al. reported the synthesis and application of iridium complexes with the octahedral Ir(III) stereocenter [84]. The new catalyst **C13** (Figure 12) bearing a pyridylpyrazole hydrogen-bonding domain is an asymmetric triplet sensitizer and allows for the enantioselective intramolecular [2+2]photocycloadditions of quinolones with excellent yields and good enantiomeric excess (Scheme 72). The substitution of the alkene moiety was tolerated, but the enantioselectivity dropped significantly. The catalyst is very effective in amounts as small as 1 mol%.

**Scheme 73:**
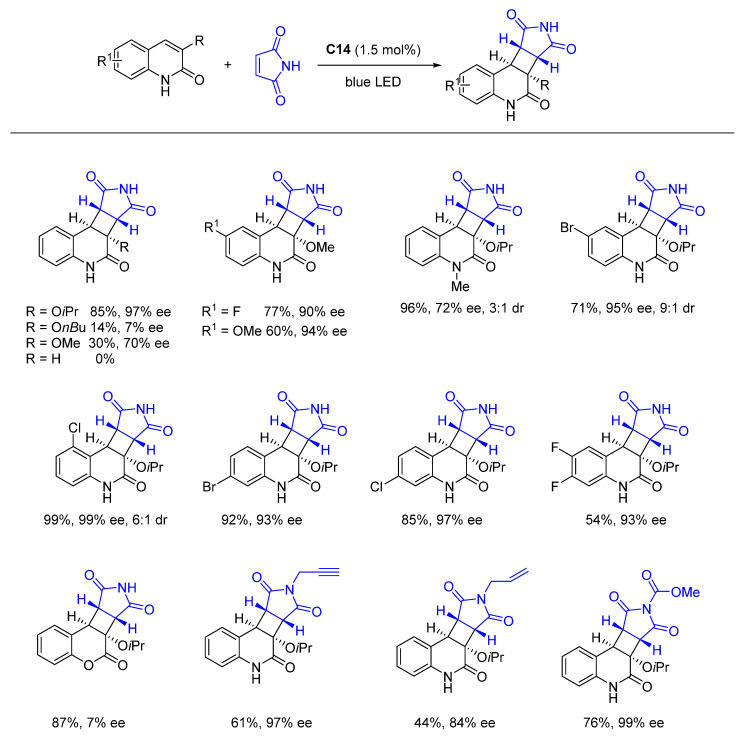
[2+2]Cycloaddition of 3-alkoxyquinolones.

This approach was extended to the intermolecular reaction. [2+2]Cycloaddition of 3-alkoxyquinolones catalyzed by chiral hydrogen-bonding iridium photosensitizer **C14**, a slightly modified structure of **C13**, is described [85]. The identity of 3-alkoxy substituent in the quinolone molecule highly influenced the yield and stereoselectivity of the reaction and its presence appeared to be indispensable (Scheme 73).

**Figure 13:**
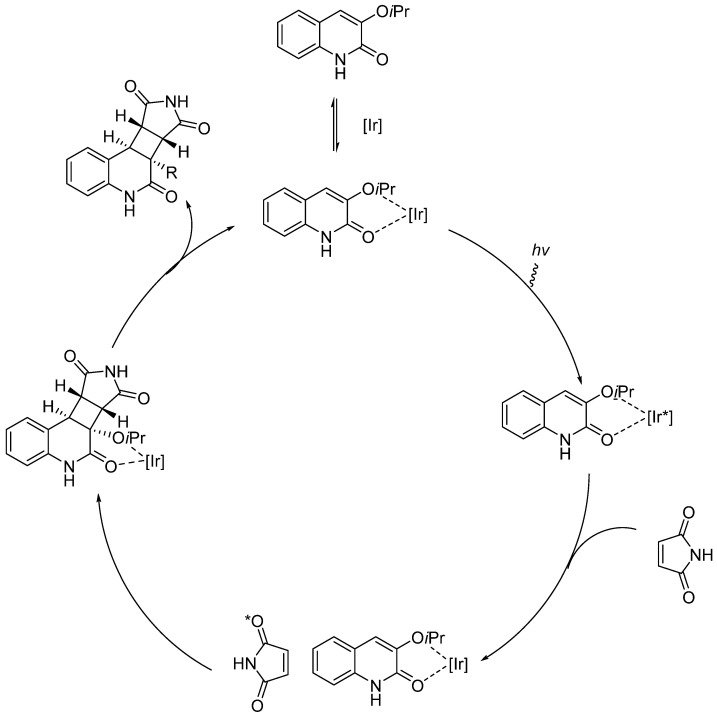
Plausible mechanism of [2+2]cycloaddition of 3-alkoxyquinolones.

Through a combination of synthetic, kinetic, spectroscopic, and computational studies, a mechanism of the process was proposed (Figure 13).

**Scheme 74:**
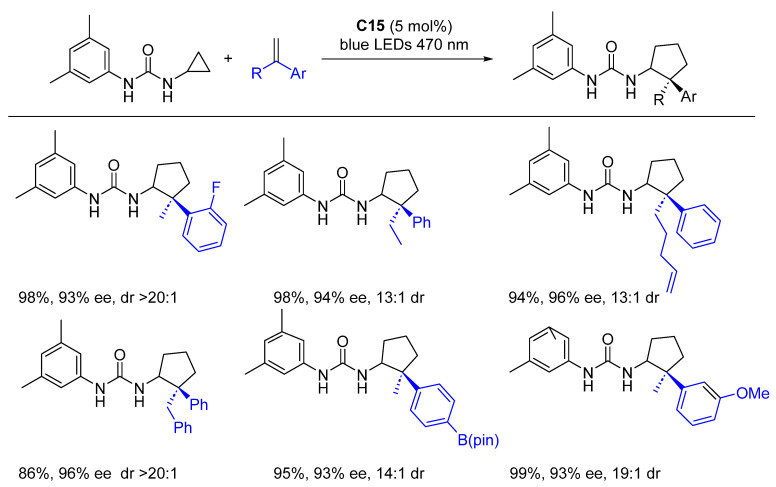
[3+2] cycloaddition of cyclopropylamine to α-alkyl-substituted styrenes.

The very interesting iridium photocatalyst was developed by Ooi and co-workers [86]. The racemic iridium complex [*rac*-Ir(dFCF_3_ppy)_2_(dtbbpy)] has been paired with a chiral borate anion to give catalyst **C15** (Figure 12). The chiral environment of the borate anion and not the stereochemistry of the iridium complex is responsible for the asymmetric induction. The control experiments showed that enantioselectivity in the presence of any of [Ir(dFCF_3_ppy)_2_(dtbbpy)]’s enantiomers was virtually identical. The urea moiety in the substrate was used as a directing group in the [3+2]cycloaddition of cyclopropylamine to α-alkyl-substituted styrenes and the products were obtained with excellent chemical yields and very-good-to-excellent enantio- and diastereoselectivity (Scheme 74).

**Scheme 75:**
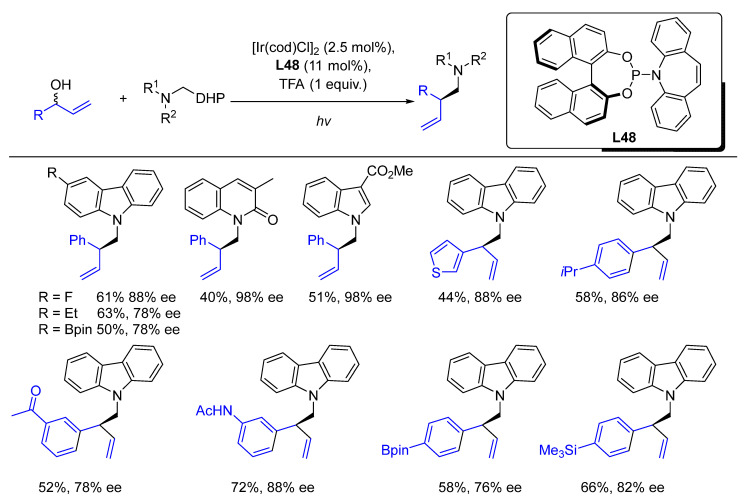
Alkyl–alkyl cross-coupling reactions of allylic alcohols.

The iridium complex [Ir(cod)Cl]_2_ combined with various phosphoramidite chiral ligands, e.g., **L48** have been widely used as very successful catalysts in asymmetric allylic substitution reactions. Melchiorre and co-workers presented completely new possibilities for this type of catalyst. Under the influence of visible light, the photoexcitation of the organometallic reagent can lead to a radical-based pathway. That allows for alkyl–alkyl cross-coupling reactions of allylic alcohols with radical precursors [87]. The reaction proceeds with moderate-to-good yields and good-to-excellent enantiomeric excess (Scheme 75).

### 2.6. Iron-Catalyzed Enantioselective Radical Reactions

**Scheme 76:**
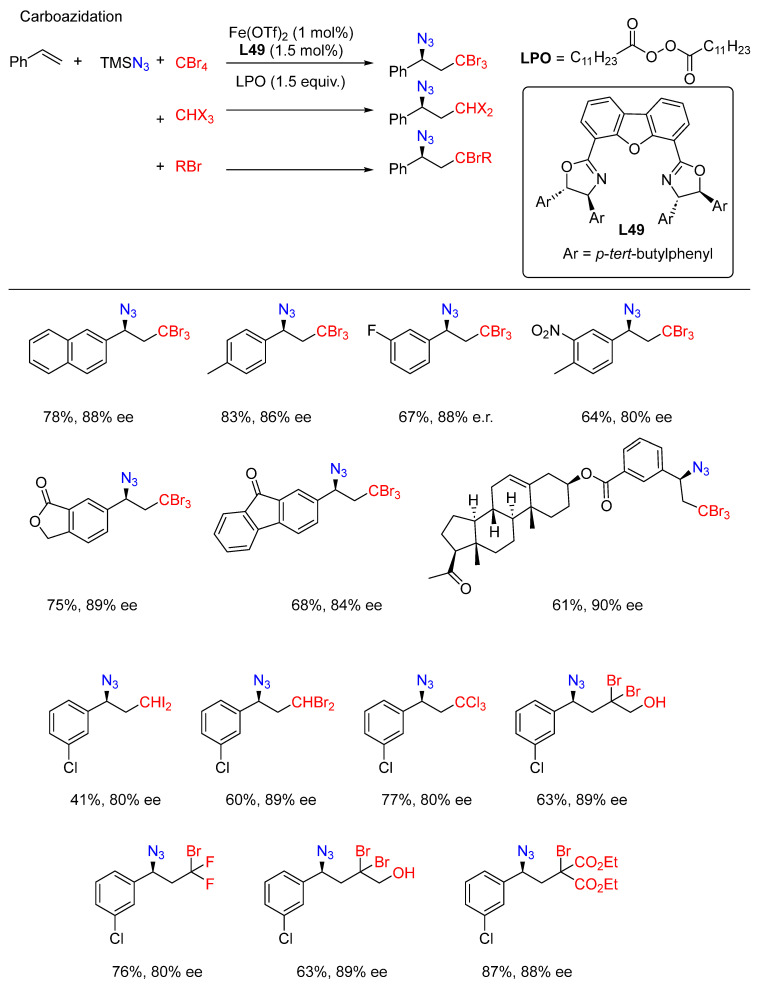
Carboazidation of styrenes.

**Scheme 77:**
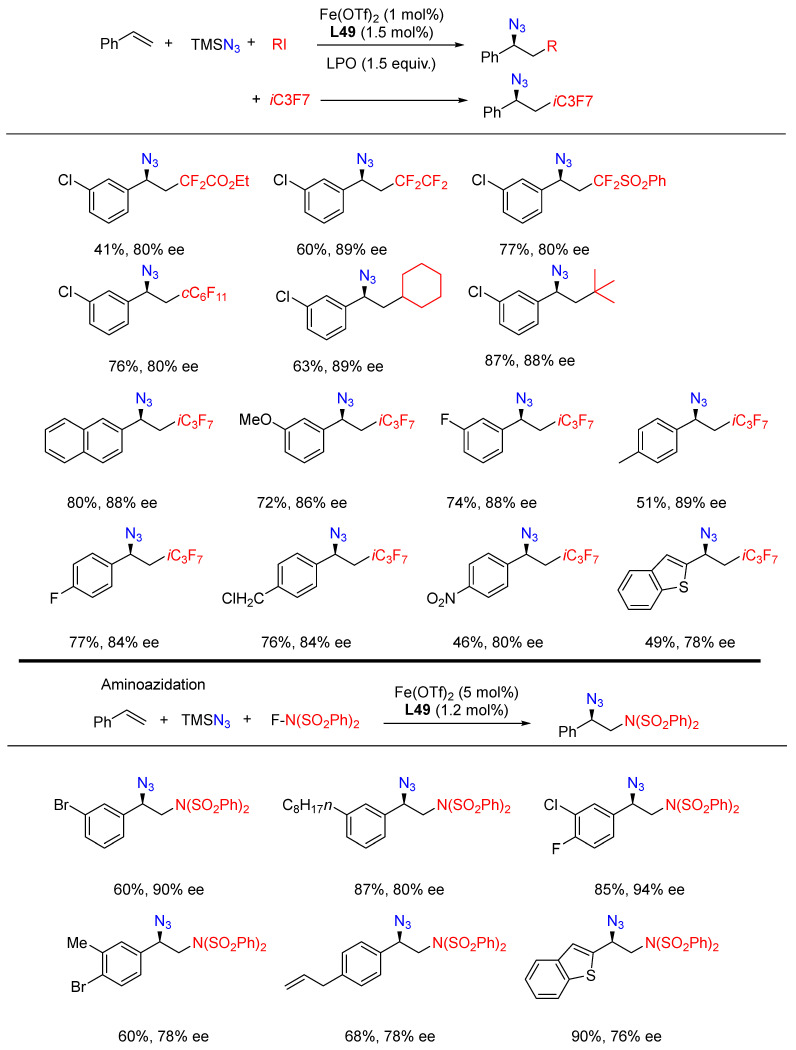
Carboazidation and aminoazidation of styrenes.

**Scheme 78:**
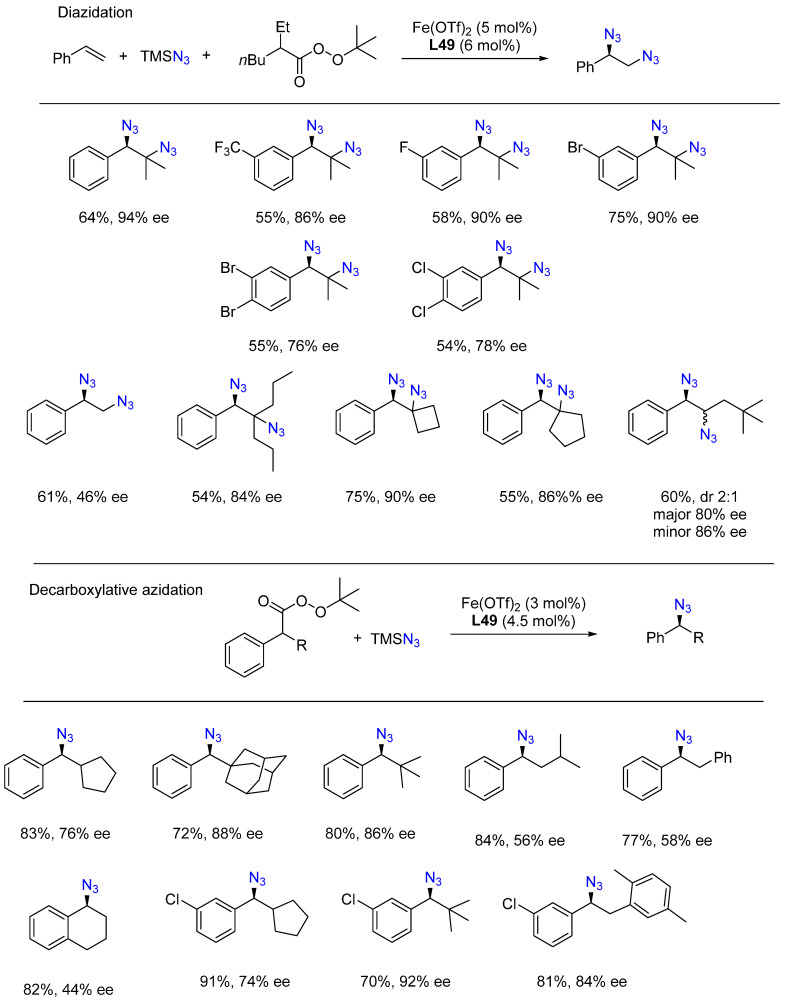
Diazidation and decarboxylative azidation.

Bao’s group published a series of papers presenting various approaches to the enantioselective radical azidation. In each case, the box complex Fe(II)-**L48** provided a chiral environment; the catalyst is very active, no more than 5 mol% was required for the completion of the reactions with good yields and enantioselectivities. The developed method was applied with success to the carboazidation of styrenes [88], aminoazidation and diazidation of styrenes [89] and decarboxylative azidation [90], as presented in Scheme 76, Scheme 77 and Scheme 78. 

### 2.7. Catalysis with Complexes of Other Metals

In the last 5 years, in addition to many papers devoted to catalysis with the metals described above, less popular metals have been used for catalysis in enantioselective radical reactions.

**Scheme 79:**
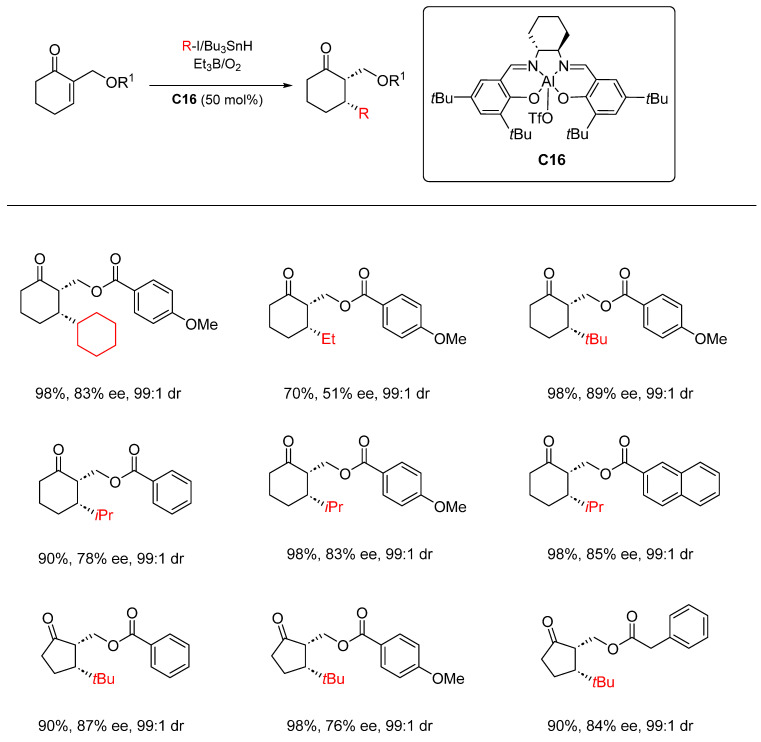
Conjugate addition to 2-hydroxymethyl-cycloalkenones.

Sibi and Nad reported enantioselective conjugate radical additions to 2-hydroxymethyl-cycloalkenones leading to 2,3-disubstituted cyclopentanones and cyclohexanones (Scheme 79) in high yield, good enantioselectivity and excellent diastereoselectivity [91]. The reaction was catalyzed by chiral salen-aluminum catalyst **C16**; other metals and ligands proved to be less effective. 

**Scheme 80:**
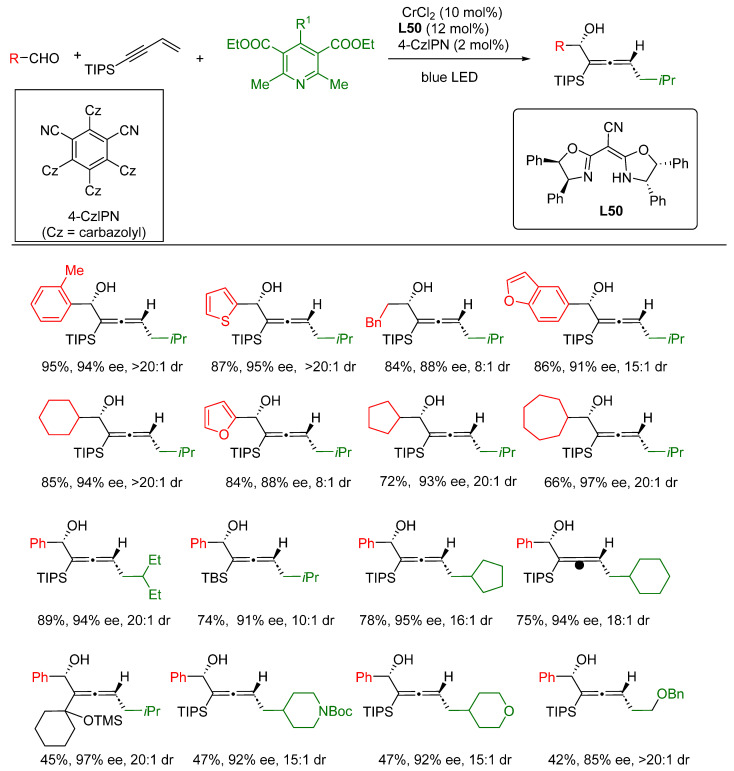
Three-component 1,4-dialkylation of 1,3-enynes.

Zhang et al. reported the asymmetric three-component radical 1,4-dialkylation of 1,3-enynes [92]. This photoredox reaction is catalyzed by photocatalyst 4-CzIPN and the chiral environment is provided by the chromium complex of the anionic box ligand **L50;** under mild conditions chiral allenols were obtained with very good yields and stereoselectivities (Scheme 80). 

**Scheme 81:**
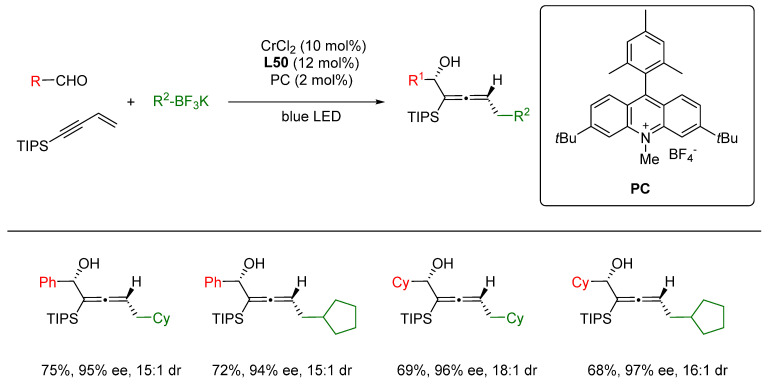
Modified three-component 1,4-dialkylation of 1,3-enynes.

The conditions depicted in Scheme 80 above were modified and Hantzsch ester, a radical precursor, and CzIPN, a photocatalyst, were replaced with alkyl tetrafluoroborates and acridine tetrafluoroborate, respectively, in order to increase the scope of the reagents (Scheme 81). 

**Figure 14:**
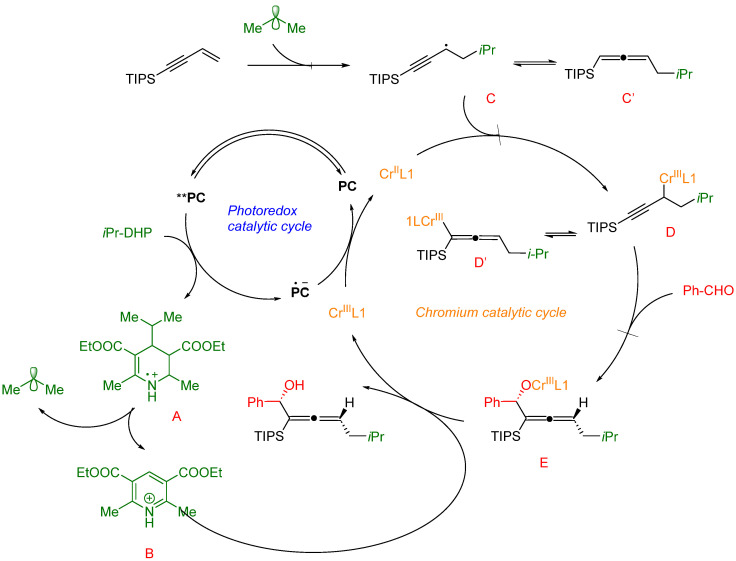
Plausible mechanism of 1,4-dialkylation.

The mechanism of the reaction presented in Scheme 80 was investigated using a radical trapping experiment, light on/off study and quantum yield measurement, as well as Stern–Volmer luminescence quenching studies. A direct correlation between photolysis and product formation indicates that the process undergoes a photoredox, instead of a radical-chain pathway as presented in Figure 14.

**Scheme 82:**
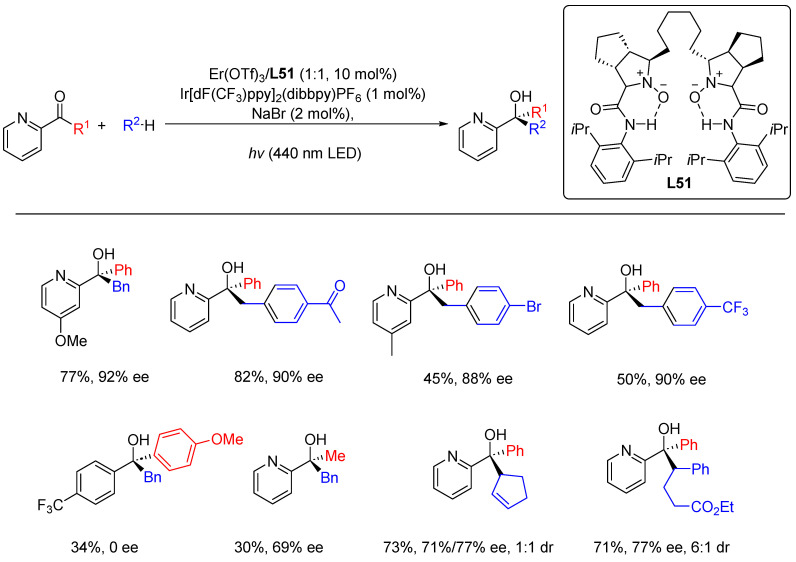
Alkylation of pyridyl aryl and alkyl ketones.

The enantioselective radical alkylation of pyridyl aryl and alkyl ketones was described by Yu et al. [93]. The asymmetric induction is secured by the erbium(III) complex of a very bulky dioxide, **L51**; as the photocatalyst that was used was iridium complex Ir[dF(CF_3_)ppy]_2_(dtbbpy)PF_6_, bromide-radical-mediated hydrogen atom transfer (HAT) is responsible for the formation of the alkyl radical. The series of tertiary alcohols was obtained with moderate-to-very-good chemical yields and enantioselectivities (Scheme 82). 

**Scheme 83:**
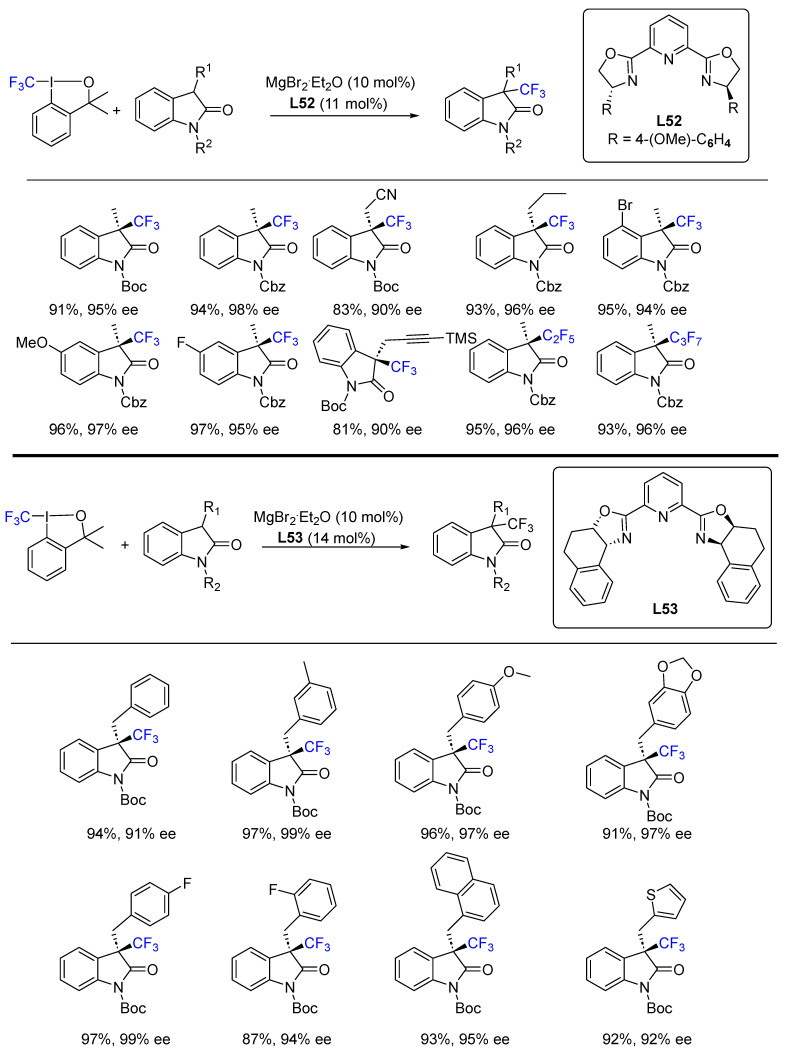
Trifluoromethylation of oxindoles.

Calvo et al. described radical asymmetric trifluoromethylation of oxindoles using hypervalent iodine-based reagent as a trifluoromethyl group source and a pybox–magnesium bromide complex as a chiral Lewis acid [94]. Neither pybox ligand provided high ee’s for all substrates; therefore, ligands **L52** and **L53** were used interchangeably and very-good-to-excellent yields and enantiomeric excesses were obtained (Scheme 83).

**Scheme 84:**
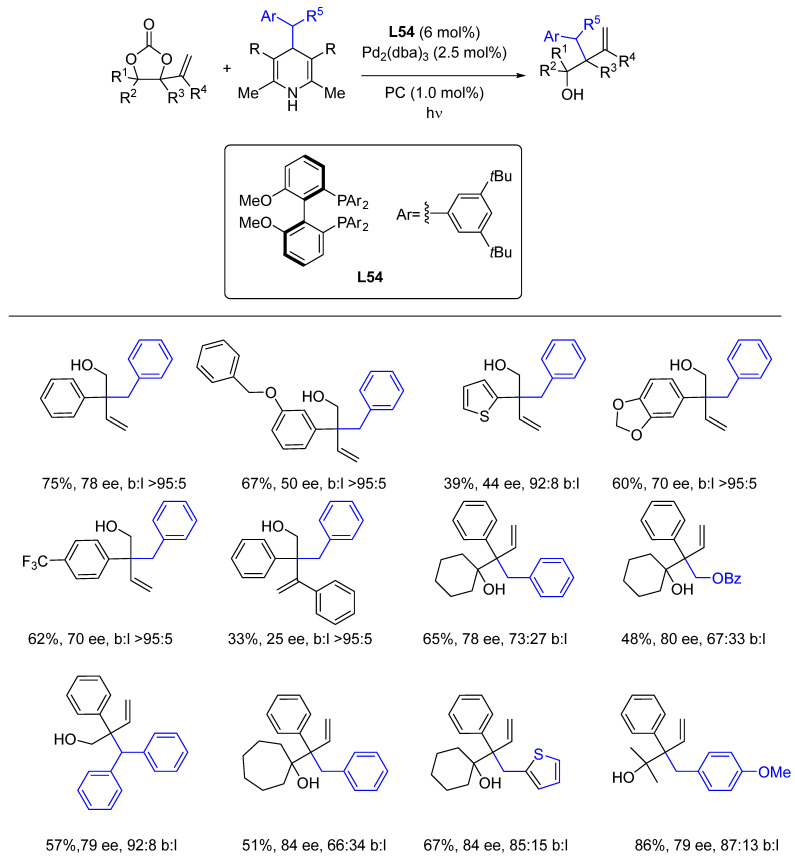
Synthesis of vicinal tetrasubstituted homoallylic alcohols.

Kleij and co-workers describe the method for the synthesis of vicinal tetrasubstituted homoallylic alcohols [95]. The procedure combines photoredox catalysis and palladium-catalyzed asymmetric allylic alkylation of vinyl cyclic carbonates, making use of Hantzsch esters as radical precursors, Ir(ppy)_2_(dtbbpy)PF_6_ (PC) as the photocatalyst and (*R*)-3,5-*t*Bu-MeOBIPHEP **L54** as the chiral ligand. Homoallylic alcohols were obtained with moderate-to-good chemical yields and enantioselectivities (Scheme 84). The ratio of the linear (addition to the terminal carbon of the double bond vs. branched (leading to the new stereogenic center) product was from excellent to moderate.

## 3. Dual Catalysis with Transition Metals and Organocatalysts

**Figure 15:**
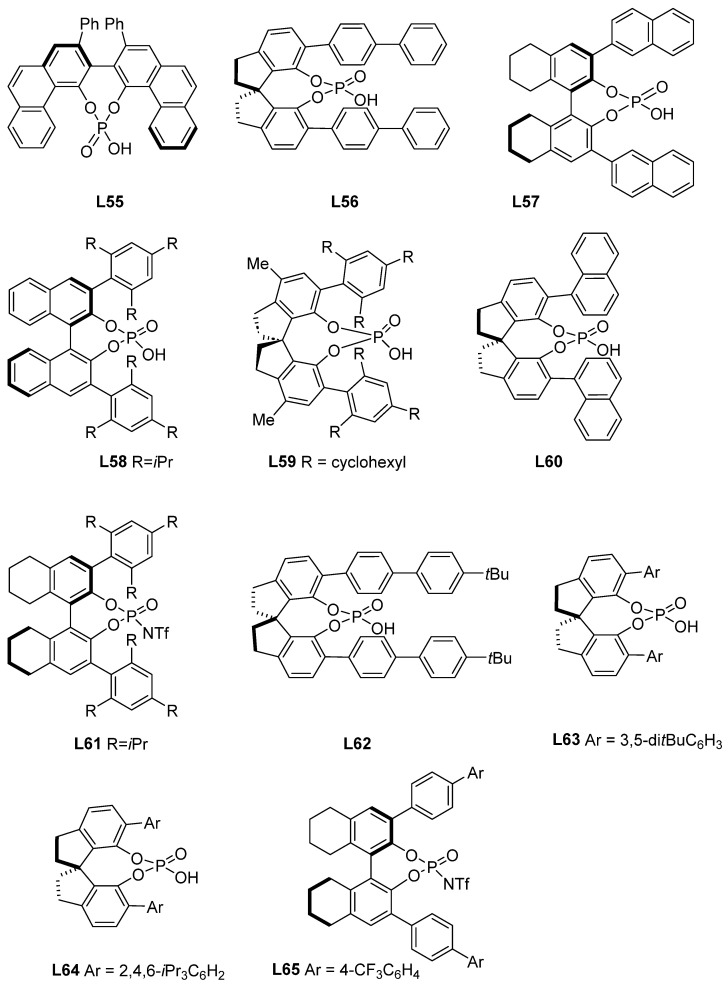
Chiral phosphoric acids used as catalysts.

In recent years, compounds developed earlier for the organocatalytic chemistry entered the field of radical chemistry as carriers of chirality. Several excellent papers depict the “purely” organic approach and will be presented in Section 4; herein, we discuss the border chemistry, where both transition metals and chiral organocatalysts are indispensable elements of the process. The organocatalysts applied in the dual-type catalysis are presented in Figure 15.

One of the hot topics is the enantioselective functionalization, including the difunctionalization, of alkenes.

**Scheme 85:**
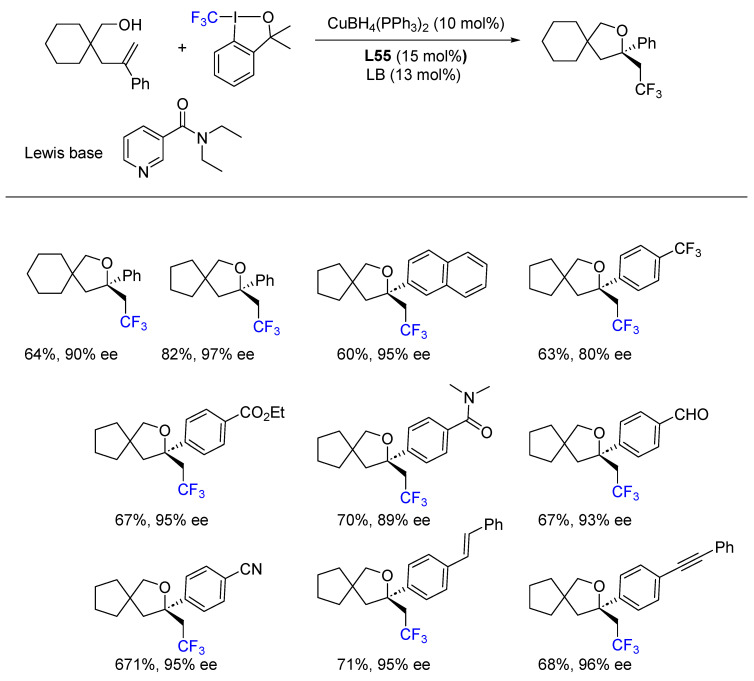
Oxytrifluoromethylation of alkenes.

Liu described several methods for the functionalization of styrenes. Enantioselective radical oxytrifluoromethylation of alkenes with the simultaneous cyclization was catalyzed by copper(I) complex, chiral phosphoric acid (CPA) **L55** with the assistance of *N,N*-diethylnicotinamide as an achiral Lewis base LB [96]. Chiral spirotetrahydrofurans bearing quaternary stereogenic centers were obtained with good chemical yields and very-good-to-excellent enantioselectivities (Scheme 85). Achiral pyridine, as shown in control experiments, acts as an ancillary copper ligand to greatly enhance the level of enantiocontrol. 

**Scheme 86:**
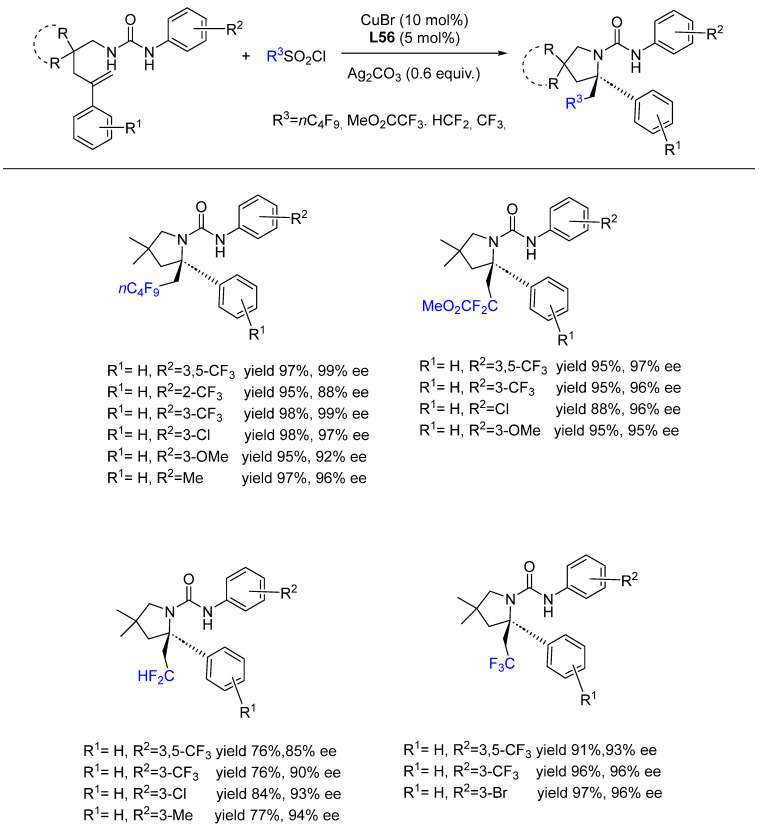
Synthesis of substituted pyrrolidines.

Liu and co-workers developed a methodology for the synthesis of substituted pyrrolidines bearing a quaternary stereogenic center by the functionalization of styrenes, α-substituted with various urea derivatives in the presence of chiral phosphoric acids [97]. The enantioselective aminoperfluoroalkylation and aminodifluoromethylation were catalyzed by CPA **L56**/Cu; the trifluoromethyl group was introduced using the inexpensive and easily available trifluoromethylsulfonyl chloride, and not by the usually used Togni’s reagent. Pyrrolidines were obtained with very good yields and excellent enantiomeric excesses (Scheme 86). The reaction is catalyzed by the chiral Brønsted acid and another one, HCl, is formed in situ. In an elegant way, the background reactions catalyzed by HCl are suppressed by an additive, a silver carbonate.

**Scheme 87:**
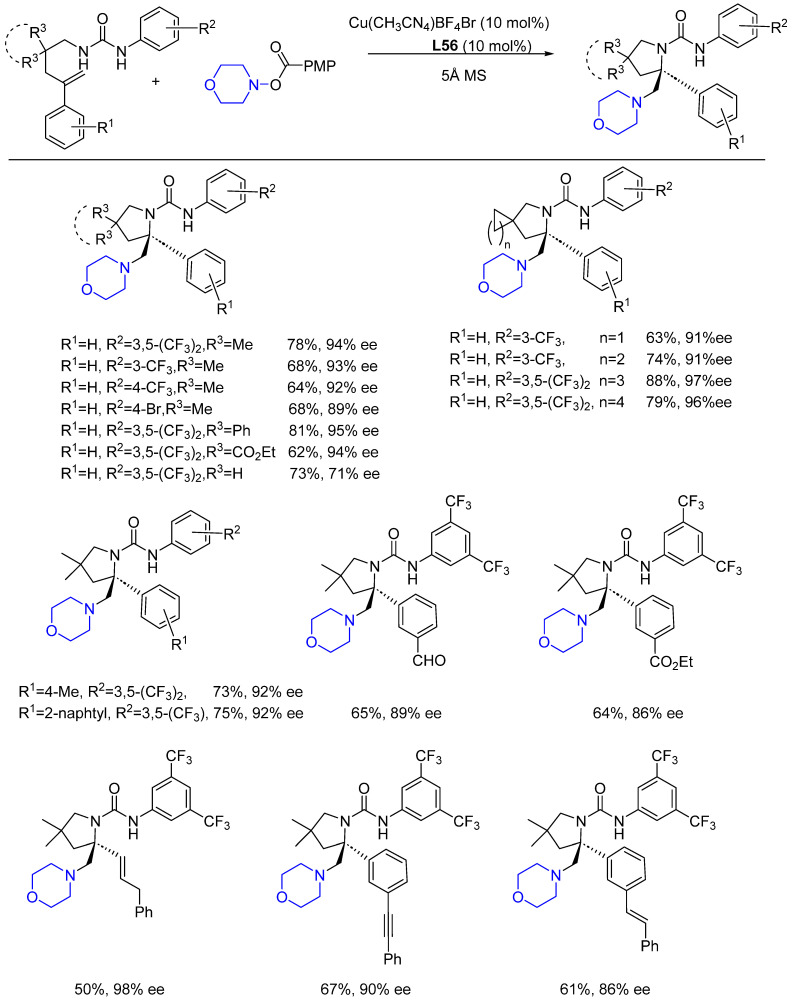
Diamination of styrenes.

**Scheme 88:**
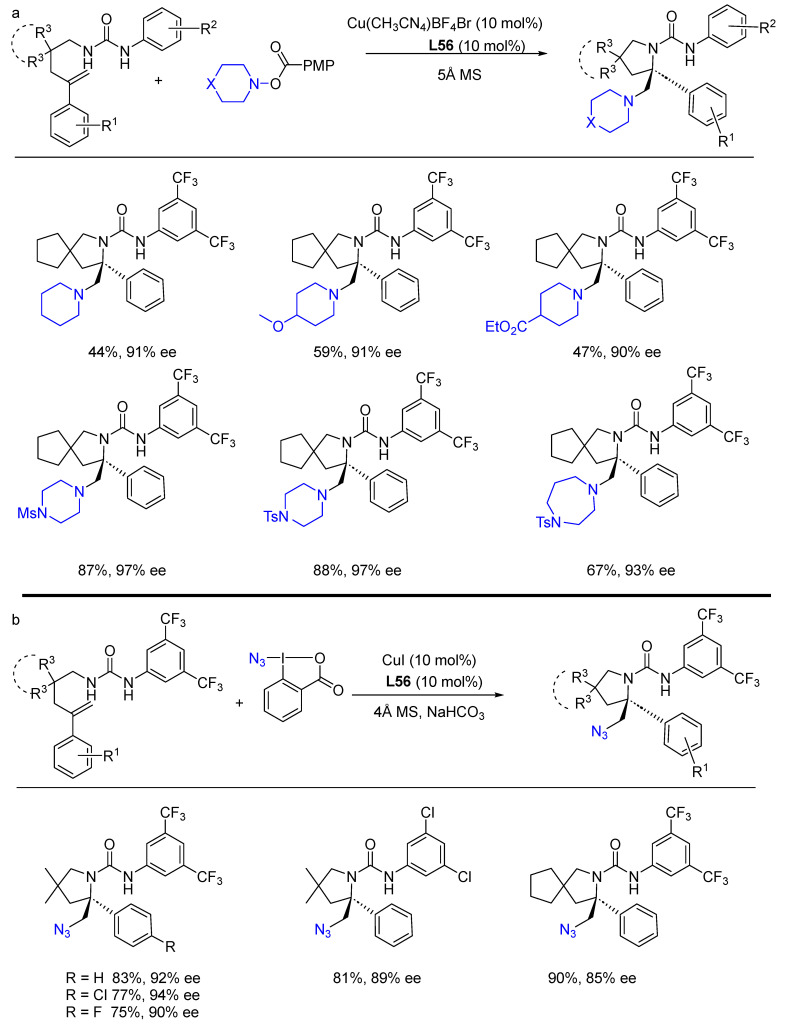
Diamination (**a**) and azidoamination (**b**) of styrenes.

The same system was used for the diamination and aminoazidation [98]. As a second reactant was used, various *O*-acyl hydroxylamines for the diamination and azidoiodinane for the aminoazidation were also used; the CPA **L56**/Cu catalytical system was responsible for the highly asymmetric induction (Scheme 87 and Scheme 88).

**Scheme 89:**
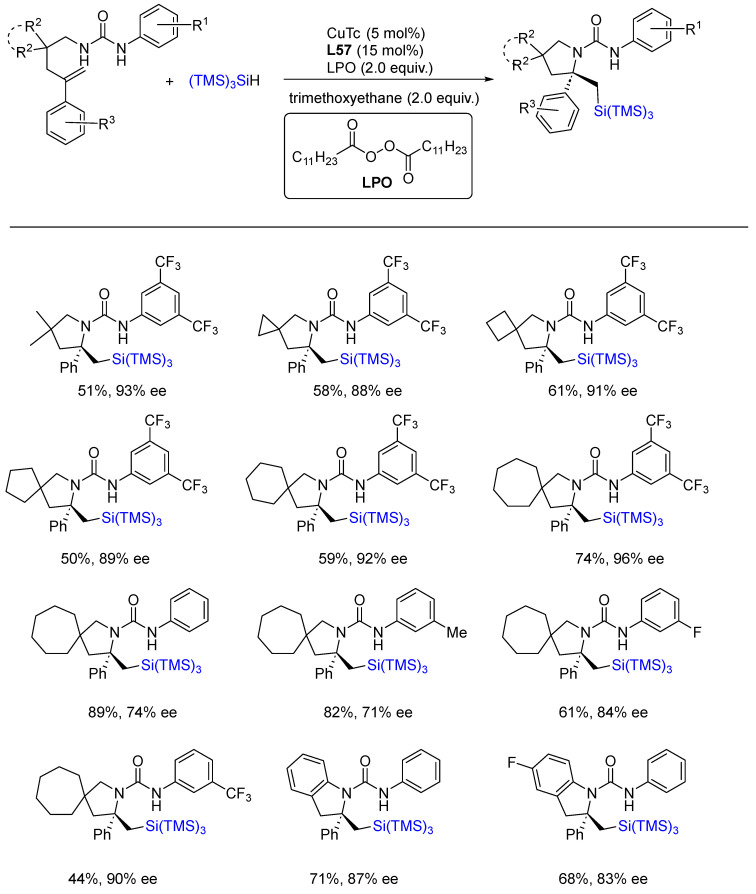
Synthesis of silylated azaheterocycles.

The modification of the above-described conditions allowed for the synthesis of silylated azaheterocycles from the same type of substrates in the presence of CPA **L57**/Cu complex [99]. The reaction proceeds with good chemical yields and ee’s (Scheme 89). The catalytic cycle supposedly involves the formation of an alkyl radical from LPO, which reacts with (TMS)_3_SiH to give a silicon-centered radical through hydrogen atom abstraction (HAA).

**Scheme 90:**
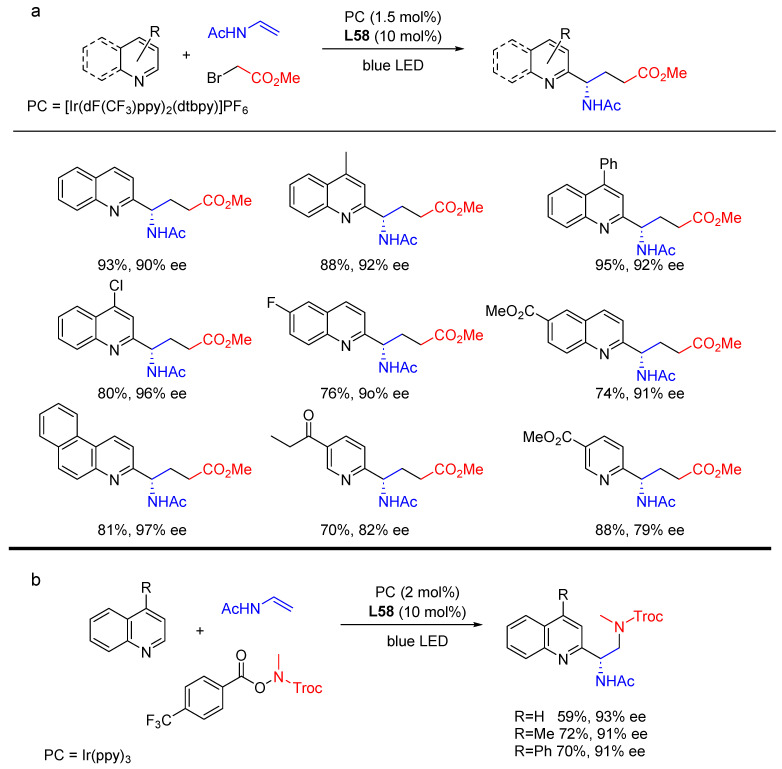
Synthesis of γ-amino acids and diamines by three-component Minisci reaction.

The enantioselective three-component Minisci reaction was developed by Zheng and Studer [100]. The reaction of heteroarene, enamide and methyl 2-bromoacetate catalyzed by CPA **L58** in the presence of iridium photocatalyst provided γ-amino acids with good yields and good-to-very-good selectivities (Scheme 90a). The replacement of the bromoacetate with *O*-acyl hydroxylamine allowed for the synthesis of diamines, also with high ee’s (Scheme 90b). This is the first example of three-component Minisci reaction involving an amidyl radical. 

**Scheme 91:**
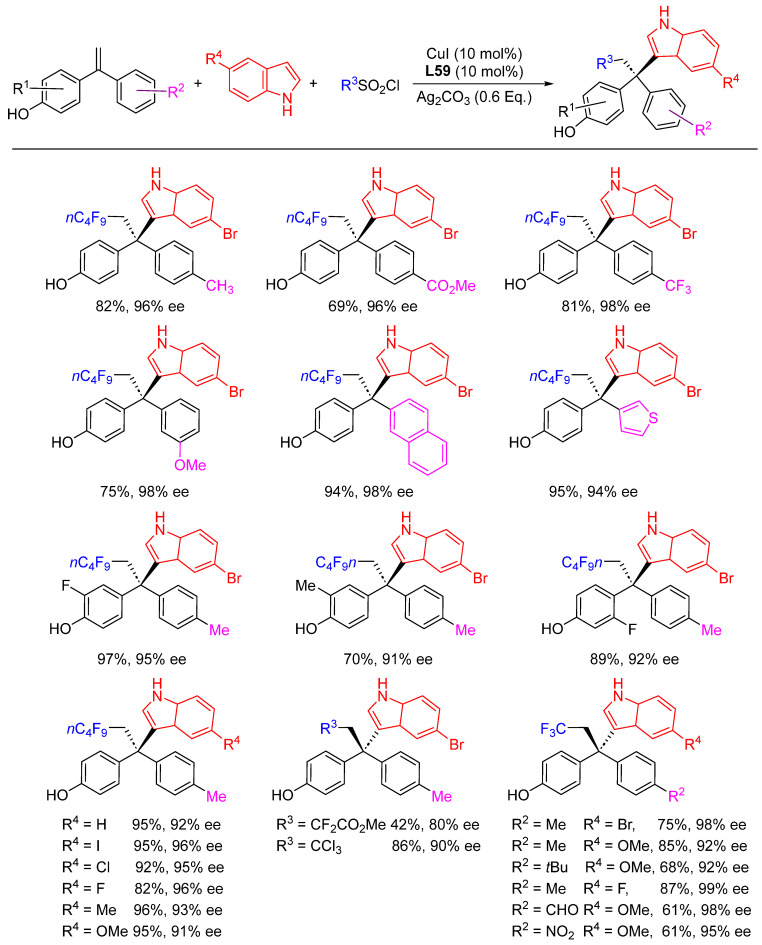
Dicarbofunctionalization of 1,1-diaryloalkenes.

The enantioselective dicarbofunctionalization of 1,1-diaryloalkenes with indole derivatives and halogenated sulfonyl chlorides furnished triarylmethanols bearing a quaternary stereogenic center [101]. The CPA **L59**/Cu(I) complex gave the best ee’s; several other SPINOL- and BINOL-based chiral phosphoric acids were much less efficient, both in terms of chemical yield and asymmetric induction. Various perfluorinated and perchlorinated substituents were introduced using sulfonyl chlorides; however, the trifluoromethyl group had to be derived from Togni’s reagent, because CF_3_SO_2_Cl was inefficient. Again, the use of silver carbonate allowed for the suppression of background reactions (vide supra). Selected examples are presented in Scheme 91.

**Figure 16:**
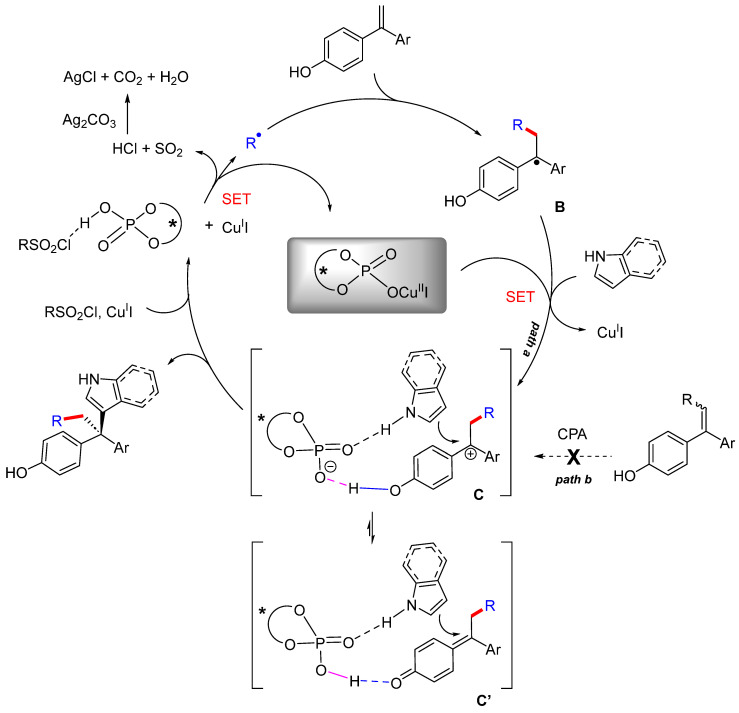
Mechanism proposed for the dicarbofunctionalization of 1,1-diaryloalkenes.

The mechanism of the reaction was studied via a radical quenching experiment, GC-MS, a linear relationship study between product enantiopurity and catalyst enantiopurity, which indicates the involvement of CPA catalyst in the enantioselectivity-determining transition states. The presence of intermediate C is also supported by DFT calculations. The proposed mechanism is depicted below in Figure 16. 

**Scheme 92:**
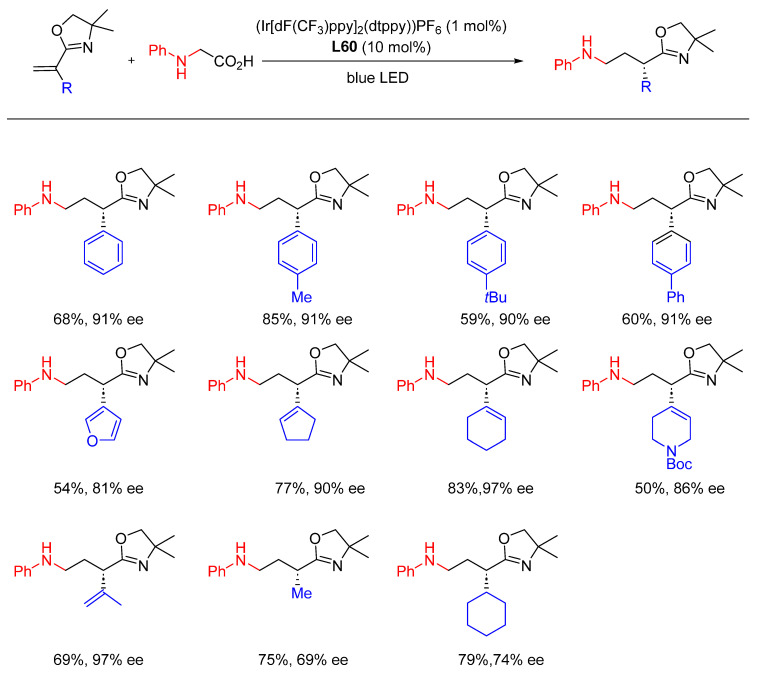
Functionalization of alkenyl oxazolines with *N*-aryl glycines.

Chen et al. presented a method for the functionalization of alkenyl oxazolines with *N*-aryl glycines in the presence of CPA **L60** as a chiral catalyst and iridium complex (Ir[dF(CF_3_)ppy]_2_(dtppy))PF_6_ as a photocatalyst [102]. The reaction yielded α-substituted oxazoline with good yields and high ee’s (Scheme 92).

**Scheme 93:**
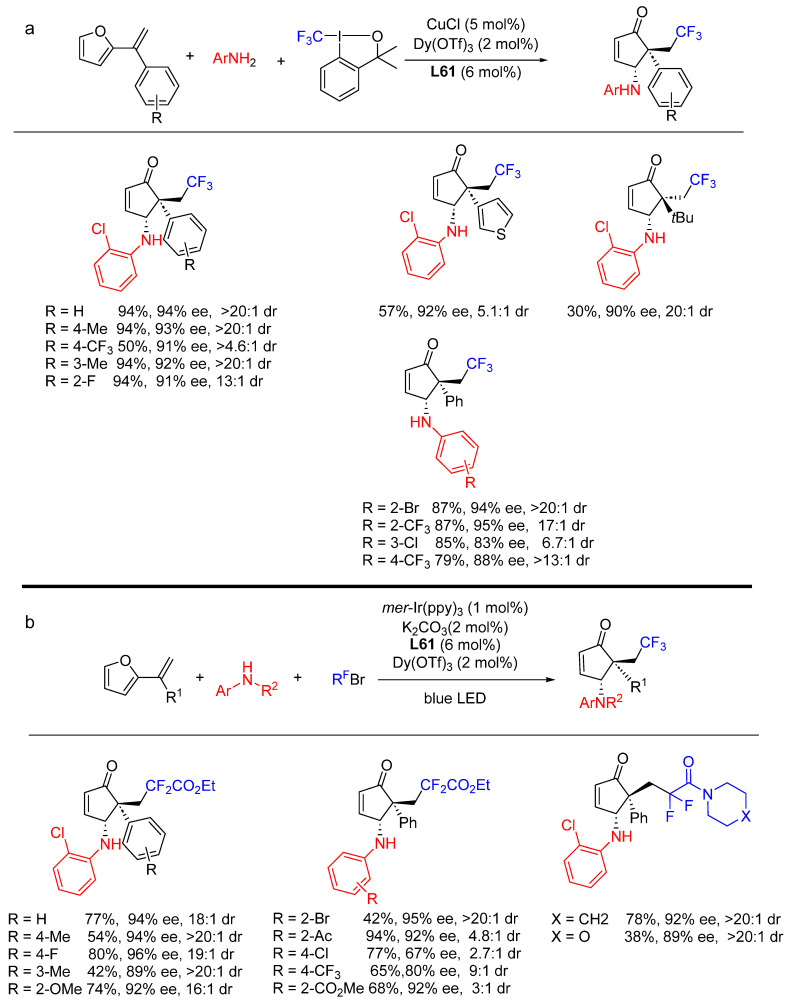
Three-component Piancatelli-type rearrangement.

The three-component Piancatelli-type rearrangement catalyzed using a combination of chiral CPA **L61**/copper(I) and achiral Lewis acid Dy(OTF)_3_ was developed by Xu et al. [103]. The reaction of furylalkenes, anilines and Togni-I reagent led to a series of cyclopentenones with a quaternary stereogenic center, substituted with the CF_3_CH_2_ group and an arylamine with very good yields and diastereo- and enantioselectivity (Scheme 93a). To introduce halogenated substituents other than trifluoromethyl, it was necessary to use the appropriate bromides instead of the Togni reagent and the iridium photocatalyst (Scheme 93b).

**Scheme 94:**
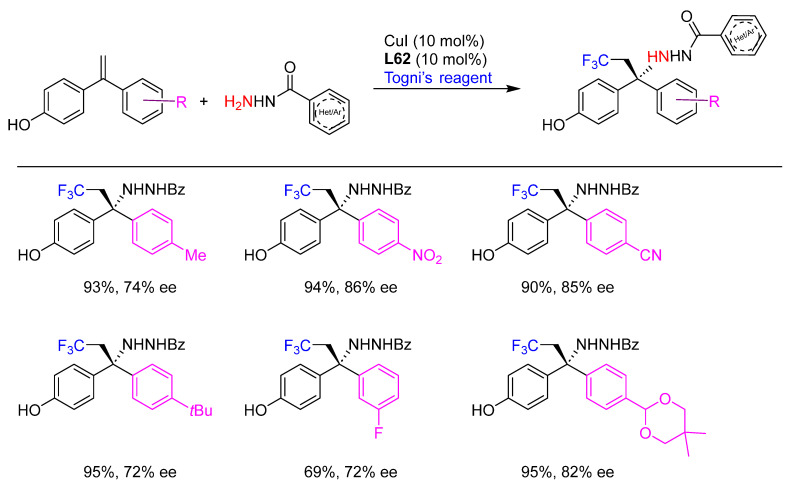
Intermolecular aminotrifluoromethylation of 1,1-diarylalkenes.

An interesting modification of the previously described intramolecular approach [96] was presented by Liu [104]. In this work, the group described the development of the first efficient asymmetric intermolecular radical aminotrifluoromethylation of 1,1-diarylalkenes with nitrogen-based nucleophile and Togni’s reagent enabled by CPA **L62**/Cu(I) cooperative catalysis with very good chemical yields and moderate-to-good ee’s (Scheme 94).

**Scheme 95:**
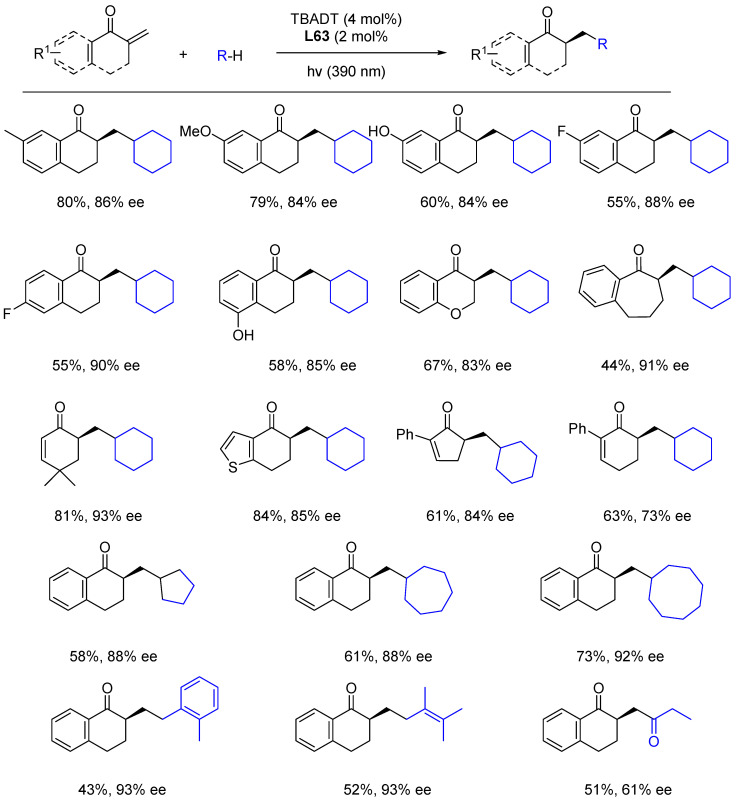
C(sp3)−H bond addition to enones.

Dai et al. described an asymmetric photocatalytic C(sp^3^)−H bond addition to enones using tetrabutylammonium decatungstate (TBADT) as a hydrogen atom transfer (HAT) photocatalyst and chiral phosphoric acid **L63** [105]. Products of the substitution were obtained with good yields and good to high enantioselectivities (Scheme 95).

**Scheme 96:**
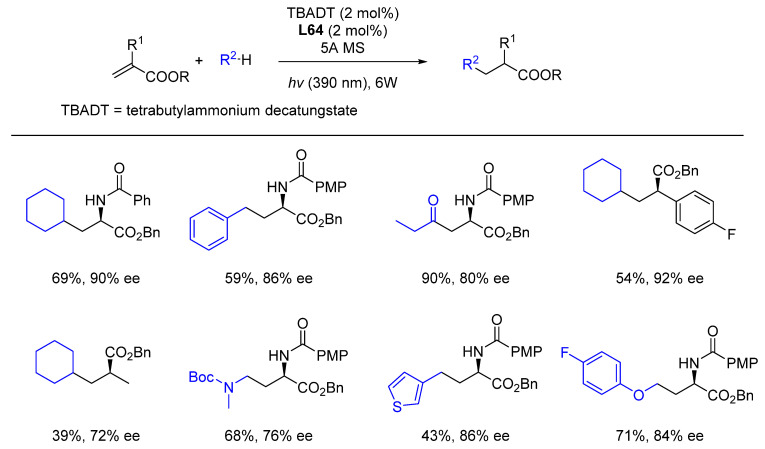
C(sp^3^)−H bond addition to α-substituted acrylates.

The same approach to the asymmetric photocatalytic C(sp^3^)−H bond addition was applied to the functionalization of α-substituted acrylates using a chiral phosphoric acid **L64** as a chirality source [106]. The process occurs via a radical/ionic relay process, including a TBADT-mediated HAT to cleave the inert C(sp^3^)−H bond, a 1,4-radical addition, a back hydrogen abstraction, and an enantioselective protonation. With respect to the hydrocarbon substrates, the reaction tolerated a wide range of cyclic hydrocarbons and methylarenes and the substituted products were obtained with moderate yields and good enantioselectivities (Scheme 96).

**Scheme 97:**
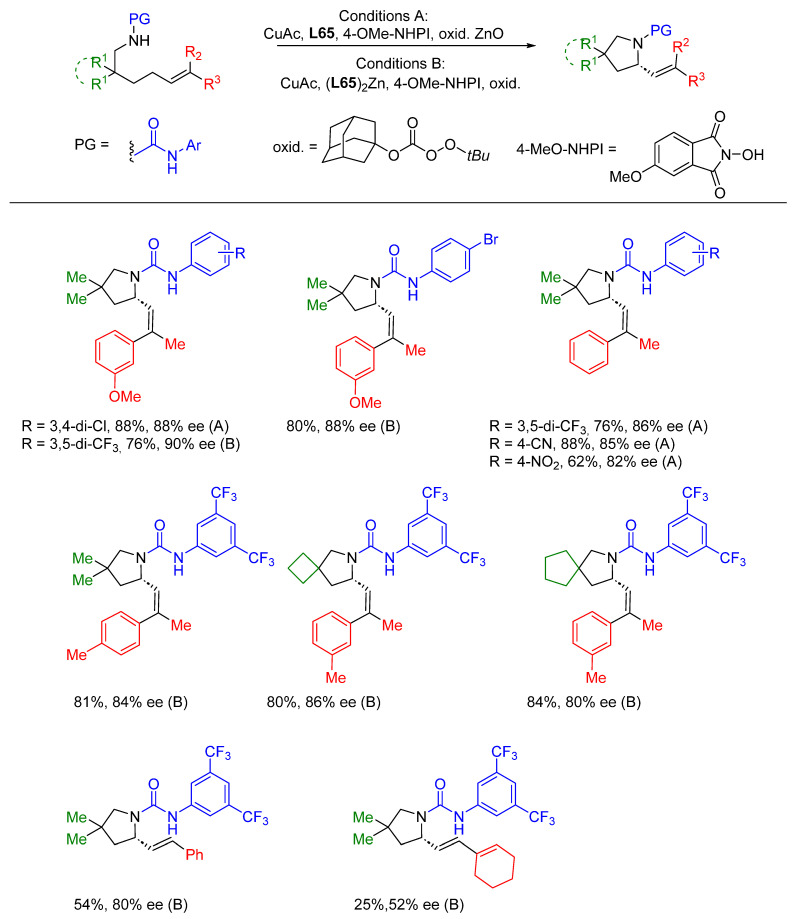
Intramolecular C-H amination in allylic position.

**Scheme 98:**
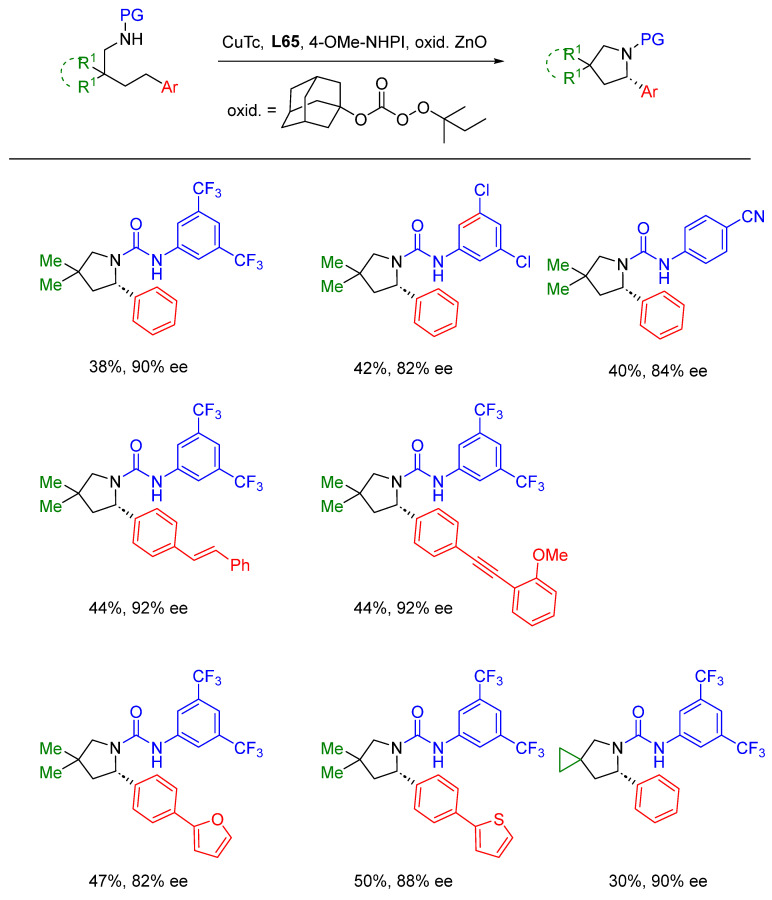
Intramolecular C-H amination in benzylic position.

Ureas, successfully used in the intermolecular aminations of the double bond (vide supra), were also valuable substrates in the intramolecular, enantioselective C-H amination in the presence of CPA **L65**/Cu(I)/Zn(II) catalyst, oxidant and 4-OMe-PINO as the chemoselective HAT mediator, which abstracts a proton in allylic and benzylic position [107]. The Zn(II) was added in the form of ZnO and it supposedly built zinc phosphate with CPA. Alternatively, (**L65**)_2_Zn was preformed by the reaction of CPA with Et_2_Zn.The latter further improved the enantioselectivity of the reaction. The selected examples are presented in Scheme 97 (allylic) and Scheme 98 (benzylic).

**Figure 17:**
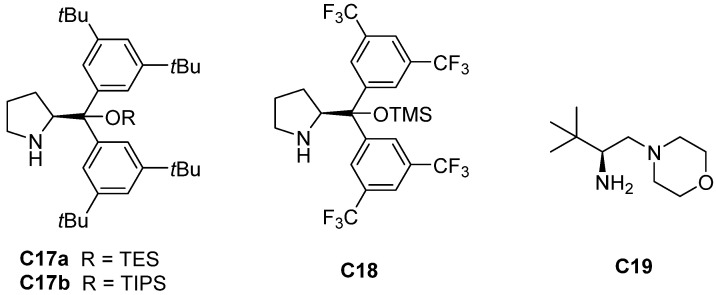
Amine organocatalysts.

**Scheme 99:**
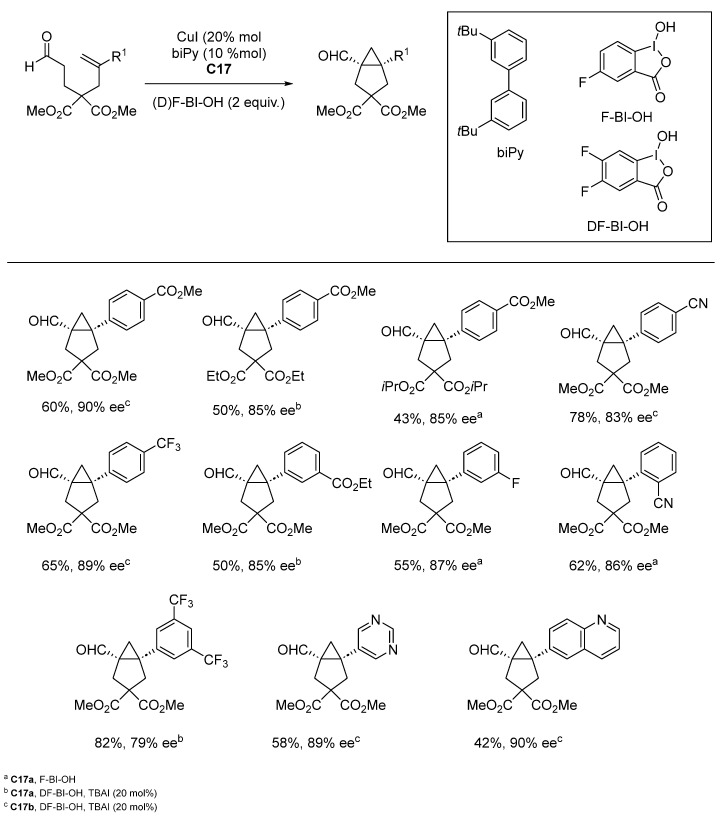
Intramolecular cyclopropanation.

Intramolecular cyclopropanation was developed by Liu [108]. The concept is based on the classical organocatalytic approach, i.e., the formation of an enamine in the reaction of an unsaturated aldehyde with the proline-derived organocatalyst followed by a radical formation and ring closure to give bicyclo[3.1.0]hexanes. The best chiral catalysts were selected from among 15 amines and the best asymmetric induction was observed for the bulky proline derivatives **C17a** and **b** (Figure 17); however, only in the presence of two other crucial elements—bipyridyl copper ligand biPy and hypervalent iodine(III) reagents as sufficiently strong oxidants F-BI-OH and DF-BI-OH (Scheme 99).

**Scheme 100:**
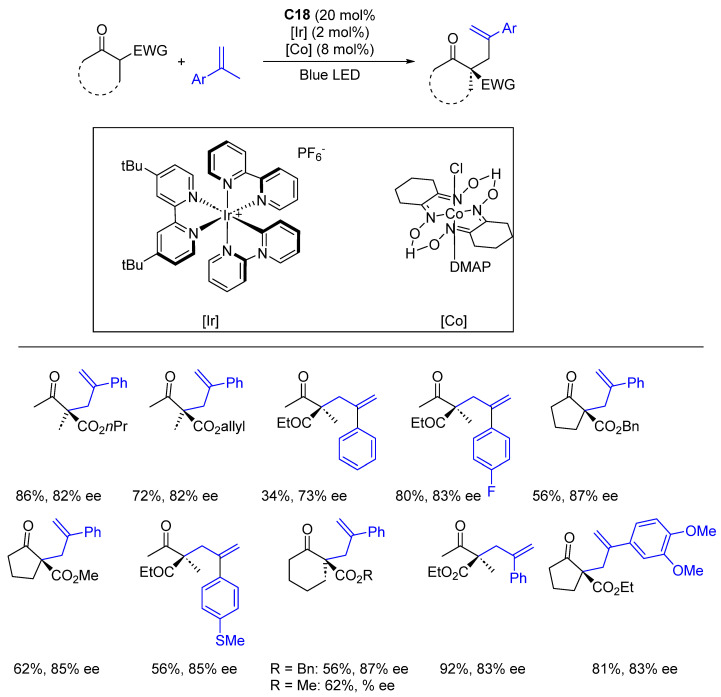
Dehydrogenative coupling of β-ketocarbonyls with alkenes.

A simple amine, the *tert*-leucinol derivative, was used as a chiral tool for the enamine formation. The morpholine substituent appeared to be the most efficient for the asymmetric dehydrogenative coupling of β-ketocarbonyls and simple olefins with acceptable yields and stereoselectivity, as depicted in Scheme 100 [109]. The radical addition process involving a chiral α-imino radical, iridium photocatalyst Ir(ppy)_2_dtbbpyPF_6_ and a cobaloxime Co(II)-metalloradical resulted in stereoselective C−C bond formation.

## 4. Organocatalysis

Organocatalyzed reactions are now an emerging tool in asymmetric radical synthesis. While dual organo- and transition-metal catalysis brought several excellent publications (vide supra), the organocatalysis, which does not suffer from tedious transition-metal removal, is a method of choice for the pharmaceutical industry. In recent publications in the radical organocatalysis area, there are two classes of catalysis that dominate—Brønsted acids, typically chiral phosphoric acids and amine catalysis, usually based on the proline derivatives, often accompanied by organic photosensitizers. Let us start with the former.

### 4.1. Chiral Phosphoric Acids as Catalysts

**Figure 18:**
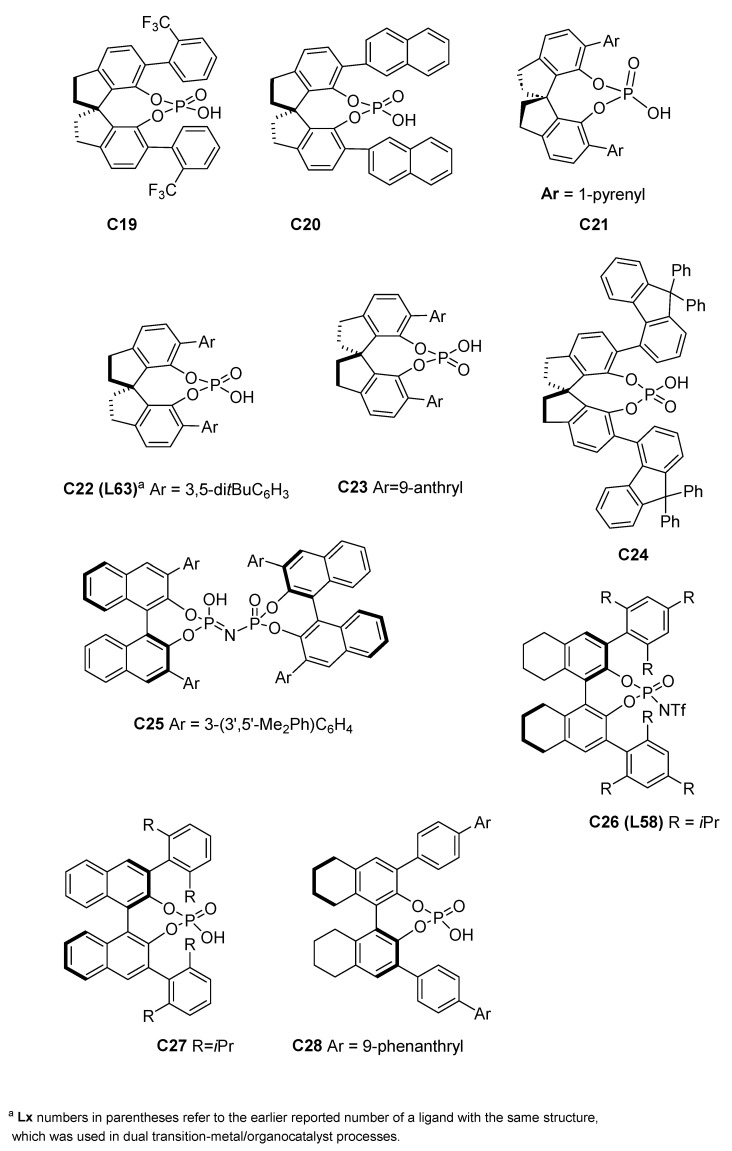
Chiral phosphoric acids as catalysts.

**Scheme 101:**
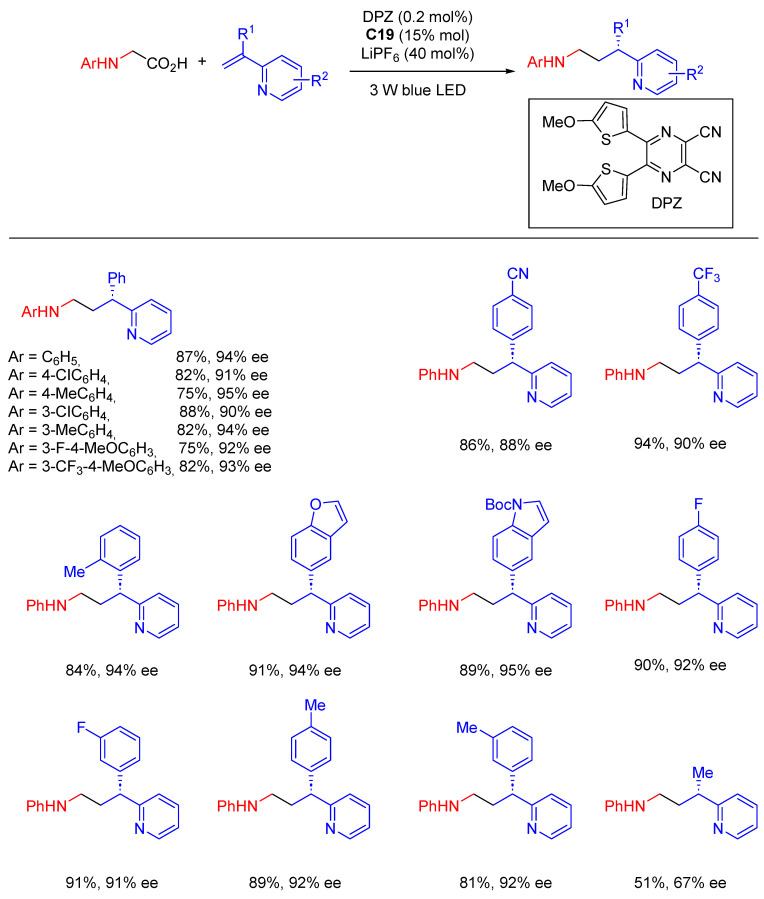
Conjugate addition of *N*-aryl glycines to α-substituted 2-vinyl pyridines.

**Scheme 102:**
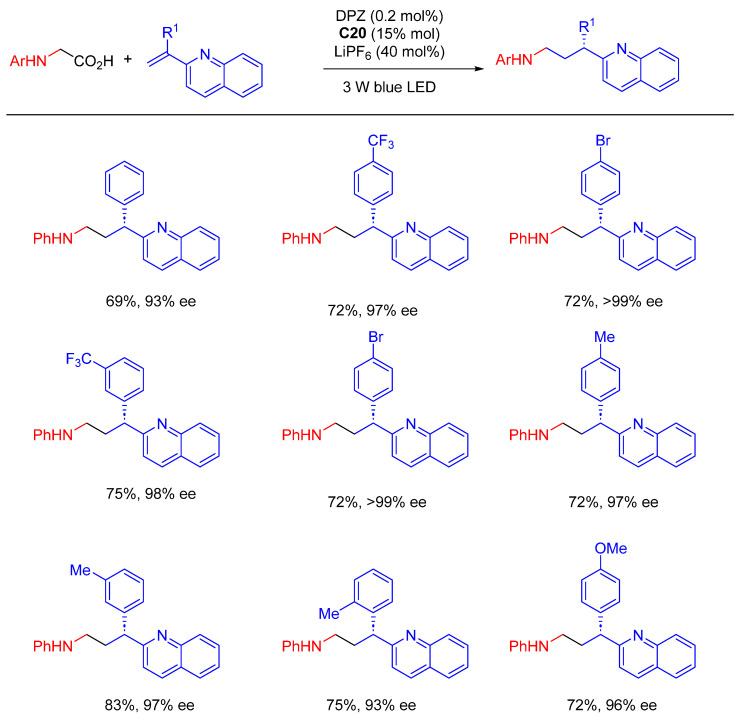
Conjugate addition of *N*-aryl glycines to α-substituted 2-vinyl quinolines.

Jiang and co-workers described a series of papers dealing with organocatalytic, radical functionalization of the α-position in *N*-aryl glycines. The conjugate addition of *N*-aryl glycines to α-substituted 2-vinyl pyridines and 2-vinyl quinolines proceeded very well, when catalyzed by SPINOL-based phosphoric acid **C19** and **C20**, respectively (Figure 18), in the presence of DPZ as a photocatalyst [110]. A series of 3-heteroaryl-3-substituted amines was obtained with very-good-to-excellent enantioselectivity, especially for 2-vinyl quinolines (Scheme 101 and Scheme 102). As suggested on the basis of theoretical and experimental data, the stereogenic center is formed by the enantioselective protonation by CPA.

**Scheme 103:**
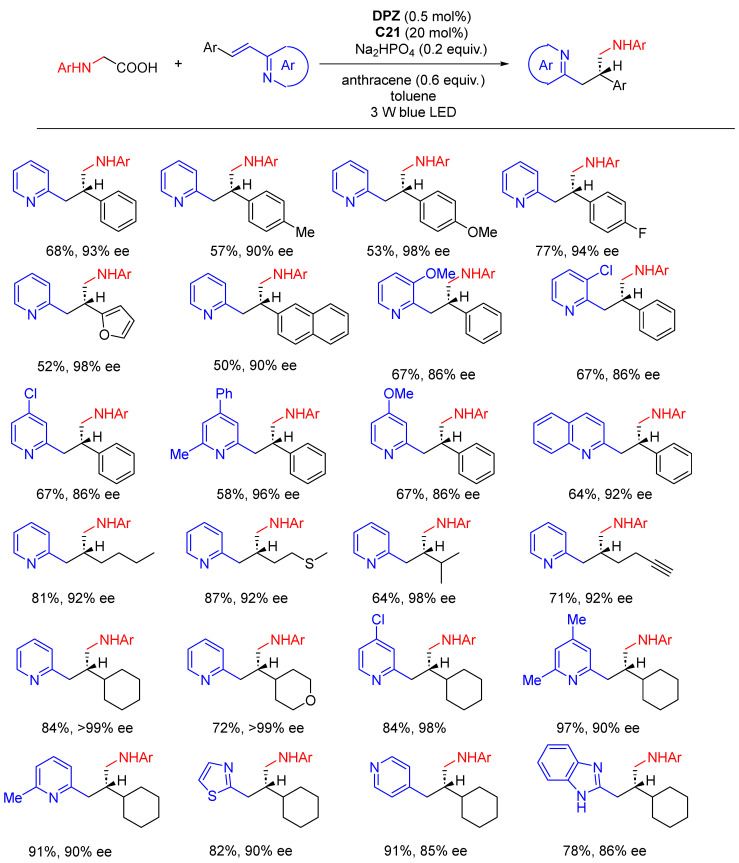
Conjugate addition of *N*-aryl glycines to β-substituted 2-vinyl pyridines.

Also, β-substituted pyridines could be used for the enantioselective addition of *N*-aryl glycines and the reaction catalyzed by CPA **C21** and DPZ afforded various heteroaromatics bearing the tertiary stereogenic centers with good-to-excellent ee’s [111]. The selected examples are presented in Scheme 103.

On the basis of experiments and calculations, some possible mechanisms of the reaction were proposed.

**Scheme 104:**
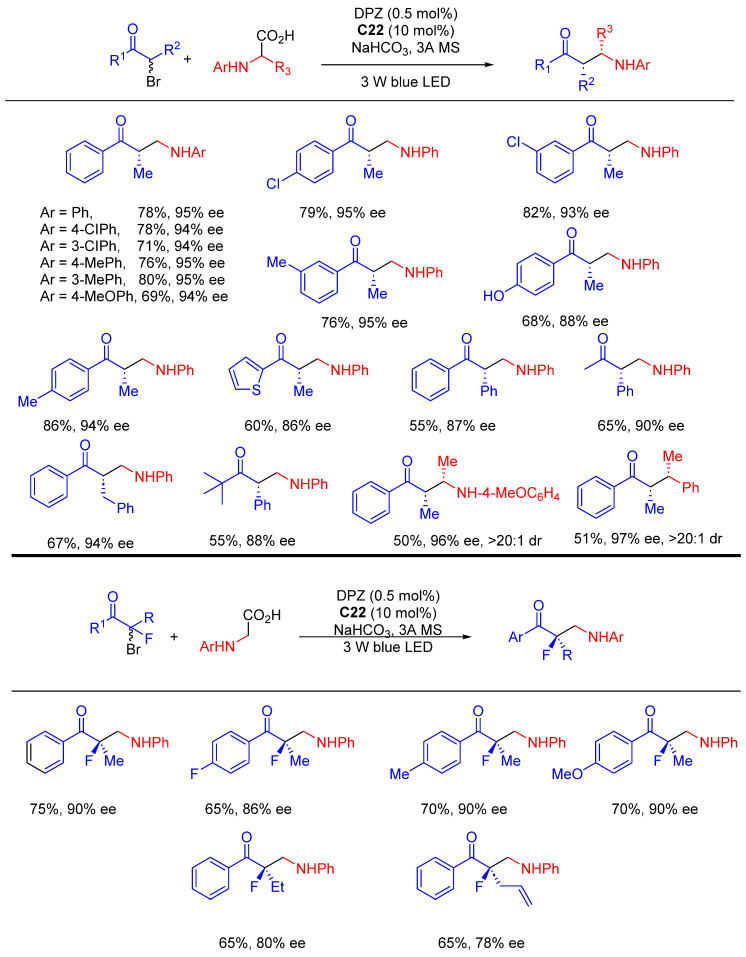
Substitution of alkyl halides with *N*-aryl glycines and amino acids.

Another example is the enantioconvergent substitution of racemic alkyl halides, being part of α-bromo ketones with *N*-aryl glycines and amino acids [112]. The reaction is based on the concept of the debromination of the ketone leading to the alkyl radical and subsequent cross-coupling with another radical derived from the amino acid in the chiral environment of the difunctional H-bonding CPA catalyst **C22**. Both secondary and tertiary stereogenic centers are formed with a high selectivity (Scheme 104).

**Scheme 105:**
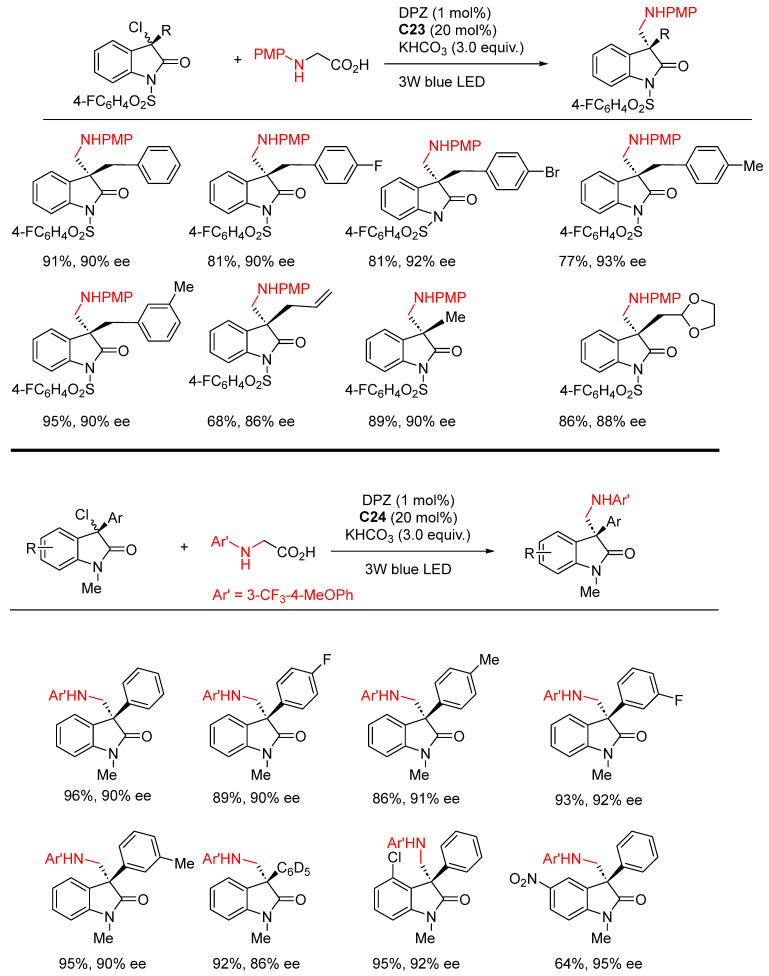
Substitution of 3-substituted 3-chlorooxindoles.

That approach was extended to the substitution of 3-substituted 3-chlorooxindoles with high yields and selectivities [113]. Some selected examples are presented in Scheme 105.

**Scheme 106:**
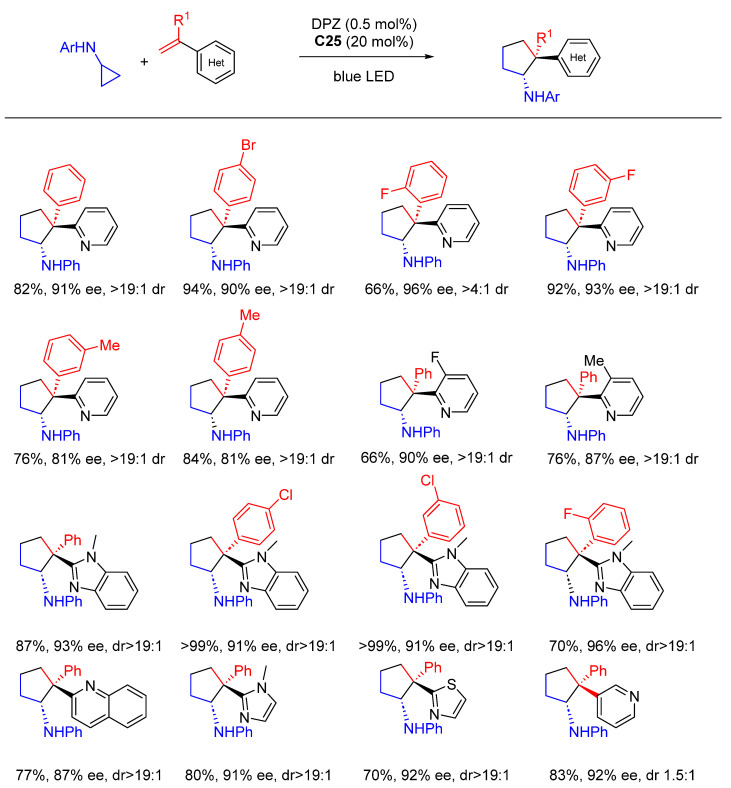
Synthesis of azaarene-substituted cyclopropanes.

On the basis of previous research, Jiang developed a new enantioselective process leading to an azaarene-substituted cyclopentane ring with a quaternary stereogenic center [114]. Formally, [3+2]cycloaddition, the reaction is in fact a photocatalyzed cyclopropylamine ring opening, followed by two radical additions. The chiral C2-symmetric imidodiphosphoric acid **C25**, which stabilizes the transition state by hydrogen bonding to both nitrogen atoms, is responsible for the asymmetric induction. The minimum energy of the TS was calculated and, according to the results, the minimalization of steric interaction between the aryl groups at the catalyst and the one originating from the cyclopropyl amine are responsible for the observed selectivity. Various cyclopentanes were obtained in good chemical yields and high ee’s (Scheme 106).

**Scheme 107:**
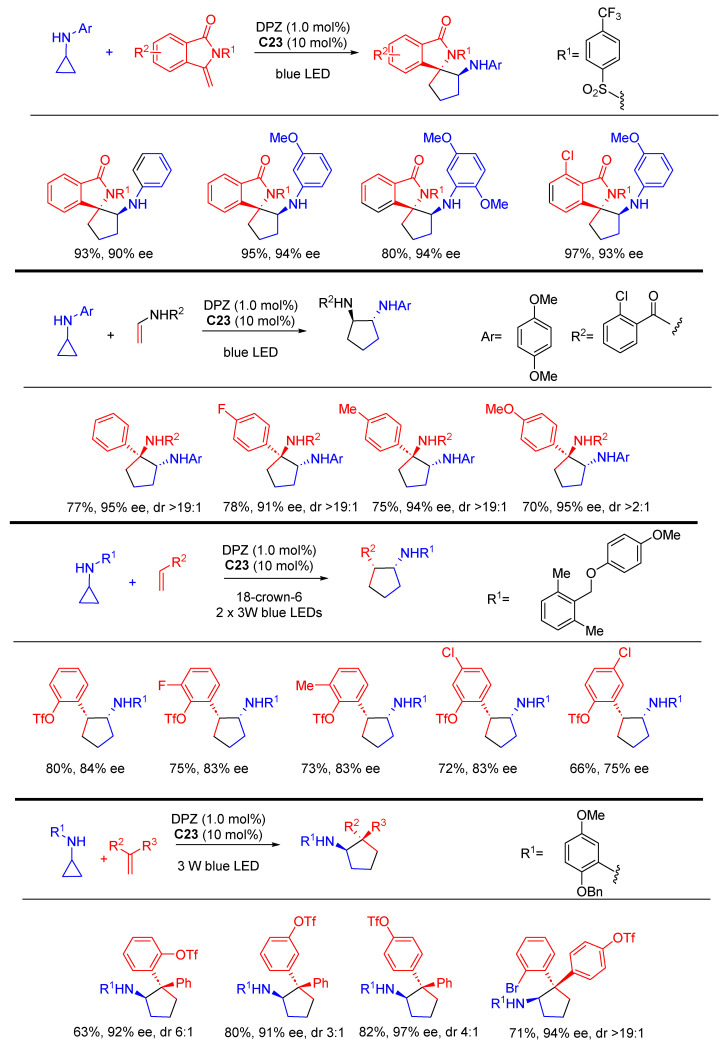
[3+2]Photocycloadditions of cyclopropylamines with electron-rich and electron-neutral olefins.

Jiang made this method more general and described the highly stereoselective [3+2] photocycloadditions of cyclopropylamines with electron-rich and electron-neutral olefins [115]. Herein, due to a lack of the second binding point, a much simpler CPA, **C23**, was used with good results. Quite a number of olefins were tested as substrates and the representative examples are presented in Scheme 107.

**Scheme 108:**
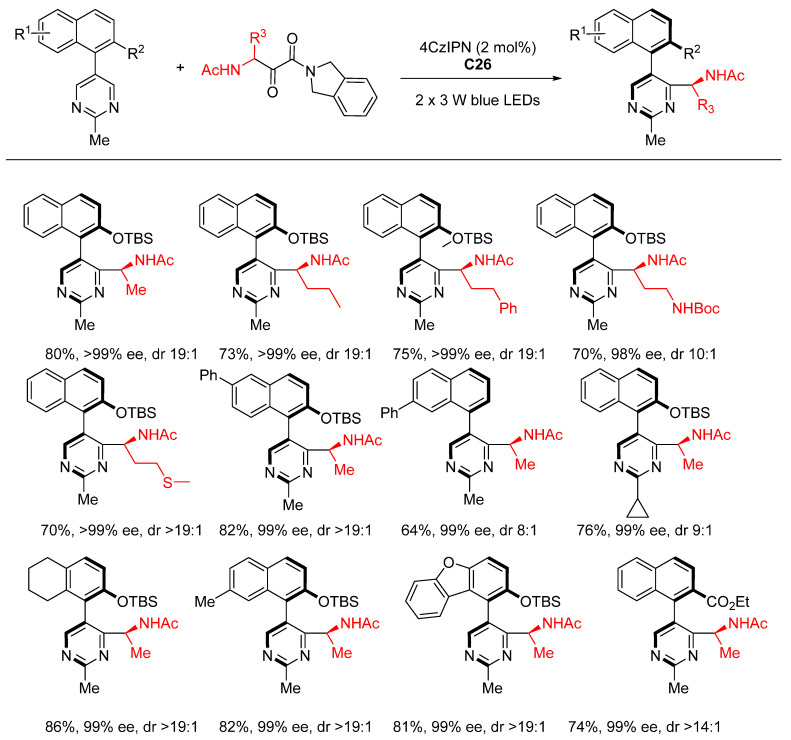
Synthesis of axially and centrally chiral heterobiaryls.

The Minisci reaction was used in the synthesis of axially and centrally chiral heterobiaryls of 5-arylpyrimidines and α-amino acid esters, repeatedly with an enantioselectivity over 99% [116]. The chiral environment leading to high ee’s is secured by chiral phosphoric acid **C26/L58** (other substituents at the phenyl ring were less efficient) and the formation of the radical is controlled by organic photocatalyst 4CzIPN. Several biaryls were obtained and those with the highest ee are presented in Scheme 108.

**Scheme 109:**
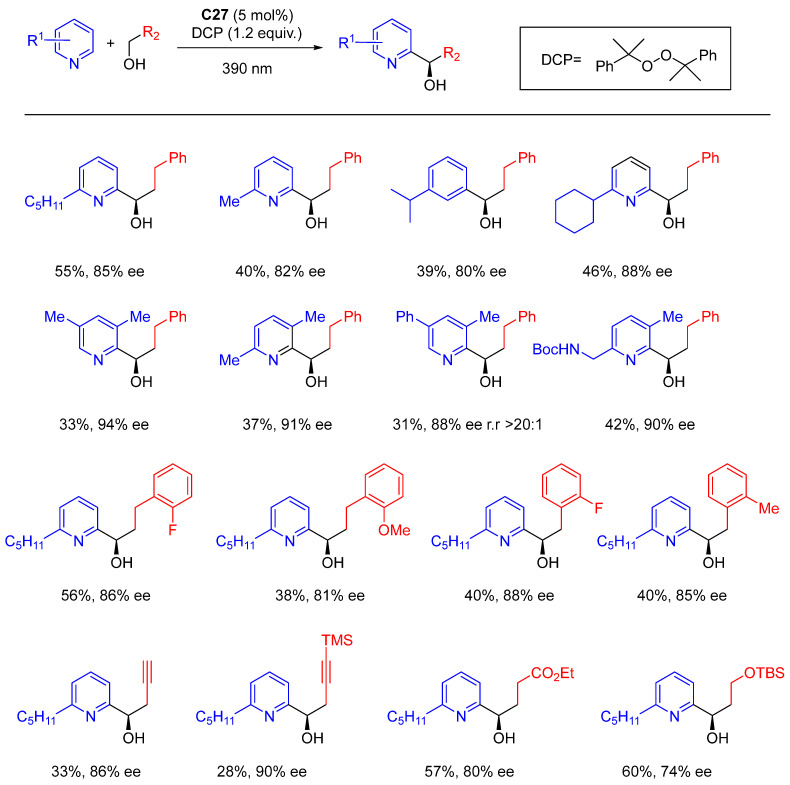
Minisci reaction using alcohols as prochiral radical source.

The new version of the Minisci reaction was developed by Phipps. Unlike the earlier presented approaches, he used alcohols as a prochiral radical source, for the first time accessing secondary alcohols with good yields and enantiomeric excesses [117]. The reaction proceeds best with the irradiation at 390 nm, and under these conditions, dicumyl peroxide (DCP) was sufficient and no additional photosensitizer was required. Interestingly, less hindered CPA **C27** was proved to furnish better ee’s than **C26** (Scheme 109).

**Scheme 110:**
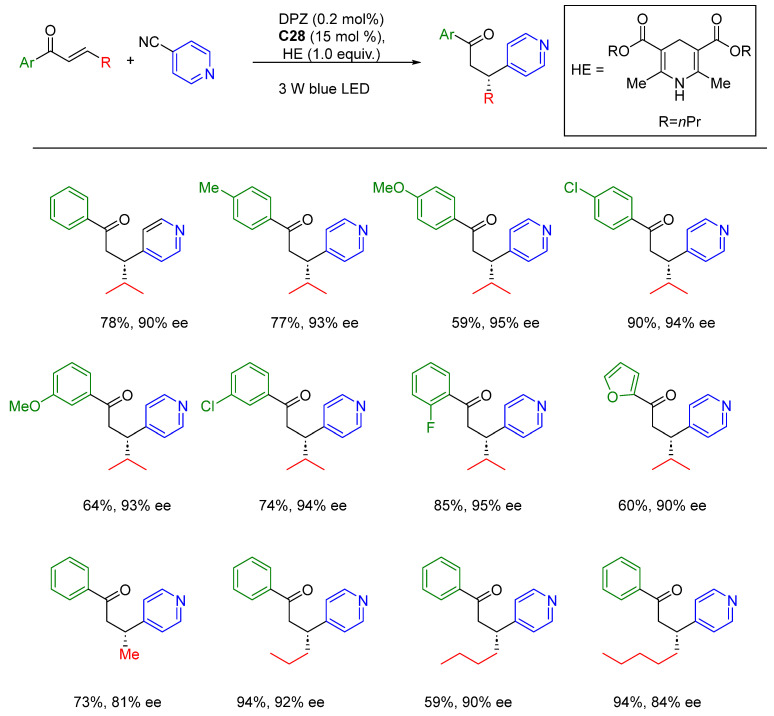
Addition of cyanopyridines to acyclic and cyclic enones.

Another publication, devoted to the conjugated addition to enones, shows how much effort needs to be put in and how many catalysts need to be tested to obtain the best stereoselectivity. Li et al. developed an efficient method for the addition of cyanopyridines to acyclic and cyclic enones [118]. They tested 51 chiral catalysts, several photoredox catalyst, both metal-based and organic, and seven Hantzsch esters as reductants. The addition to β-alkyl ketones proceeded with a high enantioselectivity in the presence of CPA **C28** (Scheme 110).

However, in the presence of **C28**, addition to β-aryl enones (chalcones) led to nearly racemic products with a low chemical yield. Further investigation revealed that in this case, a totally different catalytic system was required, and its main element was a chiral catalyst from a different family of organocatalyst—a chiral thiourea. This is what leads us to the next section.

### 4.2. Double H-Bonding Catalysts

**Figure 19:**
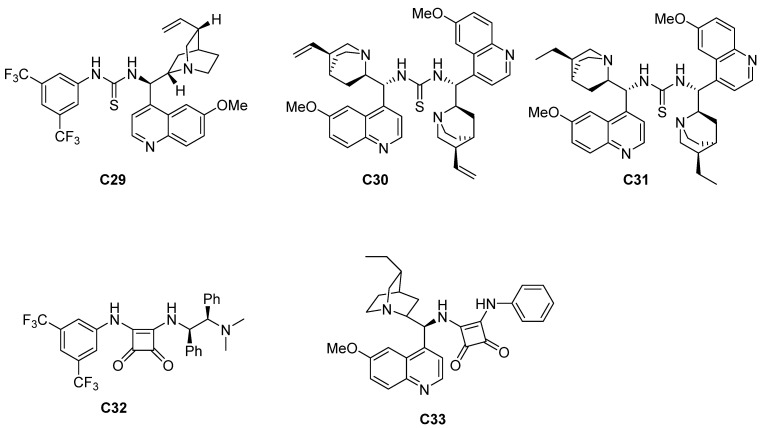
Chiral double-H-bonding catalysts.

The quinine-derived chiral thiourea **C29** (Figure 19) was shown to be the most efficient organocatalyst for addition to chalcones.

**Scheme 111:**
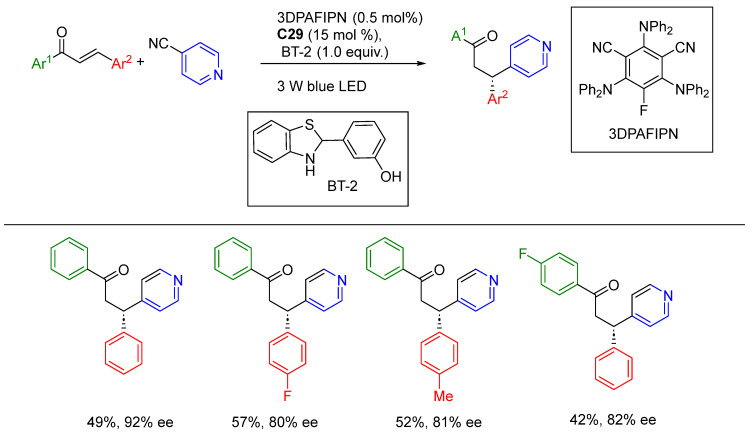
Addition to chalcones.

However, not only the chiral catalyst but also the photosensitizer and amine reductant had to be tuned in order to achieve ee’s slightly over 80% ee (Scheme 111).

**Scheme 112:**
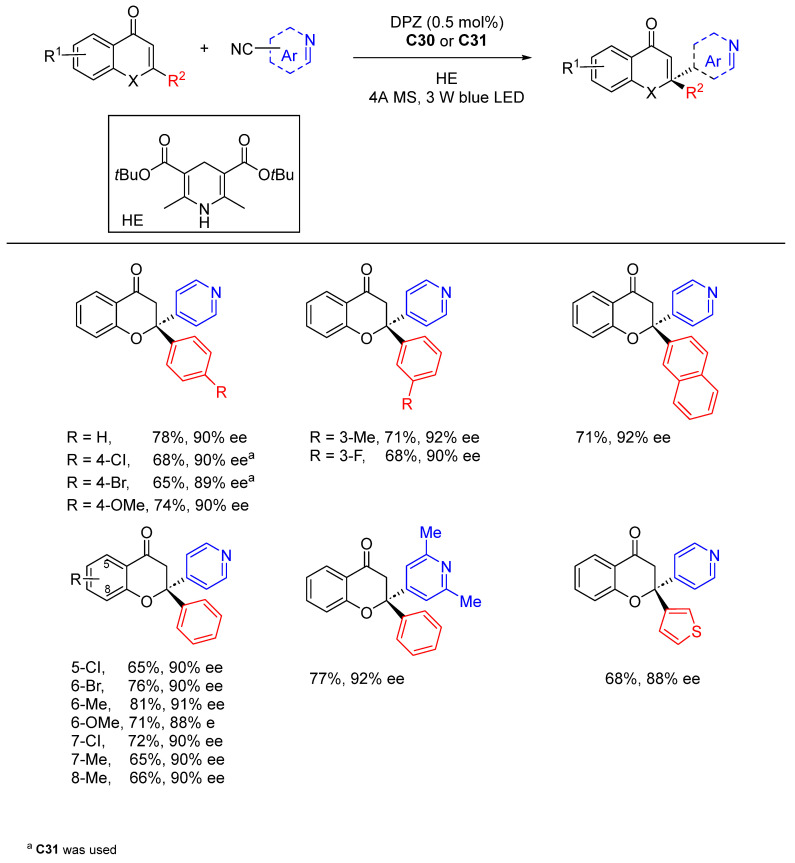
Addition to cyclic enones.

And again, attempts to use the already established catalytical system to cyclic enones failed and some modifications were required; DPZ was used as photocatalyst and the authors returned to the Hantzsch-type ester as amine. The addition proceeded with good enantioselectivities around 90% ee (Scheme 112).

**Scheme 113:**
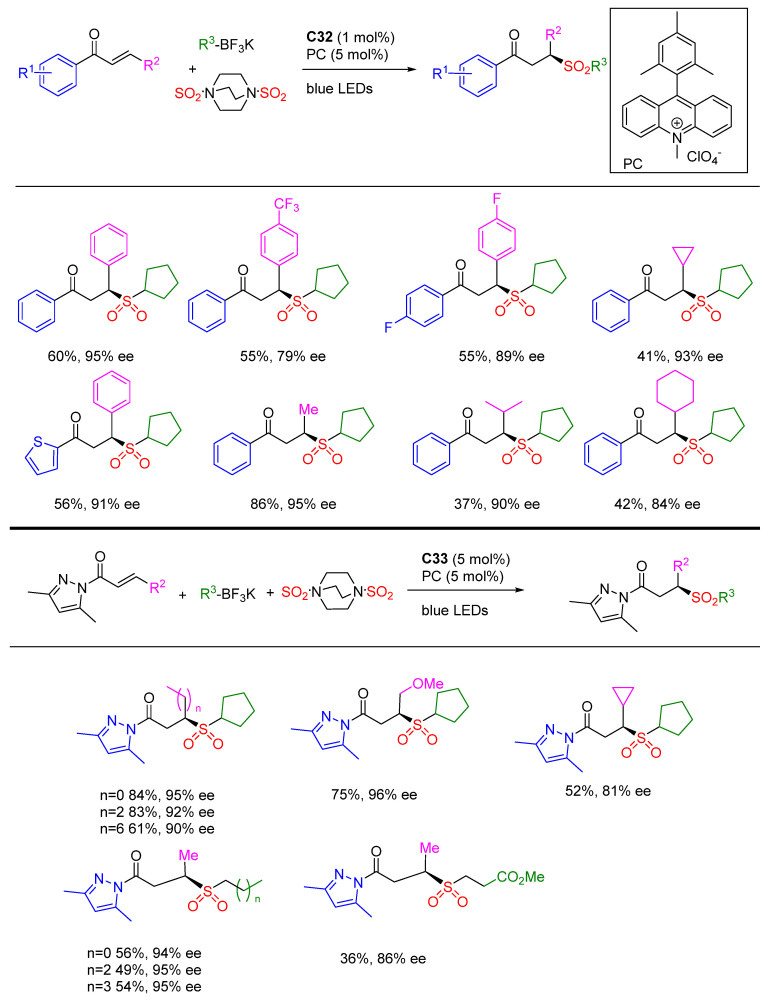
Synthesis of β-carbonyl sulfones by conjugate addition.

Chiral squaramides are another group of very efficient organocatalysts, whose ability to form two hydrogen bonds allows for excellent stereoselectivity in various reactions. He et al. developed a method for the conjugate addition leading to enantioenriched β-carbonyl sulfones [119]. The combination of potassium alkyltrifluoroborates as an alkyl source, and DABCO^.^(SO_2_)_2_ complex as a sulfur dioxide source provided convenient access to chiral sulfones. The reaction performed in the presence of the photocatalyst Mes-Acr+ perchlorate and squaramides **C32** and **C33** yielded various sulfones with high enantioselectivity (Scheme 113).

**Scheme 114:**
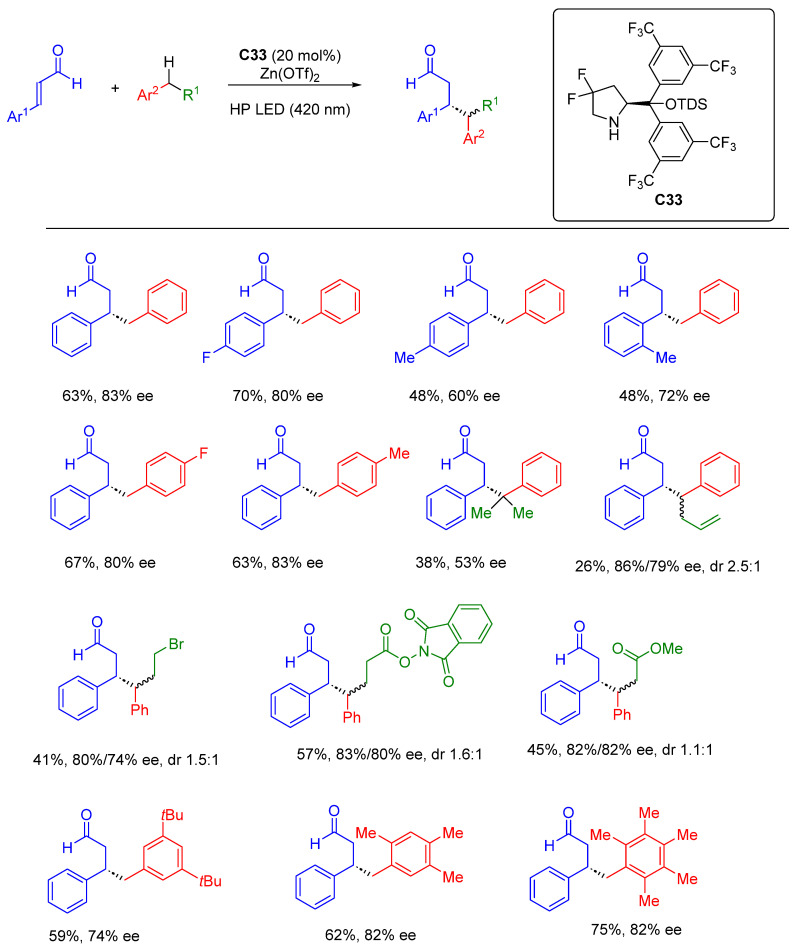
β-Benzylation of enals.

Melchiorre developed an excellent combination of classical amine organocatalysis with light-induced radical chemistry. The group showed that the iminium ion obtained by the reaction of an aldehyde with an amine organocatalyst after irradiation goes to the excited state, which in turn, with a suitable substrate, generates a radical by a SET-oxidation. This approach was used for the functionalization of the unactivated benzylic position in toluene and its derivatives [120]. In the presence of the proline-derived catalyst **C33**, the β-benzylation of enals could be performed, affording products with good chemical yields and enantioselectivities (Scheme 114); however, the reaction is limited to the enals bearing an aromatic ring in the β-position, since the β-alkyl enals are unable to react through the excited state.

**Scheme 115:**
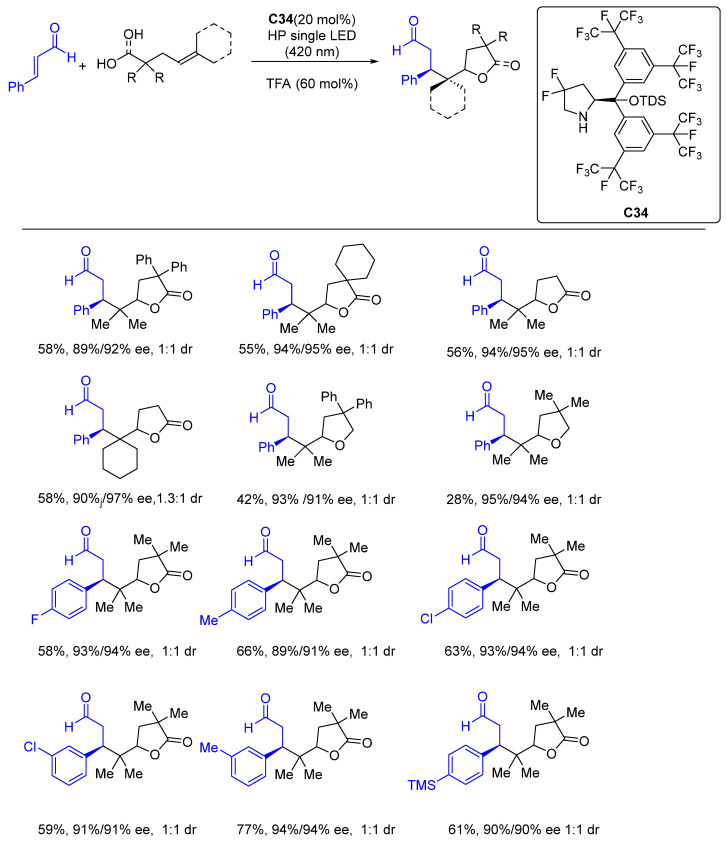
Synthesis of chiral γ-lactones.

Melchiorre extended the use of excited-state reactivity of chiral iminium ions to several other enantioselective processes. The radical cascade reaction of α,β-unsaturated aldehydes (the precursor of the chiral iminium ion) and the alkenes bearing a suitably placed oxygen function (hydroxyl of carboxylic group) led to the chiral γ-lactones [121]. In the presence of a very sterically demanding catalyst **C34**, good yields and high enantioselectivities were obtained, as presented in Scheme 115. The process was extended to the three-component reaction, albeit with lower selectivity.

**Scheme 116:**
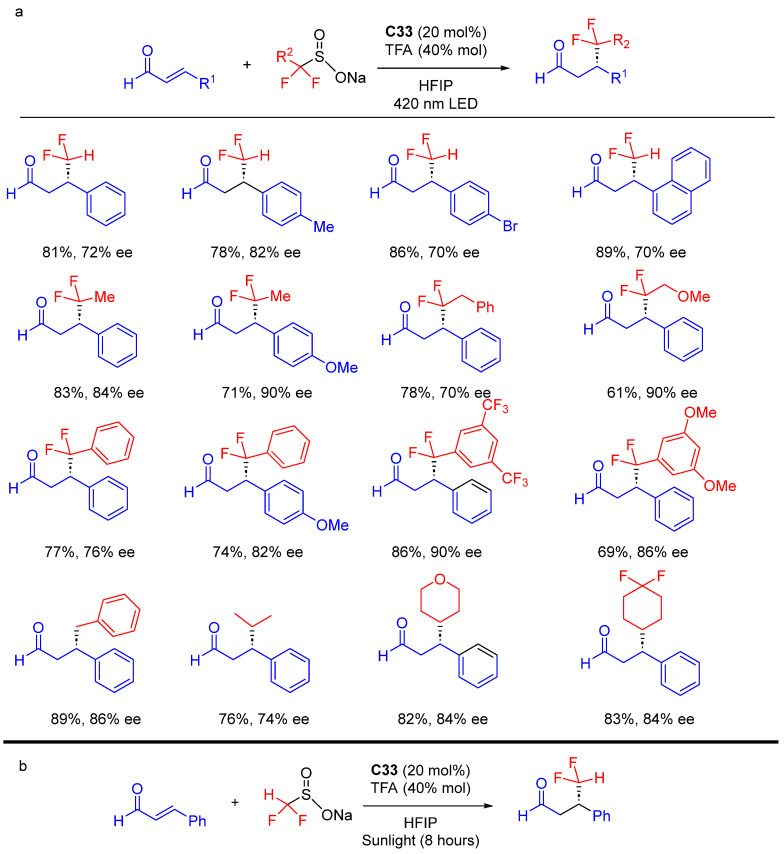
Visible-light-induced *gem*-difluoroalkylation of enals.

Alemán applied that idea for the synthesis of *gem*-difluoroalkylation of enals using alkyldifluorosulfinates in the presence of chiral catalyst **C33**. The reaction goes through the electron donor–acceptor complex of the iminium ion with sulfinate, followed by the visible-light-induced formation of the excited state intermediate. The interaction of the latter with sulfonate leads to sulfonyl radical formation, followed by SO_2_ extrusion and radical–radical recombination to arrive at a product with good yields and ee’s. The scope of the reaction could be extended to simple alkyl sulfinates (Scheme 116a). The reaction goes quite well, even under the natural sunlight irradiation; however, one has to take into account that the experiment was conducted in sunny Spain; the product was obtained in 71% yield and 72% ee (Scheme 116b).

**Scheme 117:**
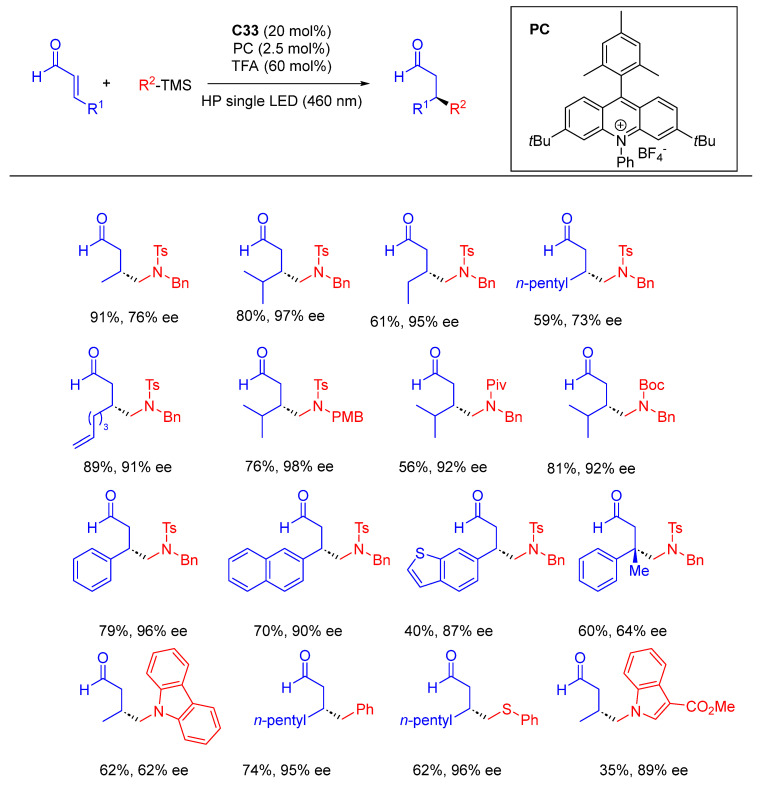
Alkylation of alkyl- and aryl-substituted enals with silyl radical precursors.

The lack of reactivity of enals substituted with an alkyl group in the β-position was overcome via the modification of the previous approach. Instead of relying on the activity of the enal’s excited state, the second partner of the reaction was transformed into the radical by means of an external photoredox catalyst. Then, it could react with the iminium ion in its ground state, regardless of the type of β-substituents. The broad applicability of this new approach was shown by the reactions of various alkyl- and aryl-substituted enals with silyl radical precursors [122]. In the presence of chiral catalyst **C33** and organic photoredox catalyst di-*t*BuMesAcr^+^BF_4_^-^, addition products were obtained with good yields and very high ee’s (Scheme 117).

**Scheme 118:**
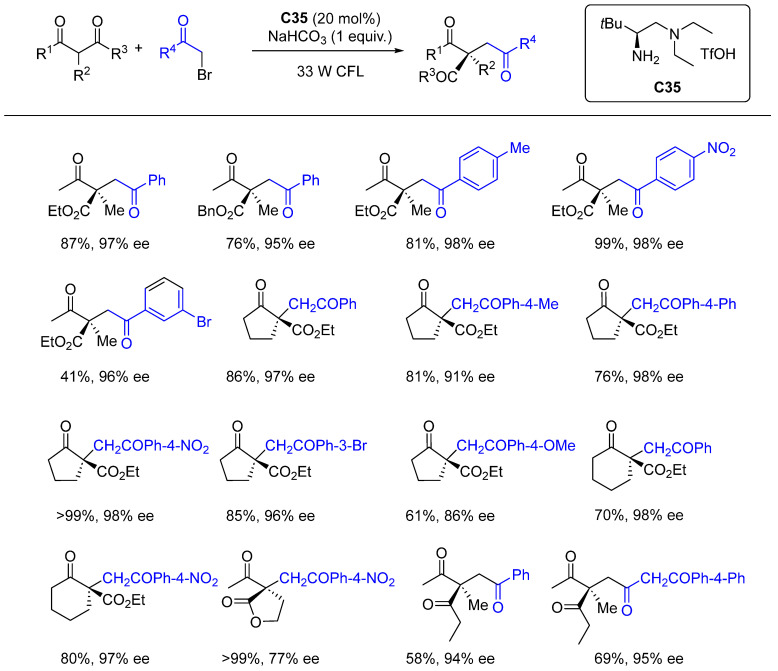
Addition of phenacyl bromide to β-ketocarbonyl compounds.

Melchiorre’s idea of a photoredox catalyst-free enamine catalysis was further extended to chiral primary amines as organocatalysts by Luo [123]. The addition of phenacyl bromide to β-ketocarbonyls provided a series of products with the quaternary center with good yields and high enantioselectivities. The sterically demanding primary amine **C35** was very efficient as an enamine-forming organocatalyst with further stabilization of the transition state by double hydrogen bonding to the remaining carbonyl of the substrate. The phenacyl bromide radical was formed in the CFL-induced photolysis. Some selected examples of the products are presented in Scheme 118.

## 5. Enzyme-Catalyzed Reactions

**Scheme 119:**
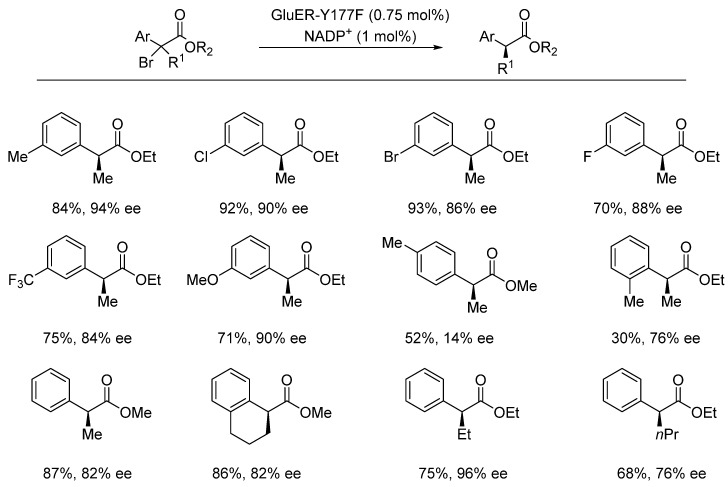
Enzymatic dehalogenation of α-bromoesters.

Enzymes are well known as excellent tools for asymmetric synthesis providing enantiomers with often extreme purity. However, many reactions which are highly valuable for organic chemists are not catalyzed by natural enzymes and some modification of the classical enzymatic synthesis approach is required. Even less popular is the application of enzymes for the enantioselective radical reactions. Hyster showed that flavine-dependent ‘ene’-reductases, known as EREDs, can be used as highly enantioselective chiral catalysts in radical chemistry. The first example discussed in this review is an enantioselective dehalogenation of α-bromoesters [124]. Hydrogen atom transfer (HAT) from flavine to reactant in the chiral environment of the enzyme GluER-Y177F obtained from mutated *Gluconobacter oxydans* is an enantiodetermining step. Several esters with a stereogenic center in the α-position were obtained with enantioselectivity varying from low to very high, and being highly dependent on the substrate structure (Scheme 119).

**Scheme 120:**
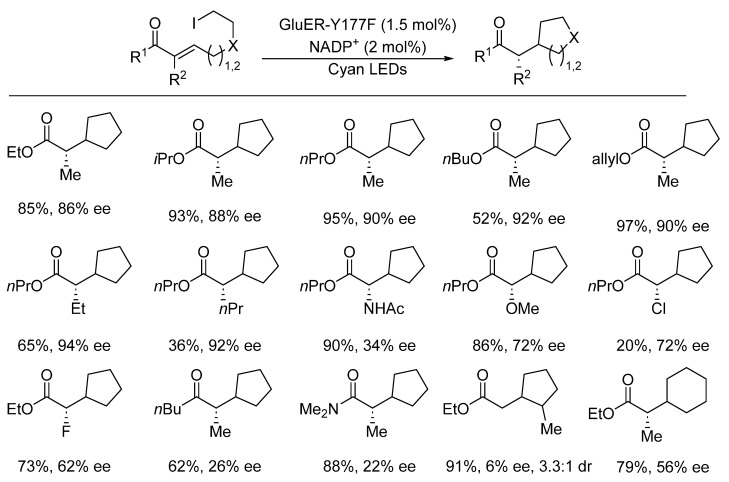
Enzymatic cyclization of unactivated iodides.

The same enzyme was used in the enantioselective cyclization of unactivated iodides (Giese reaction); however, in this case, an irradiation with cyan LEDs (497 nm) was required [125]. The stereoselectivity of the reaction was again highly dependent on the structure of the starting material and varied from nearly racemic to very high (Scheme 120).

**Scheme 121:**
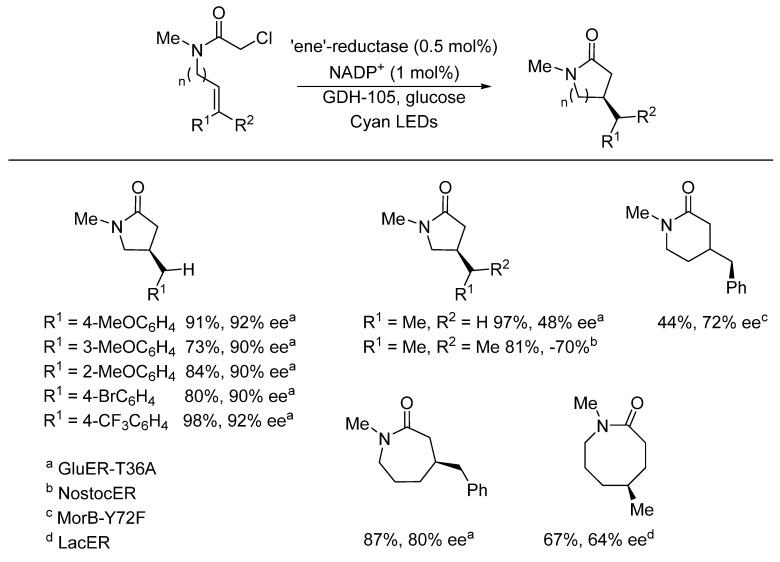
Cyclization of α-chloramides.

Another cyclization enzyme proposed by the Hyster’s group is the synthesis of lactams from α-chloramides [126]. The reaction is highly sensitive to the structure of the substrate used in this process; therefore, various types of ‘ene’-reductases (GluER T36A, NostocER, MorB-Y72F, LacER) were used to obtain the best results, However, the enantioselectivities were only moderate. Also, this reaction required irradiation with cyan LEDs (Scheme 121).

**Scheme 122:**
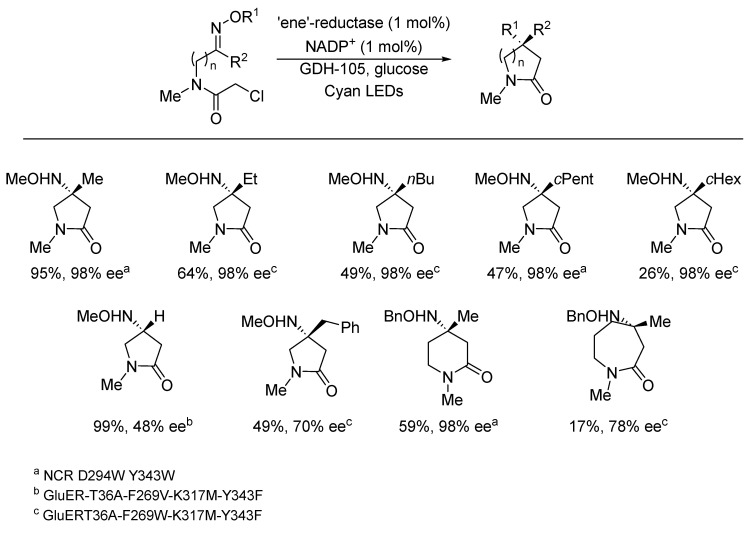
Cyclization of α-chloroamides bearing an oxime functionality.

Hyster’s group developed the very interesting cyclization of α-chloroamides bearing an oxime functionality [127]. Three very efficient engineered ‘ene’-reductases, NCR D294W Y343W, GluER-T36A-F269V-K317M-Y343F and GluERT36A-F269W-K317M-Y343F, were successfully used, yielding various chiral lactams, in several cases with excellent enantiomeric excesses (Scheme 122).

**Scheme 123:**
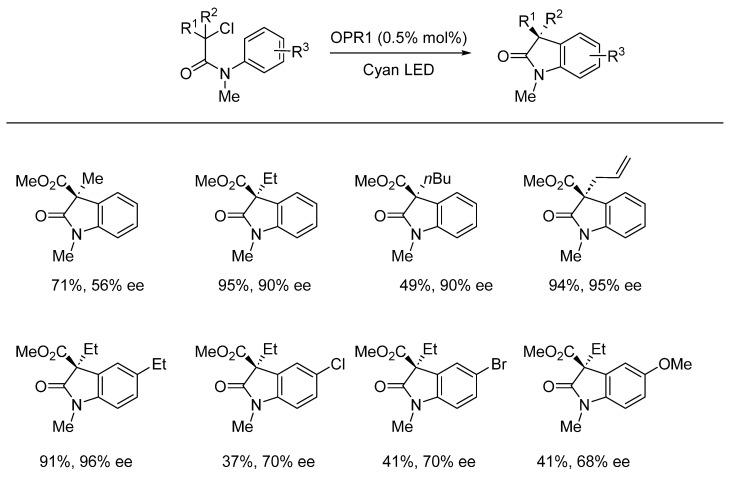
Cyclization of α-haloamides.

Flavine-dependent ‘ene’-reductase was used in the redox-neutral cyclization of α-chloroamides to oxindoles with moderate to high enantioselectivity [128]. Among several wild-type ERED’s tested, the best results were achieved for the 12-oxophytodienoate reductase (OPR1) and the cyan LED irradiation. The selected results are presented in Scheme 123.

**Scheme 124:**
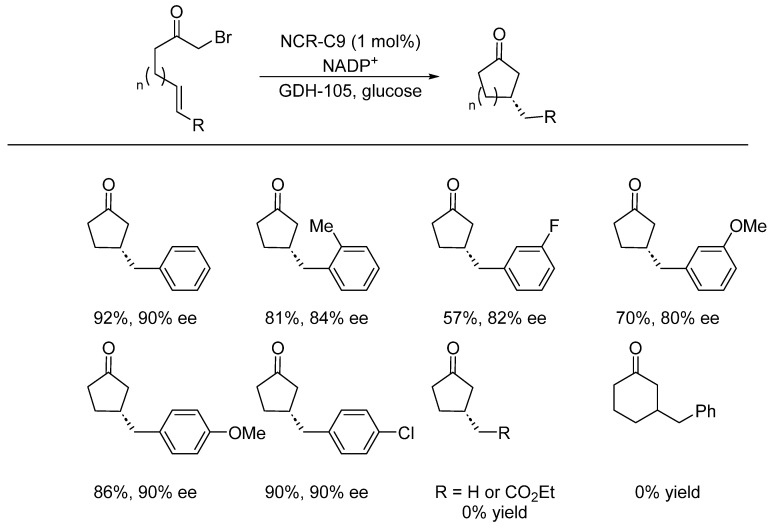
Synthesis of β-chiral cyclopentanones.

A mutated nicotinamide-dependent cyclohexanone reductase from *Zymomonas mobiles* was used for the synthesis of β-chiral cyclopentanones. Several wild-type enzymes were checked and did not deliver acceptable results; therefore, a new, artificial enzyme was developed by site saturation mutagenesis. The resulting NCR-C9 mutant secured high yields and stereoselectivities, as presented in Scheme 124.

**Scheme 125:**
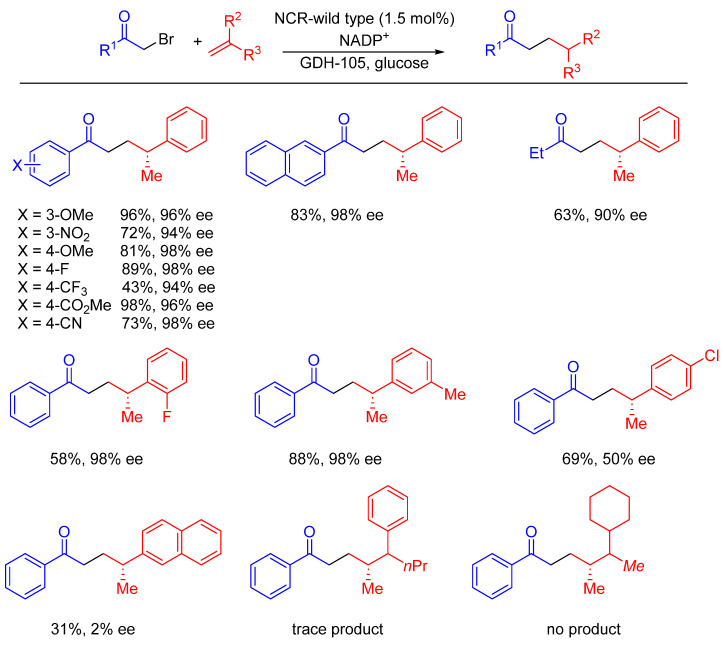
Hydroalkylation of α-methylstyrenes.

In the same paper, the authors described the enantioselective hydroalkylation reaction catalyzed by the wild-type NCR enzyme. This reaction of α-bromoacetophenones with α-methylstyrene goes via the stereoselective HAT process in the absence of a light source, as opposite to the method described by Zhang (vide infra). The stereoselectivity is good; however, the substrate choice seems to be restricted to α-methyl- or -ethylstyrene (Scheme 125).

**Scheme 126:**
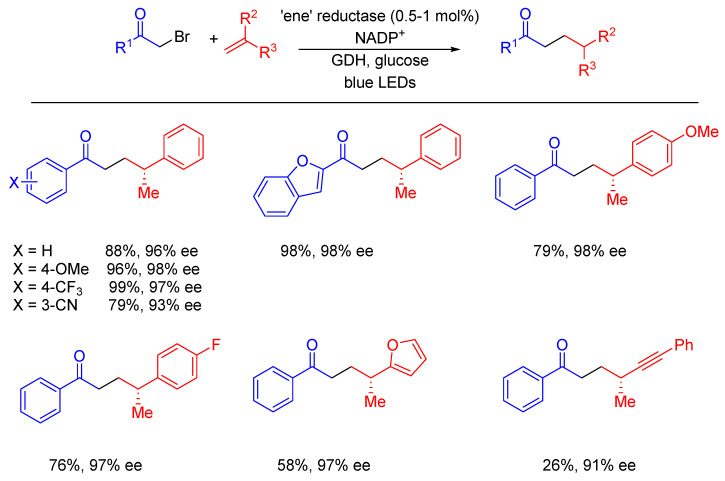
Hydroalkylation of alkenes.

As mentioned above, Zhang and co-workers presented similar hydroalkylation reactions. In this case, the best results, up to 98% ee (Scheme 126), were obtained when the reactions were catalyzed by OYE1, an Old Yellow Enzyme from *Saccharomyces cerevisiae* with irradiation using blue LEDs [129].

**Scheme 127:**
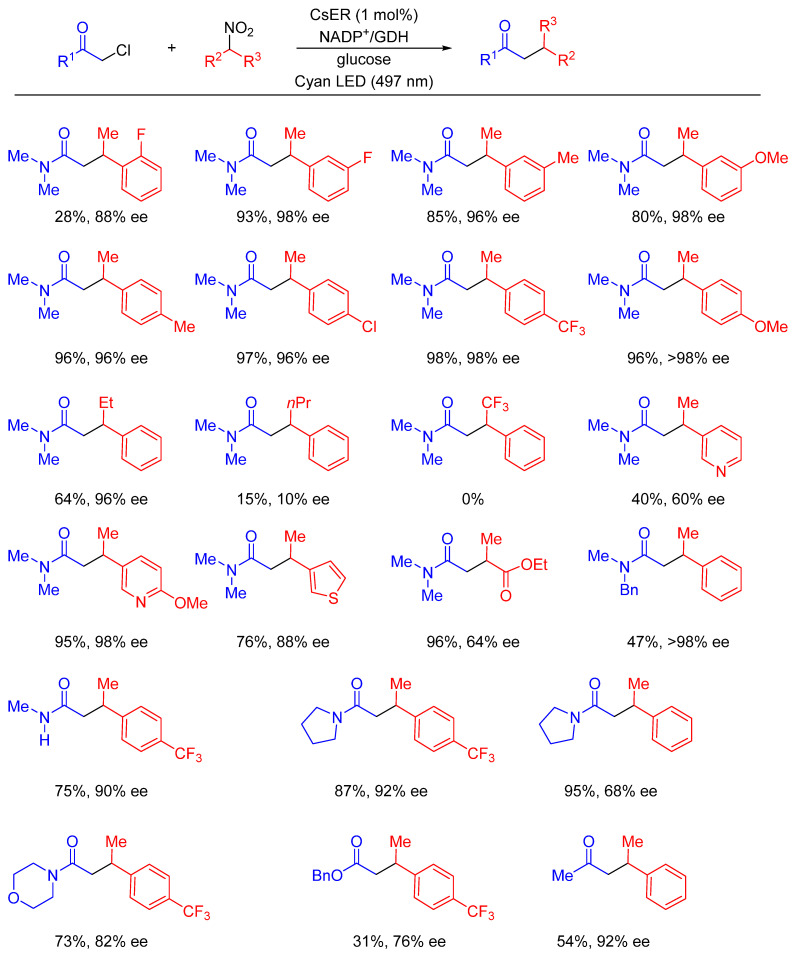
Cross-electrophile coupling of alkyl halides and nitroalkanes.

The wild-type flavine-dependent ‘ene’-reductase CsER from *Gluconobacter oxydans* was used for the challenging cross-electrophile coupling (XEC) of alkyl halides and nitroalkanes [130], leading to the enantioconvergent creation of a new Csp^3^-Csp^3^ bond. The formation of the product is not possible under classical photoredox metal catalysis and is unique for the biocatalysis. According to the proposed mechanism of the reaction, the alkyl radical formed from the halide reacts with the in situ-generated nitronate to form the new C-C bond. This nitro radical undergoes enzyme-mediated homolytic cleavage of the C-N bond and the newly formed alkyl radical is transformed into final product in the enzyme-controlled chiral space. The conversions and enantioselectivities of the reaction were in most cases very high, with the exclusion of bulky R^2^ substituents (Scheme 127).

**Scheme 128:**
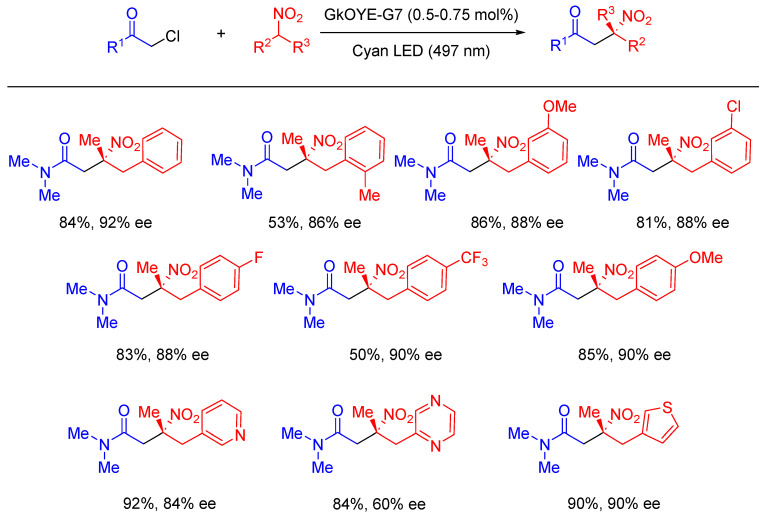
Coupling of alkyl halides and nitroalkanes leading to tertiary nitroalkanes.

The same type of substrates, which under CsER catalysis underwent Csp^3^-Csp^3^ coupling with the removal of the nitro group, was used in the synthesis of enantioenriched tertiary nitroalkanes [131]. The key modification of the reaction conditions was the use of the mutated Old Yellow Enzyme from Geobacillus kaustophilus (GkOYE); the mutation allowed for the enhancement of the enantioselectivity from a moderate 56% ee for the wild-type enzyme to, in some cases, 90% ee for the engineered one (GkOYE-G7). Selected examples are presented in Scheme 128.

**Scheme 129:**
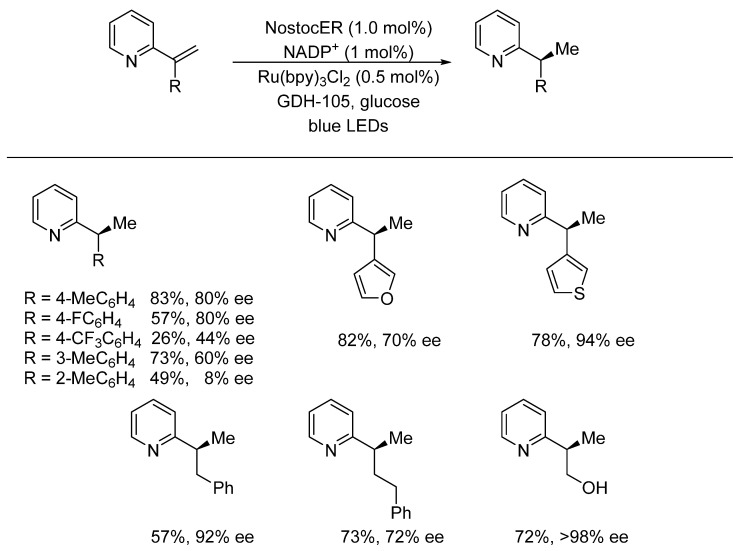
Hydrogenation of vinyl-substituted heteroaromatics.

The NostocER, the ‘ene’ reductase from *Nostoc punctiforme*, was applied to the hydrogenation of the 2-vinyl pyridines [132]. The process required the addition of ruthenium photosensitizer and irradiation with blue LEDs to afford products with good yields and high enantioselectivities (Scheme 129).

The use of enzymes in radical enantioselective synthesis is not limited to flavine-dependent ‘ene’ reductases. The enzyme catalysts derived from the cytochrome P450 proved to be very efficient tools in enzymatic radical synthesis. These enzymes are produced and function in bacteria, where they can be optimized by directed evolution for a broad spectrum of enantioselective reactions.

**Scheme 130:**
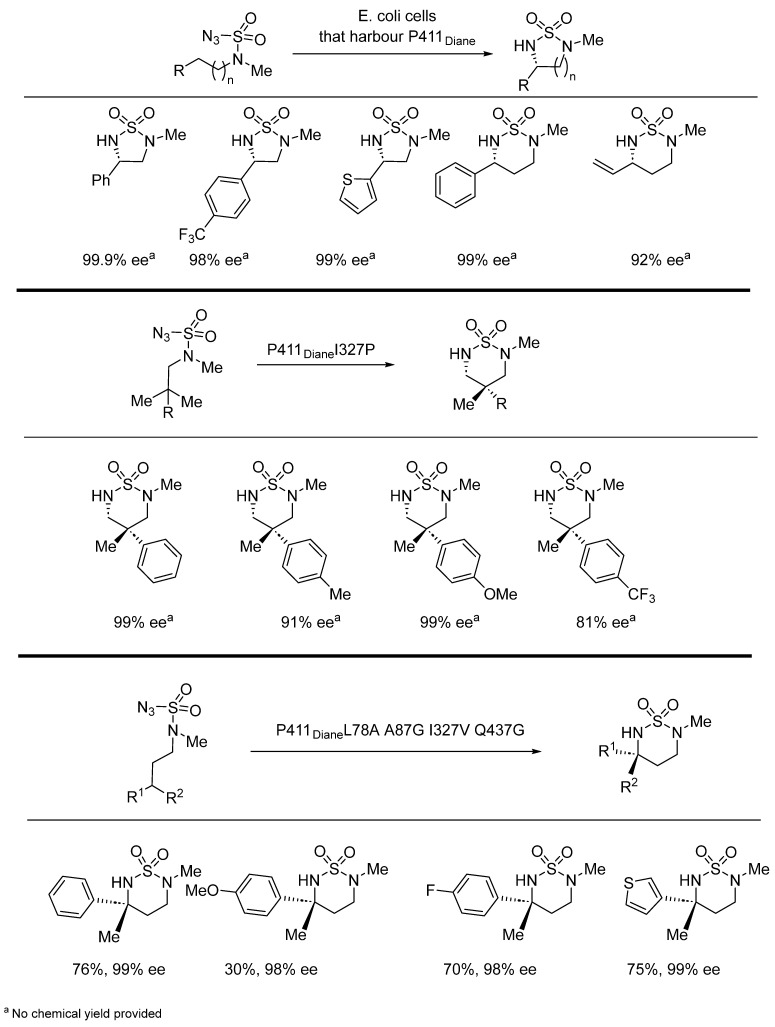
Amination of C(sp^3^)–H bonds.

Yang et al. described the amination of C(sp^3^)–H bonds which proceeds with excellent yield and enantioselectivities in the presence of cytochrome P411, a variant of P450 [133]. The catalysts were fine-tuned by the evolution of intact *Escherichia coli* cells that harbor P411 to deliver the best one for the amination process. Whole cells of *Escherichia coli* were used, which highly simplifies the synthetic procedures. The selected amination results are presented in Scheme 130.

Yang and Liu published important papers which deal with the mechanism of the P450-catalyzed reactions. These in-depth computational and experimental studies go beyond the scope of this review; however, we encourage interested researchers to read these works [134,135]. Within the same trend of mechanistic investigations, joined group of researchers from Universities of Queensland and Adelaide analyzed mechanistic aspects of P450-catalyzed alkene epoxidations [136].

**Scheme 131:**
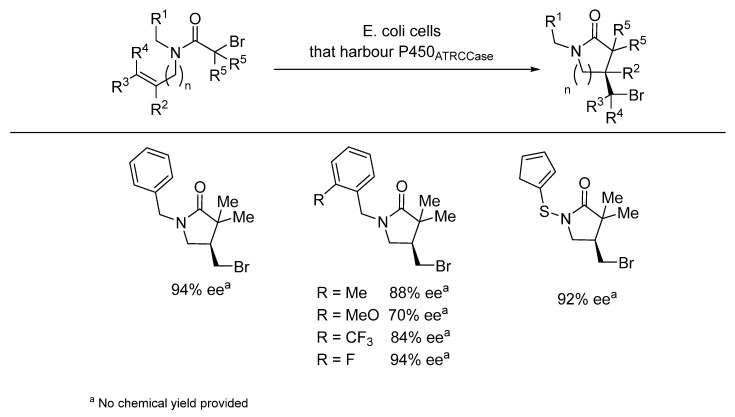
Stereodivergent atom-transfer radical cyclization.

Yang and co-workers developed another cyclization process catalyzed by engineered P450 enzymes [137]. They developed the atom-transfer radical cyclization (ATRC) of amides leading to respective lactams with low to very high enantioselectivity, depending on the substrate structure (Scheme 131).

**Scheme 132:**
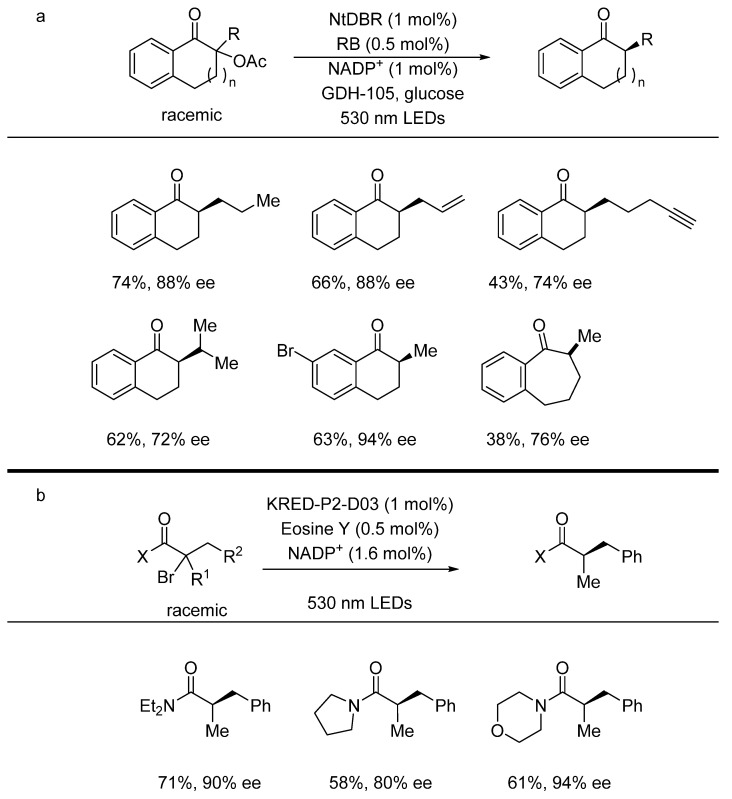
Enantioselective deacetoxylation (**a**) and debromination (**b**).

Biegasiewicz et al. described an enantioselective deacetoxylation of ketones by nicotinamide-dependent double-bond reductase (NtDBR) from *Nicotiana tabacum* in the presence of Rose Bengal (RB) as a photocatalyst and irradiation with visible light [138]. The reaction proceeded with good yields and good to high enantioselectivities (Scheme 132a). This approach appears to be more general and a tuning of the reaction conditions should allow for the effective use of other substrates. It was shown by enantioselective debromination of α-bromoamides in the presence of ketoreductase and Eosine Y (Scheme 132b).

**Scheme 133:**
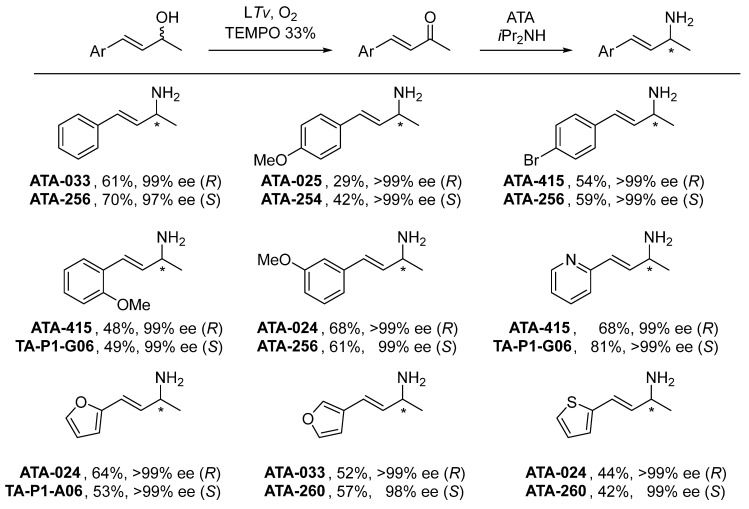
Sequential transamination of allylic alcohols.

The conversion of racemic allylic alcohols into enantioenriched allylic amines was based on two consecutive enzymatic processes: the oxidation of an alcohol with the laccase from *Trametes versicolor* and the oxyradical TEMPO to an α,β-unsaturated ketone, followed by transamination with amine transaminase (ATA) [139]. The sequential protocol was developed and the amines were obtained with moderate-to-good isolated yields and excellent enantiomeric excess, in some cases over 99% ee. Both enantiomers of the amines are available with the proper choice of ATA (Scheme 133).

**Scheme 134:**
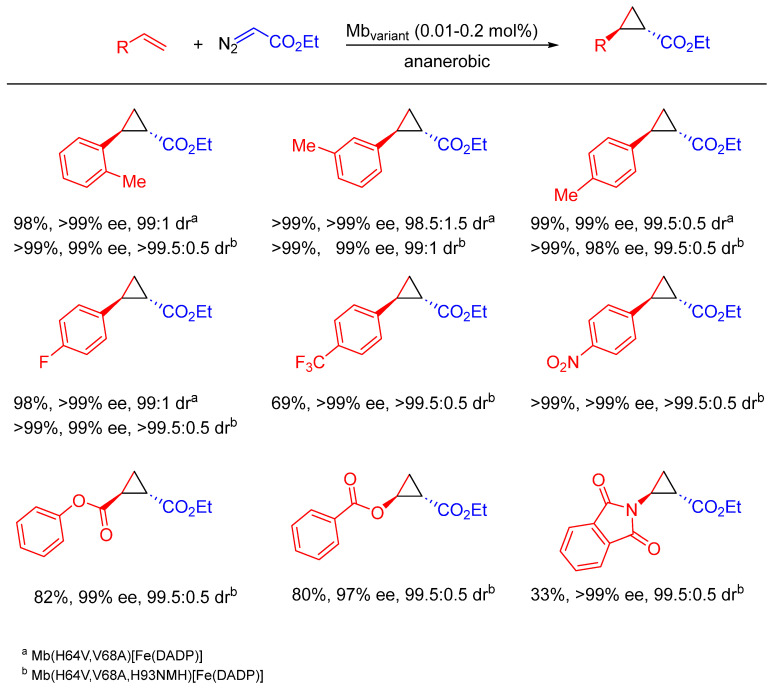
Myoglobin-catalyzed cyclopropanation.

The engineered metalloenzyme myoglobins (Mb) were shown to be very effective catalysts for the highly stereoselective cyclopropanation of various types of alkenes, as presented in Scheme 134 [140].

**Scheme 135:**
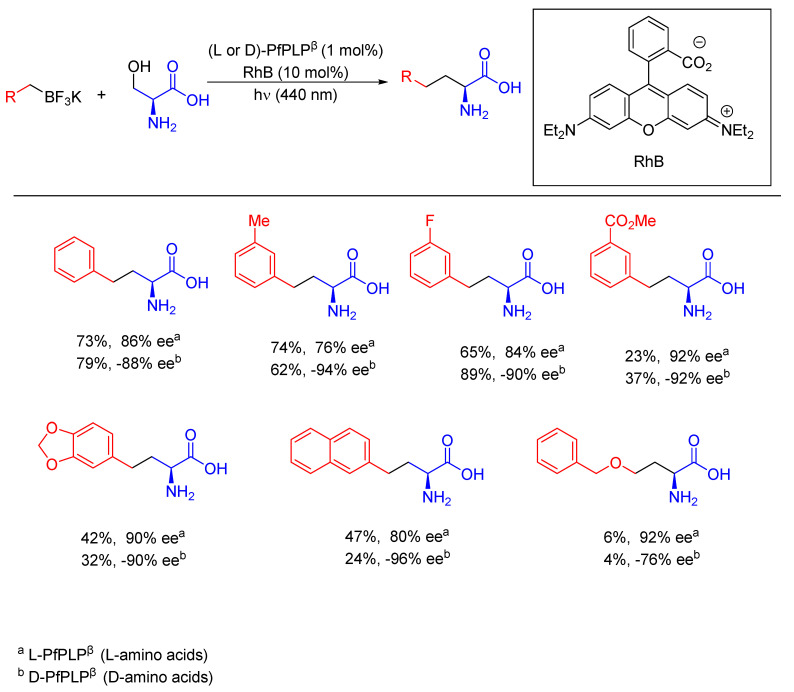
Synthesis of non-canonical amino acids.

Yang, Liu and co-workers developed a method in which the simultaneous use of a photoredox catalyst responsible for generating radicals and an enzyme creating a chiral environment was used for the synthesis of non-canonical amino acids [141]. The best results were obtained by the combination of rhodamine B (RhB) as the photocatalyst and the pyridoxal 5-phosphate (PLP) enzyme “2B9” variant of the *Pyrococcus furiosus* tryptophan synthase β subunit (l-PfPLP^β^) for l-amino acids and its single mutant E104G for d-amino acids (d-PfPLP^β^). Some selected examples from the broad library of products are presented in Scheme 135.

## 6. Conclusions

Enantioselective radical chemistry has been developing rapidly in recent years and new types of enantioselective reactions, hitherto known in the racemic version, are emerging. Undoubtedly, in the coming years, we will witness more and more new discoveries that will allow us to obtain new groups of compounds with very high enantiomeric excesses with greater and greater freedom.
